# Intervention strategies to improve adherence to treatment for selected chronic conditions in sub‐Saharan Africa: a systematic review

**DOI:** 10.1002/jia2.26266

**Published:** 2024-06-25

**Authors:** Siphamandla Bonga Gumede, John B. F. de Wit, Willem D. F. Venter, Annemarie M. J. Wensing, Samanta Tresha Lalla‐Edward

**Affiliations:** ^1^ Ezintsha, Faculty of Health Sciences University of the Witwatersrand Johannesburg South Africa; ^2^ Department of Interdisciplinary Social Science Utrecht University Utrecht the Netherlands; ^3^ Centre for Social Research in Health UNSW Sydney New South Wales Australia; ^4^ Department of Medical Microbiology University Medical Center Utrecht Utrecht the Netherlands; ^5^ Ndlovu Research Consortium Elandsdoorn South Africa

**Keywords:** adherence, antiretroviral therapy, anti‐hypertensive medication, anti‐diabetic medication, systematic review, Africa

## Abstract

**Introduction:**

Evidence‐based intervention strategies to improve adherence among individuals living with chronic conditions are critical in ensuring better outcomes. In this systematic review, we assessed the impact of interventions that aimed to promote adherence to treatment for chronic conditions.

**Methods:**

We systematically searched PubMed, Web of Science, Scopus, Google Scholar and CINAHL databases to identify relevant studies published between the years 2000 and 2023 and used the QUIPS assessment tool to assess the quality and risk of bias of each study. We extracted data from eligible studies for study characteristics and description of interventions for the study populations of interest.

**Results:**

Of the 32,698 total studies/records screened, 2814 were eligible for abstract screening and of those, 497 were eligible for full‐text screening. A total of 82 studies were subsequently included, describing a total of 58,043 patients. Of the total included studies, 58 (70.7%) were related to antiretroviral therapy for HIV, 6 (7.3%) were anti‐hypertensive medication‐related, 12 (14.6%) were anti‐diabetic medication‐related and 6 (7.3%) focused on medication for more than one condition. A total of 54/82 (65.9%) reported improved adherence based on the described study outcomes, 13/82 (15.9%) did not have clear results or defined outcomes, while 15/82 (18.3%) reported no significant difference between studied groups. The 82 publications described 98 unique interventions (some studies described more than one intervention). Among these intervention strategies, 13 (13.3%) were multifaceted (4/13 [30.8%] multi‐component health services‐ and community‐based programmes, 6/13 [46.2%] included individual plus group counselling and 3/13 [23.1%] included SMS or alarm reminders plus individual counselling).

**Discussion:**

The interventions described in this review ranged from adherence counselling to more complex interventions such as mobile health (mhealth) interventions. Combined interventions comprised of different components may be more effective than using a single component in isolation. However, the complexity involved in designing and implementing combined interventions often complicates the practicalities of such interventions.

**Conclusions:**

There is substantial evidence that community‐ and home‐based interventions, digital health interventions and adherence counselling interventions can improve adherence to medication for chronic conditions. Future research should answer if existing interventions can be used to develop less complicated multifaceted adherence intervention strategies.

## 1 INTRODUCTION

Patients on treatment for chronic conditions face multiple barriers to adherence, and no single intervention is deemed sufficient to ensure that high levels of adherence to treatment are maintained [1]. There remains a need to strengthen and tailor different intervention strategies to different barriers to adherence for chronic conditions [1].

In the efforts to address adherence to treatment for chronic conditions; behavioural and psychological factors, education, integrated care and patient self‐management interventions have been explored [2, 3]. This includes behavioural rehabilitation provided by health providers to patients, integration of psychosocial support within health programmes and patients’ knowledge about the medication and their overall satisfaction with the treatment [4, 5, 6]. Other studies have recommended telephonic counselling and text messaging or reminders (mobile health/mhealth), packaging/medication boxes, home visits, drug‐level monitoring and consistent clinical monitoring of patients [4, 6, 7]. Studies focusing on adherence to antiretroviral therapy (ART) for human immunodeficiency virus (HIV) have further emphasized the importance of compliance with standard treatment guidelines (monitoring and reporting of health information [data] to promote appropriate medicine use) [8, 9, 10]. Interventions that use mobile technology (mhealth) have the potential to facilitate self‐management, education and support, unfortunately, mhealth applications have been limited in sub‐Saharan Africa (SSA), and they have had mixed effects on controlling non‐communicable diseases (NCDs) [11, 12].

Although numerous reviews have evaluated adherence interventions [12, 13, 14, 15, 16], few have undertaken a comparative analysis of adherence to medication for various conditions, notably HIV, hypertension (HPT) and diabetes mellitus (DM). While HIV remains the leading cause of death especially in young and middle‐aged adults in SSA, the burden of NCDs, particularly HPT and DM, has increased rapidly in recent years [17, 18, 19]. There has been a surge in the burden of NCDs in SSA over the past two decades, rising from 24% in 2000 largely due to challenges in the implementation of preventative and control measures including screening, diagnosis and care [20, 21, 22]. Therefore, understanding adherence to related medication is a priority issue. This review assesses treatment adherence interventions for HIV, HPT and DM in SSA, which provides valuable comparisons and context to adherence intervention strategies for chronic conditions in SSA. We sought to understand evidence‐based approaches to support adherence to treatment for HIV, HPT and DM.

## 2 METHODS

This systematic review has been designed and reported according to the Preferred Reporting Items for Systematic Reviews and Meta‐Analysis (PRISMA) [23] (Supplementary Material 1), following the registered protocol on the International Prospective Register of Systematic Reviews (PROSPERO) (registration number: CRD42019127564) [24]. The study used Population (P), Interventions (I), Comparisons (C) and Outcomes (O) (PICO) criteria as the search strategy tool.

### 2.1 Eligibility criteria

All studies assessing the impact of adherence interventions for ART, anti‐hypertensive medication and anti‐diabetic medication in SSA that were conducted or published between 01 January 2000 and 30 November 2022 were considered for inclusion. Studies were excluded if the study setting was not SSA, if papers were written in any other language than English, if the health condition for which adherence to medication was assessed was not HIV, HPT or DM, and if the study was published before the year 2000 (Table 1).

**Table 1:** Methodological aspect of the systematic review

Criteria for study inclusion	Components details
Population (P)	Patients/participants with selected chronic conditions (HIV, HPT, DM) in SSA
Intervention (I)	All interventions listed/described as medication adherence interventions or strategies for the conditions of HIV, HPT, DM
Comparisons (C)	Standard of care and other adherence interventions reported in the relevant study
Outcome (O)	There is no preferred measurement for reporting. For this review, we included studies that reported any quantitative measure of medication adherence including self‐reported adherence using a defined threshold, pill count using a defined threshold, change in CD4^+^ lymphocyte count and HIV‐1 RNA (viral load), mean change in systolic blood pressure (SBP) and/or diastolic blood pressure (DBP) measured at certain intervals, retention in care and LTFU, weight, a reduction in HbA1C level, improved specific self‐efficacy, mean change in weight, mean waist circumference, mean HbA1c and medication adherence using Morisky Medication Adherence Scale (MMAS‐8). Effects on adherence behaviour and the changes in health outcomes.
Setting	All studies from SSA only were considered for the review.
Language	English
Publication date and study duration	The year 2000−2023
Publication status	All the documented studies were considered and included for review. This includes peer‐reviewed (i.e. papers, manuscripts and abstracts) and dissertations or theses.

### 2.2 Data collection

We conducted a systematic data search using several electronic databases, including PubMed Web of Science, CINAHL, Scopus and Google Scholar, between 01 August 2021 and 29 February 2024 (including the period when the analysis was updated: 08 February 2024−29 February 2024). These dates included repeat searches in case of article publications occurring after the initial search. We also reviewed citations and bibliographies of other related reviews to identify additional relevant material.

The search terms were adjusted to suit the database being searched. An inventory with the database searched, the corresponding search criteria used, the date when the searches were conducted and the results were all maintained (Supplementary Material 2_search term strategy). Two other reviewers (Lisa Noordman and Marit Wiltink) ran the searches separately for comparison. The comparison entailed a number of articles showing as a result of each search (search hits). Small differences were observed and attributed to different dates on which databases were accessed and/or searched by the reviewers. In this case, results from each reviewer were merged and de‐duplicated.

Three reviewers (S.B.G., Lisa Noordman and Marit Wiltink) independently conducted title and abstract screening. The screening results were compared for each reviewer to identify any discrepancies. Discrepancies in the screening results were discussed between the three reviewers. In cases where an agreement on inclusion could not be reached, a fourth reviewer (S.T.L.‐E.) made the final decision.

From the database search engines, data were imported into the Rayyan electronic tool, a free web‐tool designed to help synthesize data for systematic reviews, scoping reviews and literature reviews [25].

### 2.3 Study selection

**Figure 1:**
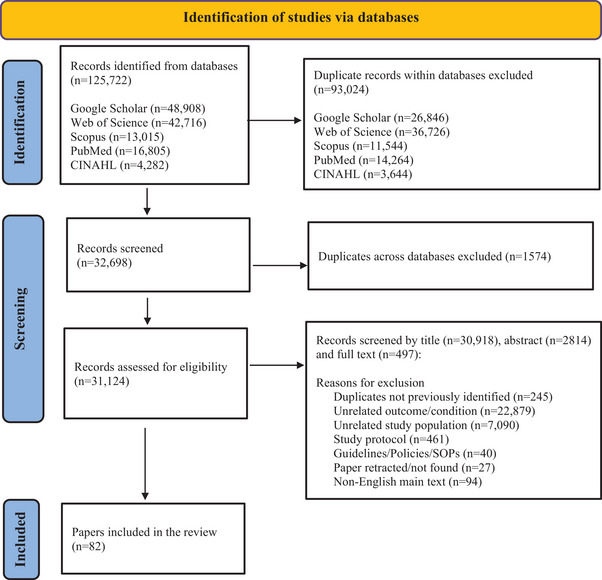
Flow chart—identification of studies via databases.

The reviewers performed title and abstract screening using a predefined list of inclusion and exclusion criteria (Table 1 and Figure 1). In case the article was not specific or clear enough, especially in terms of intervention type and outcome measures, screening was discussed by all the reviewers and S.T.L.‐E. made the final decision. Multiple studies of the same cohort were included if different outcomes were studied.

### 2.4 Data extraction

Data were extracted using a standardized form. The following details were extracted: study title, first author, publication year, type, study duration, country, population, study geographical setting, study design, sample size, intervention description, details on age, sex, condition (HIV, HPT, DM), outcome measures and main results.

### 2.5 Quality assessment

For all studies in the systematic review, assessments of quality and risk of bias were performed using the Quality in Prognostic Studies (QUIPS) tool (Supplementary Material 3) [26]. The risk of bias was assessed as low risk, moderate risk or high risk for each of the following domains: study design, participant selection, study sample size, descriptions of outcomes and description of interventions.

### 2.6 Data analysis

For each study included in the review, the following characteristics were recorded: authors, year of publication, country where the study was conducted, study duration, study design, study site/setting, study population/sample, sample size, sex of participants, age of participants, health condition studied, intervention assessed, outcomes analysed and main findings. Adherence interventions were described according to the type of intervention and implementation setting.

## 3 RESULTS

### 3.1 Description of included studies

A total of 32,927 records were screened and after the removal of duplicates, a total of 30,918 records remained and their titles and abstracts were screened for inclusion. Of the 30,918 records, 2814 were further screened for inclusion by abstract. The full‐text screening of potentially eligible articles was subsequently done in 497 records. The reasons for the exclusion of papers are noted in Figure 1. In total, 82 articles [27, 28, 29, 30, 31, 32, 33, 34, 35, 36, 37, 38, 39, 40, 41, 42, 43, 44, 45, 46, 47, 48, 49, 50, 51, 52, 53, 54, 55, 56, 57, 58, 59, 60, 61, 62, 63, 64, 65, 66, 67, 68, 69, 70, 71, 72, 73, 74, 75, 76, 77, 78, 79, 80, 81, 82, 83, 84, 85, 86, 87, 88, 89, 90, 91, 92, 93, 94, 95, 96, 97, 98, 99, 100, 101, 102, 103, 104, 105, 106, 107, 108] were found to be eligible and data were then extracted (Supplementary Material 4).

Of the 82 articles, 74/82 (90.2%) were peer‐reviewed journal articles, 7/82 (8.5%) were dissertations and 1/82 (1.2%) were paper abstracts. Fifty‐eight papers reported studies of interventions to promote adherence to ART, six were anti‐hypertensive medication related, 12 were anti‐diabetic medication related and six focused on more than medication for more than one condition. The studies included reported findings on a total of 58,043 participants. The mean sample size was 735 participants (range: 10–10,136). The median age of participant samples was between 30 and 40 years and about two‐thirds of the study participants were women. Almost half of the included studies (39/82, 47.6%) were conducted in the southern African region, with a total of 35,849/58,043 participants (61.8%), and East Africa (31/82, 37.8%) with a total of 18,121/58,043 participants (31.2%) (Supplementary Material 4). The remaining 12/82 (14.6%) studies with 4073/58,043 (7.0%) participants were conducted in other parts of the sub‐Saharan African region (including West Africa and central Africa).

Of the included papers, 34/82 (41.5%) reported findings of evaluation studies, including randomized controlled trials (31/34, 91.2%) and quasi‐experimental studies (3/34, 8.8%), 19/82 (23.2%) reported findings of correlational studies, notably cross‐sectional surveys (11/19, 57.9%), and cohort studies (8/19, 42.1%), 17/82 (20.7%) reported findings of qualitative research and 5/82 (6.1%) were mixed method studies. The remaining 8/82 (9.8%) papers included various study designs such as non‐described descriptive studies. Data were collected between 2003 and 2023 and the year of study publications ranged from 2006 to 2023.

A total of 54/82 (65.9%) reported improved adherence based on the described study outcomes, 13/82 (15.9%) did not have clear results or defined outcomes and 15/82 (18.3%) reported no significant difference between studied groups.

Of the studies reporting improved medication adherence, 40/54 (74.1%) were related to ART, 2/54 (3.7%) were related to HTN, 9/54 (16.7%) were related to DM and 3/54 (5.6%) were related to more than one health condition. (Supplementary Material 4).

The 82 publications described 98 unique interventions (some studies described more than one intervention). Of the 37 community‐ and home‐based reported interventions, peer treatment supporters (14/37, 37.8%), nutrition support (7/37, 18.9%) and community‐based social network support (10/37, 27.4%) were the most described community‐ and home‐based interventions. Of the total mhealth‐based interventions, SMS reminders at a regular interval (10/19, 55.6%) was the most described mhealth‐based intervention (Table 2).

**Table 2:** Description of adherence intervention types

Intervention type	Definition	ART	Anti‐hypertensive medication	Anti‐diabetic medication
**Community‐ and home‐based interventions**				
Community based‐adherence support (CBAS) with home visits	Provision of adherence support through home visits by a community health worker or peer volunteer. Home visitors are involved in a variety of activities such as food ration provision basic clinical assessments and patient referrals, ART delivery, and providing DOT and pill counts.	2	0	0
Multi‐component health services‐ and community‐based programme	Interventions involve a facility‐designated health worker who connects patients to facility‐level services. Responsibilities of the worker have included home visits, patient monitoring and tracing, and counselling.	4	0	0
Peer treatment supporters	Involving other persons within a patient's social circle in assisting that patient with their treatment. Examples of responsibilities of the treatment supporter include providing Direct Observed Therapy (DOT) service, social support, bonding, bridging or linking social networks, clinical site mediation, and providing regular follow‐up reminders for the next appointment attendance.	9	2	3
Community‐based social network support	Support at the community level is designed to be delivered to a group. Includes interventions involving a patient's extended social network in the patient's treatment.	6	2	2
Nutrition support	Providing supplementary nutritional support either at the individual or household level. Can come in the form of food rations or nutrition education.	7	0	0
**Total**		**28**	**4**	**5**
**Digital health/mhealth/ehealth interventions**				
SMS reminders sent at regular intervals	Regular delivery of SMS messages aimed at directly or indirectly reminding patients to adhere to their medication.	8	1	1
SMS reminders triggered by adherence monitors	SMS messages were sent if an electronic adherence monitoring device was not opened within 30 minutes of the scheduled dose time.	1	0	0
IVR or phone calls for reminders	Interactive voice response or regular phone calls delivering messages on medication adherence and other HIV/ART‐related topics, as well as appointment reminders. Some also sent non‐interactive, SMS picture messages to remind patients of dosage adherence.	1	0	0
Smartphone application	Medication reminders and adherence tracking, refill and appointment reminders, leaderboard and adherence points, social support through discussion forms, peer‐to‐peer support and community‐based resources list	2	0	0
eHealth: adherence monitor device	A device enabling healthcare professionals or researchers to interpret activities of the patient/participant in the context of adherence and inform better decision‐making and as an intervention and tool for the patient to aid self‐management and improve adherence.	2	0	0
**Total**		**14**	**1**	**1**
**Adherence counselling**				
Individual counselling	Adherence to educational and/or counselling interventions delivered in a one‐on‐one setting. Sessions are often led by trained health professionals or lay counsellors (including direct social support).	7	2	5
Group counselling	Adherence to educational and/or counselling interventions delivered in a group setting. Includes social support groups. Sessions are often led by trained professionals or lay counsellors and delivered through a set curriculum or informed by a psychosocial theory/practice.	5	1	0
Individual plus group counselling	Interventions with both individual and group counselling components (including educational and/or counselling delivered in a one‐on‐one or group setting)	6	0	0
Adherence counselling: SMS or alarm reminders plus individual counselling	Individual adherence counselling combined with regular reminders. Reminders could come in the form of either an alarm device programmed around dosage times or regular SMS messages, sent at times independent of the dosage schedule.	3	0	0
**Total**		**21**	**3**	**5**
**Health service interventions**				
Decentralization: decentralized medication delivery (DMD)	Decentralization comprises clinically stable ART patients who meet at facilities or community locations in groups of up to 30 every 2−3 months to receive group counselling, have a brief symptom screen and receive pre‐packed medications. DMD comprises prepacking and distribution of medications to pick‐up points, which are at locations other than the clinic pharmacy. Patients only need to come to the clinic on a 6‐monthly basis for a clinical exam and rescripting.	1	0	0
Health system strengthening programme	A well‐functioning health system working in harmony is built on having trained and motivated health workers, a well‐maintained infrastructure, and a reliable supply of medicines and technologies, backed by adequate funding, strong health plans and evidence‐based policies.	1	0	0
Integration of services	Provision of integrated care with standard vertical care delivered separately for people with HIV, diabetes or hypertension.	1	1	1
**Total**		**3**	**1**	**1**
**Incentive intervention (behaviour change mechanism)**				
Voucher	Vouchers are cash or gifts with a definitive value provided to study participants. The voucher incentive interventions are offered and designed by the researchers or investigators to reward participants/patients for achieving a certain goal and encourage team members to exceed their goals.	1	0	0
**Total**		**1**	**0**	**0**
**Drug optimization**				
Transition in medication	The adoption of better drug regimens to improve treatment adherence, viral suppression and quality of life of people living with the specified condition. These benefits could reduce pressures on health systems as lower rates of viral failure on new treatments could reduce the risk of HIV drug resistance (HIVDR) and HIV transmission. In addition, the transition to new lower‐cost antiretroviral (ARV) drugs could provide significant savings for national health budgets worldwide.	2	0	0
**Total**		**2**	**0**	**0**
**Educational intervention**				
Active visualization device	A device that delivers or provides health information that could be particularly useful in educating patients about the specific condition or related treatment.	1	0	0
Health education/knowledge	Any combination of learning experiences designed to help individuals and communities improve their health, by increasing their knowledge or influencing their attitudes.	1	1	2
Health literacy	The achievement of a level of knowledge, personal skills and confidence to take action to improve personal and community health by changing personal lifestyles and living conditions.	0	0	1
**Total**		**2**	**1**	**3**
**Other**				
Intervention counting pills	Monthly clinic‐based pill counts	1	0	0
The food intervention programme: food by prescription	A programme that provides food and nutritional care to malnourished HIV‐positive individuals as a therapeutic and supplementary feeding package at health facilities.	1	0	0
**Total**		**2**	**0**	**0**
**Overall total**		**73**	**10**	**15**

*Note*: The categorization of interventions was adapted from a study by Ridgeway et al. [13].

Other interventions included adherence counselling (29/98, 29.6%), health service interventions (3/98, 3.1%) (which included decentralization of services and health systems strengthening), incentive intervention (voucher) (1/98, 1.0%) and drug optimization (transition in medication) (2/98, 2.0%). Of the 98 unique intervention strategies, 13 (13.3%) were multifaceted (4/13 [30.8%] multi‐component health services‐ and community‐based programmes, 6/13 [46.2%] included individual plus group counselling and 3/13 [23.1%] included SMS or alarm reminders plus individual counselling) (Table 2).

### 3.2 Risk of bias

Risk of bias was measured using the QUIPS tool (Supplementary Material 3), based on the study design, participant selection, study sample size, descriptions of outcomes and description of interventions. Of the 34 papers (including randomized control trials and quasi‐experimental), 28/34 (82.4%) scored a low risk of bias, 4/34 (11.8%) a moderate risk of bias and 2/34 (5.9%) reported a high risk of bias. Out of the 20 correlational papers (cohort and cross‐sectional studies), 15/20 (73.7%) scored a low risk of bias and 5/20 (26.3%) scored a moderate risk of bias. From the 17 qualitative studies, only 2/17 (11.8%) scored a low risk of bias, 11/17 (64.7%) scored a moderate risk of bias and 4/17 (23.5%) scored a high risk of bias. From the other study designs (mixed and non‐described descriptive methods), 6/11 (54.5%) scored a low risk of bias, 3/11 (27.3%) scored a moderate risk of bias and 2/11 (18.2%) scored a high risk of bias. Overall, 51/82 (62.2%) scored a low risk of bias, 23/82 (28.0%) a moderate risk of bias and 8/82 (9.8%) a high risk of bias. Most papers scored well on participant selection, study design, outcome measurement, intervention measurement, data collection methods and data analyses.

## 4 DISCUSSION

There has been a significant increase in the number of studies implementing and evaluating interventions aimed at promoting adherence to chronic conditions. This systematic review combines the available evidence from a large number of studies to identify a range of adherence interventions aimed at promoting adherence to ART among people living with HIV, anti‐hypertensive medication and anti‐diabetic medication. The majority of adherence interventions described in this review were ART‐related. This is consistent with health programmes in most sub‐Saharan African countries that have placed more focus on HIV programmes as compared to HPT and DM‐related programmes, but different to most high‐income countries whose focus has been balanced across the three chronic conditions [8, 109–111].

Individual‐related characteristics described in the reviewed studies demonstrated that almost two‐thirds were females and of middle age (30−40 years). The individual‐specific characteristics identified may represent the demographic profile of patients in chronic treatment programmes, particularly those receiving HIV care in the SSA [112, 113].

The community‐based adherence interventions highlighted an important link between primary healthcare facilities or services and the communities, demonstrated integration of treatment and patient care, and decentralization of chronic care to the communities [114, 115]. Community‐ and home‐based adherence interventions such as peer treatment support meet the rising need associated with overall chronic care, where due to the real shortage of healthcare workers and the growing caseload of people needing care, professional workers’ roles are increasingly limited to medical and nursing tasks in health facilities [116]. This review provides evidence of the efficacy of community‐ and home‐based adherence support strategies, but more focus should be on their acceptability and cost‐effectiveness.

Mobile health is increasingly being explored for health promotion [117] and was also used in adherence‐promoting interventions identified in this review, to deliver educational and behavioural components, either singly or in combination. The majority of mhealth‐related medication adherence interventions described in this review reported improved adherence using specified outcome measurements. Most mobile health interventions were used to educate, remind or provide advice to patients. These technologies enabled the collection and transfer of patient‐specific data/information across to different professionals, who could then deliver tailored feedback and reminders to the patients. The increasing advancement in technology and related benefits received by patients from healthcare providers without presenting at a health facility is an appealing prospect. Furthermore, mhealth interventions could have a greater reach, and better adoption and implementation; thus having a greater positive heath impact [117, 118]. However, more research is needed to establish the sustainability of such interventions and to evaluate how mhealth interventions can be useful in the short and long term in promoting adherence to medications.

The interventions described in this review were primarily directed at patients and ranged from adherence counselling including both individual and group counselling to more complex interventions such as mhealth interventions which took into consideration patients’ abilities to use digital technology and preferences in addition to educating and aiding them to adhere to medication. Some of the interventions employed a combination of interventions, for example adherence intervention consisting of a combination of educational, behavioural or affective strategies. Behavioural and effective strategies, which are increasingly being used in adherence support interventions, range from adherence aids (such as medication administration aids), to motivational interviewing [119, 120].

Individual and group counselling adherence interventions could be regarded as being more patient‐centred; however, their impact depends on the extent to which patients’ or individuals’ psychosocial needs are taken into consideration. This includes attitudes towards the health condition, cultural barriers, social concerns (such as perceived stigma) and cognitive abilities. These needs have been recognized in recent years as important predictors of optimal adherence to treatment and should be considered in any development of adherence interventions for chronic conditions [121].

The role of healthcare service‐related interventions on medication adherence has been emphasized, particularly in cases of chronic diseases [122], though their impact is difficult to measure and has often been found to lack consistency [122, 123]. More adherence interventions have also addressed healthcare services‐related factors impacting adherence, as was seen in this review. These include patient‐related, condition‐related and medication‐related factors. For example, this review reported that adopting and using better ART drug treatment improved adherence to medication. In addition, greater emphasis on task‐shifting and decentralization of services improved medication adherence and is, therefore, worthy of further investigation.

This review also reported improved medication adherence and retention in care for participants who received cash vouchers during the study period. The early effects on adherence and retention were sustained in the cash groups after the intervention was complete. Although this intervention improved adherence, the effect of such interventions should be considered along with other tested interventions as part of a comprehensive package of support during the treatment journey. A larger‐scale impact evaluation to determine the effectiveness of cash support on cost‐effectiveness, and issues related to sustainability, also needs consideration.

Overall, any intervention designed to influence human behaviour, such as modifying medication adherence in patients with chronic conditions, would be more successful if multiple factors that aid the change in human behaviour are addressed. Combined interventions comprise different components, which may act both independently and inter‐dependently, to address the changes needed, and may be more effective than using a single component in isolation [124]. However, the complexity involved in designing, implementation and replicating combined interventions often complicates the practicalities of such interventions [124]. Therefore, interventions involving a single component may be preferred as they are easier to design, implement and replicate, and oftentimes are successful in influencing a behaviour change [124].

### 4.1 Limitations of the reviewed studies

The quality of the studies included in our review varied; almost two‐thirds of the studies reviewed had a low risk of bias, while fewer than one‐tenth of all studies had a high risk of bias. While most studies reviewed had a low risk of bias, our assessment of a study's quality was limited for some studies due to a failure to report critical information related to study methodologies, such as study designs, or participant inclusion criteria, or to adequately describe outcome measures. Based on the small number of studies or interventions reviewed for HPT and DM, there may have been challenges in searching and including these conditions or related interventions. In addition, one in five of the total reviewed studies did not have clearly defined outcome measures. Therefore, future studies assessing adherence to treatment should use validated adherence measures, to report reliable findings or make strong conclusions.

More rigorous research in this field is critical, as is a replication of studies with positive findings in other settings. Furthermore, some of the studies described in this review were multifaceted, with some delivering multiple intervention components and others providing adherence support as a part of a broader package of services; this makes it impossible to discern the relative effect of each intervention component or identify which aspects are most impactful on adherence. Another limitation of these studies, and adherence research in general, remains with the challenge of accurately measuring medication adherence and in the variety of methodologies utilized [13].

The exclusion of data from studies conducted in North Africa may have led to the exclusion of potentially informative studies; however, NCDs are increasingly becoming the main cause of mortality in SSA, where the diseases were responsible for 37% of deaths in 2019 [20, 21, 22].

This review was limited to studies written in English only due to authors’ or reviewers’ challenges in reviewing studies written in other languages. Therefore, we may have potentially excluded relevant articles written in other languages besides English. Importantly, non‐English speaking countries based in SSA may provide different but useful knowledge or evidence on intervention strategies to improve adherence to treatment. Additionally, while we feel that the search terms or strings we used were able to remove irrelevant articles, it is possible that the search strings we used were not comprehensive enough.

## 5 CONCLUSIONS

Our study found substantial evidence of interventions to improve adherence among adults living with chronic conditions in SSA. There is more evidence that community‐ and home‐based, mhealth and adherence counselling interventions can improve adherence to chronic conditions. These tested and evaluated adherences enhancing interventions should increasingly be considered for routine implementation in health programmes. However, rigorous ongoing evaluation of the impact and performance of these interventions will be necessary. Multifaceted adherence intervention strategies that include reliable adherence measures such as drug exposure testing and socio‐economic support components such as cash vouchers provided to patients may be more effective than using a single‐component intervention strategy. Therefore, evidence gaps in adherence‐enhancing interventions need to be closed, including on cost‐effectiveness and long‐term effectiveness. Future research should seek to answer if existing intervention strategies can be successfully adapted for all chronic conditions assessed in this review. Our findings support testing more interventions and the need to develop a gold standard (or uniform measures) for adherence outcome ascertainment.

## COMPETING INTERESTS

AMJW reports grants or contracts from an investigator‐initiated grant from Gilead Sciences Global for the study of HIV Integrase resistance (Rosetta) and consulting fees from Gilead Sciences and ViiV Healthcare/GSK. Grants from charity/government institutions: Aidsfonds, NWO and Health Holland ICD‐ICK4HIVCure. Payment or honoraria for lectures, presentations or educational events from Virology Education and the Southern African HIV Clinicians Society. AMJW serves as a CEO for the European Society for Antiviral Research (ESAR), Member of the WHO Resnet, Chair of the IAS‐USA HIV drug resistance panel and as an Organizing Committee member for the international drug resistance workshop. WDFV received funding for the ADVANCE RCT through his institution from UNITAID, USAID and SAMRC, and received a study drug from ViiV Healthcare and Gilead Sciences. WDFV also reports funding for his unit from the Bill and Melinda Gates Foundation, National Institutes for Health, UNITAID, Foundation for Innovative New Diagnostics (FIND) and the Children's Investment Fund Foundation (CIFF), and received drug donations from Merck and J&J Sciences for investigator‐led clinical studies. The unit leads investigator‐led studies that receive financial support from Merck and ViiV and is involved in commercial drug studies for Merck. The unit performs evaluations of diagnostic devices for multiple biotech companies. WDFV also receives honoraria for educational talks and advisory board membership for Gilead, ViiV, Mylan, Merck, Adcock‐Ingram, Aspen, Abbott, Roche, J&J, Sanofi and Virology Education; participates on DSMB for NIH International; is currently an unpaid board member for Dira Sengwe and was an unpaid board member for SAHCS. None of the ADVANCE RCT funders were involved in the design, execution or analysis of this study. All other authors report no potential conflicts.

## AUTHORS’ CONTRIBUTIONS

SBG, STL‐E and JBFDW contributed to the conception and design of the study. SBG organized the database and performed the analysis. SBG wrote the first draft of the manuscript. SBG, JBFDW, WDFV, AMJW and STL‐E wrote sections of the manuscript. All authors contributed to the manuscript revision, read and approved the submitted version.

## FUNDING

This research was supported by the Consortium for Advanced Research Training in Africa (CARTA). CARTA is jointly led by the African Population and Health Research Center and the University of the Witwatersrand. SBG was funded by the Carnegie Corporation of New York (Grant No. G‐19‐57145), Sida (Grant No. 54100113), Uppsala Monitoring Center, Norwegian Agency for Development Cooperation (Norad) and by the Wellcome Trust [reference no. 107768/Z/15/Z] and the UK Foreign, Commonwealth & Development Office, with support from the Developing Excellence in Leadership, Training and Science in Africa (DELTAS Africa) programme.

WDFV and STL‐E are supported by the National Heart, Lung, and Blood Institute of the National Institutes of Health (Award Number UG3HL156388).

## DISCLAIMER

The statements and views made in this article are solely the responsibility of the authors and do not necessarily represent the official views of the National Institutes of Health.

## Supporting information

Supporting information

Supporting information

Supporting information

Supporting information

## Data Availability

The datasets used or analysed for the current study are available from the corresponding author on reasonable request.
