# Supplementary material for: Intervention strategies to improve adherence to treatment for selected chronic conditions in sub‐Saharan Africa: a systematic review
Source: J Int AIDS Soc. 2024 Jun 25;27(6):e26266. doi: 10.1002/jia2.26266 (PMC11197966; doi:10.1002/jia2.26266)
Supplement: Supplementary file 2 — Supporting information [file JIA2-27-e26266-s003.docx]

**Supplementary material 2: Search term strategy**

**PUBMED**

| 1 | "HIV Treatment" OR "Antiretroviral Therapy" OR "Antiretroviral Treatment" OR "ART treatment" OR "ART adherence" OR "ART Programs" OR "ART Programmes" OR "Mellitus Diabetes" OR "Hypertension" OR "Hypertension treatment" OR "Hypertension treatment adherence" OR “Stroke” OR "Chronic conditions" AND "Adherence" OR "Compliance" AND "Intervention" OR "Strategies" OR "Odds ratio" OR "risk ratio" OR "Evaluation" OR "Impact" OR "Effectiveness" OR "Outcome" AND "sub-Saharan Africa" OR "sub Saharan Africa" OR "sub-Saharan African" OR "sub Saharan African" OR "Sub-Saharan Africa" OR "Africa" OR "Angola" OR "Benin" OR "Botswana" OR "Burkina Faso" OR "Burundi" OR "Cabo Verde" OR "Cameroon" OR "Central African Republic" OR "Chad" OR "Comoros" OR "Democratic Republic of Congo" Or "Congo Republic" OR "Cote D'ivoire" OR "Equatorial Guinea" OR "Eritrea" OR "Eswatini" OR "Ethiopia" OR "Gabon" OR "Gambia" OR "Ghana" OR "Guinea" OR "Guinea-Bissau" OR "Kenya" OR "Lesotho" OR "Liberia" OR "Madagascar" OR "Madagascar" OR "Malawi" OR "Mali" OR "Mauritania" OR "Mauritius" OR "Mozambique" OR "Namibia" OR "Niger" OR "Nigeria" OR "Rwanda" OR "Sao Tome And Principe" OR "Senegal" OR "Seychelles" OR "Sierra Leone" OR "Somalia" OR "South Africa" OR "South Sudan" OR "Sudan" "Tanzania" OR "Togo" OR "Uganda" OR "Zambia" OR "Zimbabwe"[MeSH Terms] |
| --- | --- |
| 2 | "HIV Treatment"[Title/Abstract] OR "Antiretroviral Therapy"[Title/Abstract] OR "Antiretroviral Treatment"[Title/Abstract] OR "ART treatment"[Title/Abstract] OR "ART adherence"[Title/Abstract] OR "ART Programs"[Title/Abstract] OR "ART Programmes"[Title/Abstract] OR "Mellitus Diabetes"[Title/Abstract] OR "Hypertension"[Title/Abstract] OR "Hypertension treatment"[Title/Abstract] OR "Hypertension treatment adherence"[Title/Abstract] OR "Chronic conditions"[Title/Abstract] OR "Stroke"[Title/Abstract] AND "Adherence"[Title/Abstract] OR "Compliance"[Title/Abstract] AND "Intervention"[Title/Abstract] OR "Strategies"[Title/Abstract] OR "Odds ratio"[Title/Abstract] OR "risk ratio"[Title/Abstract] OR "Evaluation"[Title/Abstract] OR "Impact"[Title/Abstract] OR "Effectiveness"[Title/Abstract] OR "Outcome"[Title/Abstract] AND "sub-Saharan Africa"[Title/Abstract] OR "sub Saharan Africa"[Title/Abstract] OR "sub-Saharan African"[Title/Abstract] OR "sub Saharan African"[Title/Abstract] OR "Sub-Saharan Africa"[Title/Abstract] OR "Africa"[Title/Abstract] OR "Angola"[Title/Abstract] OR "Benin"[Title/Abstract] OR "Botswana"[Title/Abstract] OR "Burkina Faso"[Title/Abstract] OR "Burundi"[Title/Abstract] OR "Cabo Verde"[Title/Abstract] OR "Cameroon"[Title/Abstract] OR "Central African Republic"[Title/Abstract] OR "Chad"[Title/Abstract] OR "Comoros"[Title/Abstract] OR "Democratic Republic of Congo"[Title/Abstract] OR "Congo Republic"[Title/Abstract] OR "Cote D'ivoire"[Title/Abstract] OR "Equatorial Guinea"[Title/Abstract] OR "Eritrea"[Title/Abstract] OR "Eswatini"[Title/Abstract] OR "Ethiopia"[Title/Abstract] OR "Gabon"[Title/Abstract] OR "Gambia"[Title/Abstract] OR "Ghana"[Title/Abstract] OR "Guinea"[Title/Abstract] OR "Guinea-Bissau"[Title/Abstract] OR "Kenya"[Title/Abstract] OR "Lesotho"[Title/Abstract] OR "Liberia"[Title/Abstract] OR "Madagascar"[Title/Abstract] OR "Malawi"[Title/Abstract] OR "Mali"[Title/Abstract] OR "Mauritania"[Title/Abstract] OR "Mauritius"[Title/Abstract] OR "Mozambique"[Title/Abstract] OR "Namibia"[Title/Abstract] OR "Niger"[Title/Abstract] OR "Nigeria"[Title/Abstract] OR "Rwanda"[Title/Abstract] OR "Sao Tome[Title/Abstract] OR Principe"[Title/Abstract] OR "Senegal"[Title/Abstract] OR "Seychelles"[Title/Abstract] OR "Sierra Leone"[Title/Abstract] OR "Somalia"[Title/Abstract] OR "South Africa"[Title/Abstract] OR "South Sudan"[Title/Abstract] OR "Sudan" "Tanzania"[Title/Abstract] OR "Togo"[Title/Abstract] OR "Uganda"[Title/Abstract] OR "Zambia"[Title/Abstract] OR "Zimbabwe"[Title/Abstract] |
| 3 | (English[Language]) |
| 4 | (("2000/01/01"[Date - Publication] : "2022/11/01"[Date - Publication])) |
| 5 | 1 OR 2 |

**WEB OF SCIENCE**

| 1 | ALL=(‘HIV Treatment’ OR ‘Antiretroviral Therapy’ OR ‘Antiretroviral Treatment’ OR ‘ART treatment’ OR ‘ART adherence’ OR ‘ART Programs’ OR ‘ART Programmes’ OR ‘Mellitus Diabetes’ OR ‘Mellitus Diabetes treatment’ OR ‘Mellitus Diabetes adherence’ OR ‘Hypertension’ OR ‘Hypertension treatment’ OR ‘Hypertension treatment adherence’ OR ‘Chronic conditions’ AND ‘Adherence’ OR ‘Compliance’ ) |
| --- | --- |
| 2 | Publication Years: **2022** or **2021** or **2020** or **2019** or **2018** or **2017** or **2016** or **2015** or **2014** or **2013** or **2012** or **2011** or **2010** or **2009** or **2008** or **2007** or **2006** or **2005** or **2004** or **2003** or **2002** or **2001** or **2000** |
| 3 | Languages: English. |
| 4 | Citation Topics Meso: 1.26 Diabetes or 1.66 Hiv or 1.55 Urology & Nephrology - General or 1.37 Cardiology - General or 1.44 Nutrition & Dietetics or 1.104 Virology - General or 1.105 Strokes or 1.156 Healthcare Policy or 1.155 Medical Ethics or 1.112 Palliative Care or 4.13 Telecommunications. |
| 5 | Research Areas: Cardiovascular System Cardiology or General Internal Medicine or Infectious Diseases or Immunology or Health Care Sciences Services or Research Experimental Medicine or Nutrition Dietetics or Science Technology Other Topics or Respiratory System or Virology or Nursing or Biomedical Social Sciences or Social Sciences Other Topics or Religion or Government Law or Family Studies or Education Educational Research or Telecommunications or Ethnic Studies or Social Issues or Communication. |
| 6 | Countries/Regions: SOUTH AFRICA or ETHIOPIA or UGANDA or NIGERIA or KENYA or TANZANIA or GHANA or ZIMBABWE or ZAMBIA or MALAWI or CAMEROON or MOZAMBIQUE or BOTSWANA or DEM REP CONGO or RWANDA or SUDAN or COTE IVOIRE or BURKINA FASO or LESOTHO or NAMIBIA or SENEGAL or ESWATINI or BENIN or REP CONGO or SOMALIA or TOGO or BURUNDI or MALI or GUINEA or GABON or LIBERIA or MAURITIUS or ANGOLA or GUINEA BISSAU or NIGER or SIERRA LEONE or ERITREA or GAMBIA or SEYCHELLES or SOUTH SUDAN or CAPE VERDE or CHAD or COTE D IVOIRE or MADAGASCAR. |
| 7 | Open Access:Gold or Gold-Hybrid or All Open Access or Free to Read or Green Published or Green Accepted or Green Submitted |
| 8 | Web of Science Categories: Infectious Diseases or Medicine General Internal or Immunology or Multidisciplinary Sciences or Health Care Sciences Services or Public Environmental Occupational Health or Cardiac Cardiovascular Systems or Virology or Peripheral Vascular Disease or Social Sciences Biomedical or Health Policy Services or Microbiology or Medicine Research Experimental or Nutrition Dietetics or Pharmacology Pharmacy or Respiratory System or Primary Health Care or Psychology Multidisciplinary or Medical Informatics or Nursing or Social Sciences Interdisciplinary or Tropical Medicine or Family Studies or Biology or Social Work or Clinical Neurology or Computer Science Information Systems or Economics or Education Scientific Disciplines or Ethics or Green Sustainable Science Technology or Medical Ethics or Psychiatry or Radiology Nuclear Medicine Medical Imaging or Reproductive Biology or Biochemical Research Methods or Education Educational Research or Food Science Technology or Physiology or Political Science or Psychology Applied or Psychology Clinical or Psychology Social or Public Administration or Telecommunications or Toxicology. |

**CINAHL**

| 1 | "HIV Treatment" OR “Antiretroviral Therapy” OR “Antiretroviral Treatment” OR “ART treatment” OR “ART adherence” OR “ART Programs” OR “ART Programmes” OR “Mellitus Diabetes” OR “Hypertension” OR “Hypertension treatment” OR “Hypertension treatment adherence” OR “Chronic conditions” OR “Stroke” AND “Adherence” OR “Compliance” AND “Intervention” OR “Strategies” OR “Odds ratio” OR “risk ratio” OR “Evaluation” OR “Impact” OR “Effectiveness” OR “Outcome” AND “sub-Saharan Africa” OR “sub Saharan Africa” OR “sub-Saharan African” OR “sub Saharan African” OR “Sub-Saharan Africa” OR “Africa” OR “Angola” OR “Benin” OR “Botswana” OR “Burkina Faso” OR “Burundi” OR “Cabo Verde” OR “Cameroon” OR “Central African Republic” OR “Chad” OR “Comoros” OR “Democratic Republic of Congo” Or “Congo Republic” OR “Cote D'ivoire” OR “Equatorial Guinea” OR “Eritrea” OR “Eswatini” OR “Ethiopia” OR “Gabon” OR “Gambia” OR “Ghana” OR “Guinea” OR “Guinea-Bissau” OR “Kenya” OR “Lesotho” OR “Liberia” OR “Madagascar” OR “Madagascar” OR “Malawi” OR “Mali” OR “Mauritania” OR “Mauritius” OR “Mozambique” OR “Namibia” OR “Niger” OR “Nigeria” OR “Rwanda” OR “Sao Tome And Principe” OR “Senegal” OR “Seychelles” OR “Sierra Leone” OR “Somalia” OR “South Africa” OR “South Sudan” OR “Sudan” “Tanzania” OR “Togo” OR “Uganda” OR “Zambia” OR “Zimbabwe” |
| --- | --- |
| 2 | **Limiters** - Published Date: 20000101-20221131; English Language; Human; Age Groups: All Adult; Geographic Subset: Africa; Language: English |
| 3 | **Expanders** - Also search within the full text of the articles; Apply equivalent subjects |
| 4 | **Search modes** - Find all my search terms |
| 5 | **Expanders** - Apply equivalent subjects |
|  | **Narrow by SubjectGeographic** - Africa |
| 6 | **Search modes** - Boolean/Phrase |

**SCOPUS**

| 1 | TITLE-ABS-KEY ( "HIV Treatment"  OR  "Antiretroviral Therapy"  OR  "Antiretroviral Treatment"  OR  "ART treatment"  OR  "ART adherence"  OR  "ART Programs"  OR  "ART Programmes"  OR  "Mellitus Diabetes"  OR  "Hypertension"  OR  "Hypertension treatment"  OR  "Hypertension treatment adherence"  OR  "Chronic conditions"  OR  "Stroke"  AND  "Adherence"  OR  "Compliance"  AND  "Intervention"  OR  "Strategies"  OR  "Odds ratio"  OR  "risk ratio"  OR  "Evaluation"  OR  "Impact"  OR  "Effectiveness"  OR  "Outcome"  AND  "sub-Saharan Africa"  OR  "sub Saharan Africa"  OR  "sub-Saharan African"  OR  "sub Saharan African"  OR  "Sub-Saharan Africa"  OR  "Africa"  OR  "Angola"  OR  "Benin"  OR  "Botswana"  OR  "Burkina Faso"  OR  "Burundi"  OR  "Cabo Verde"  OR  "Cameroon"  OR  "Central African Republic"  OR  "Chad"  OR  "Comoros"  OR  "Democratic Republic of Congo"  OR  "Congo Republic"  OR  "Cote D'ivoire"  OR  "Equatorial Guinea"  OR  "Eritrea"  OR  "Eswatini"  OR  "Ethiopia"  OR  "Gabon"  OR  "Gambia"  OR  "Ghana"  OR  "Guinea"  OR  "Guinea-Bissau"  OR  "Kenya"  OR  "Lesotho"  OR  "Liberia"  OR  "Madagascar"  OR  "Madagascar"  OR  "Malawi"  OR  "Mali"  OR  "Mauritania"  OR  "Mauritius"  OR  "Mozambique"  OR  "Namibia"  OR  "Niger"  OR  "Nigeria"  OR  "Rwanda"  OR  "Sao Tome And Principe"  OR  "Senegal"  OR  "Seychelles"  OR  "Sierra Leone"  OR  "Somalia"  OR  "South Africa"  OR  "South Sudan"  OR  "Sudan"  "Tanzania"  OR  "Togo"  OR  "Uganda"  OR  "Zambia"  OR  "Zimbabwe" ) |
| --- | --- |
| **2** | ( LIMIT-TO ( LANGUAGE ,  "English" ) |
| **3** | AND  (  LIMIT-TO ( OA ,  "all" )   OR  LIMIT-TO ( OA ,  "publisherfullgold" )   OR  LIMIT-TO ( OA ,  "publisherhybridgold" )   OR  LIMIT-TO ( OA ,  "publisherfree2read" )   OR  LIMIT-TO ( OA ,  "repository" ) ) |
| **4** | ( LIMIT-TO ( PUBYEAR ,  2022 )  OR  LIMIT-TO ( PUBYEAR ,  2021 )  OR  LIMIT-TO ( PUBYEAR ,  2020 )  OR  LIMIT-TO ( PUBYEAR ,  2019 )  OR  LIMIT-TO ( PUBYEAR ,  2018 )  OR  LIMIT-TO ( PUBYEAR ,  2017 )  OR  LIMIT-TO ( PUBYEAR ,  2016 )  OR  LIMIT-TO ( PUBYEAR ,  2015 )  OR  LIMIT-TO ( PUBYEAR ,  2014 )  OR  LIMIT-TO ( PUBYEAR ,  2013 )  OR  LIMIT-TO ( PUBYEAR ,  2012 )  OR  LIMIT-TO ( PUBYEAR ,  2011 )  OR  LIMIT-TO ( PUBYEAR ,  2010 )  OR  LIMIT-TO ( PUBYEAR ,  2009 )  OR  LIMIT-TO ( PUBYEAR ,  2008 )  OR  LIMIT-TO ( PUBYEAR ,  2007 )  OR  LIMIT-TO ( PUBYEAR ,  2006 )  OR  LIMIT-TO ( PUBYEAR ,  2005 )  OR  LIMIT-TO ( PUBYEAR ,  2004 )  OR  LIMIT-TO ( PUBYEAR ,  2003 )  OR  LIMIT-TO ( PUBYEAR ,  2002 ) ) |
| **5** | ( LIMIT-TO ( SUBJAREA ,  "BIOC" )  OR  LIMIT-TO ( SUBJAREA ,  "DECI" )  OR  LIMIT-TO ( SUBJAREA ,  "HEAL" )  OR  LIMIT-TO ( SUBJAREA ,  "IMMU" )  OR  LIMIT-TO ( SUBJAREA ,  "MEDI" )  OR  LIMIT-TO ( SUBJAREA ,  "PSYC" )  OR  LIMIT-TO ( SUBJAREA ,  "MULT" )  OR  LIMIT-TO ( SUBJAREA ,  "SOCI" )  OR  LIMIT-TO ( SUBJAREA ,  "PHAR" )  OR  LIMIT-TO ( SUBJAREA ,  "NURS" )  OR  LIMIT-TO ( SUBJAREA ,  "ENVI" ) ) |
| **6** | ( LIMIT-TO ( AFFILCOUNTRY ,  "Uganda" )  OR  LIMIT-TO ( AFFILCOUNTRY ,  "South Africa" )  OR  LIMIT-TO ( AFFILCOUNTRY ,  "Tanzania" )  OR  LIMIT-TO ( AFFILCOUNTRY ,  "Zambia" )  OR  LIMIT-TO ( AFFILCOUNTRY ,  "Zimbabwe" )  OR  LIMIT-TO ( AFFILCOUNTRY ,  "Kenya" )  OR  LIMIT-TO ( AFFILCOUNTRY ,  "Malawi" )  OR  LIMIT-TO ( AFFILCOUNTRY ,  "Nigeria" )  OR  LIMIT-TO ( AFFILCOUNTRY ,  "Togo" )  OR  LIMIT-TO ( AFFILCOUNTRY ,  "Cameroon" )  OR  LIMIT-TO ( AFFILCOUNTRY ,  "Cote d'Ivoire" )  OR  LIMIT-TO ( AFFILCOUNTRY ,  "Mozambique" )  OR  LIMIT-TO ( AFFILCOUNTRY ,  "Ethiopia" )  OR  LIMIT-TO ( AFFILCOUNTRY ,  "Burkina Faso" )  OR  LIMIT-TO ( AFFILCOUNTRY ,  "Ghana" )  OR  LIMIT-TO ( AFFILCOUNTRY ,  "Senegal" )  OR  LIMIT-TO ( AFFILCOUNTRY ,  "Benin" )  OR  LIMIT-TO ( AFFILCOUNTRY ,  "Botswana" )  OR  LIMIT-TO ( AFFILCOUNTRY ,  "Democratic Republic Congo" )  OR  LIMIT-TO ( AFFILCOUNTRY ,  "Rwanda" )  OR  LIMIT-TO ( AFFILCOUNTRY ,  "Swaziland" )  OR  LIMIT-TO ( AFFILCOUNTRY ,  "Congo" )  OR  LIMIT-TO ( AFFILCOUNTRY ,  "Gabon" )  OR  LIMIT-TO ( AFFILCOUNTRY ,  "Guinea" )  OR  LIMIT-TO ( AFFILCOUNTRY ,  "Lesotho" )  OR  LIMIT-TO ( AFFILCOUNTRY ,  "Mauritania" )  OR  LIMIT-TO ( AFFILCOUNTRY ,  "Niger" ) ) |

**GOOGLE SCHOLAR**

|  | **With all of the words** | **With at least one of the words** |
| --- | --- | --- |
| **1** | **allintitle: Antiretroviral Therapy AND Adherence OR Intervention** | "HIV Treatment" OR “Antiretroviral Therapy” OR “Antiretroviral Treatment” OR “ART treatment” OR “ART adherence” OR “ART Programs” OR “ART Programmes” OR “Mellitus Diabetes” OR “Hypertension” OR “Hypertension treatment” OR “Hypertension treatment adherence” OR “Chronic conditions” OR “Stroke” AND “Adherence” OR “Compliance” AND “Intervention” OR “Strategies” OR “Odds ratio” OR “risk ratio” OR “Evaluation” OR “Impact” OR “Effectiveness” OR “Outcome” AND “sub-Saharan Africa” OR “sub Saharan Africa” OR “sub-Saharan African” OR “sub Saharan African” OR “Sub-Saharan Africa” OR “Africa” OR “Angola” OR “Benin” OR “Botswana” OR “Burkina Faso” OR “Burundi” OR “Cabo Verde” OR “Cameroon” OR “Central African Republic” OR “Chad” OR “Comoros” OR “Democratic Republic of Congo” Or “Congo Republic” OR “Cote D'ivoire” OR “Equatorial Guinea” OR “Eritrea” OR “Eswatini” OR “Ethiopia” OR “Gabon” OR “Gambia” OR “Ghana” OR “Guinea” OR “Guinea-Bissau” OR “Kenya” OR “Lesotho” OR “Liberia” OR “Madagascar” OR “Madagascar” OR “Malawi” OR “Mali” OR “Mauritania” OR “Mauritius” OR “Mozambique” OR “Namibia” OR “Niger” OR “Nigeria” OR “Rwanda” OR “Sao Tome And Principe” OR “Senegal” OR “Seychelles” OR “Sierra Leone” OR “Somalia” OR “South Africa” OR “South Sudan” OR “Sudan” “Tanzania” OR “Togo” OR “Uganda” OR “Zambia” OR “Zimbabwe” |
| **2** | **allintitle: Antiretroviral Treatment AND Adherence OR Intervention** | "HIV Treatment" OR “Antiretroviral Therapy” OR “Antiretroviral Treatment” OR “ART treatment” OR “ART adherence” OR “ART Programs” OR “ART Programmes” OR “Mellitus Diabetes” OR “Hypertension” OR “Hypertension treatment” OR “Hypertension treatment adherence” OR “Chronic conditions” OR “Stroke” AND “Adherence” OR “Compliance” AND “Intervention” OR “Strategies” OR “Odds ratio” OR “risk ratio” OR “Evaluation” OR “Impact” OR “Effectiveness” OR “Outcome” AND “sub-Saharan Africa” OR “sub Saharan Africa” OR “sub-Saharan African” OR “sub Saharan African” OR “Sub-Saharan Africa” OR “Africa” OR “Angola” OR “Benin” OR “Botswana” OR “Burkina Faso” OR “Burundi” OR “Cabo Verde” OR “Cameroon” OR “Central African Republic” OR “Chad” OR “Comoros” OR “Democratic Republic of Congo” Or “Congo Republic” OR “Cote D'ivoire” OR “Equatorial Guinea” OR “Eritrea” OR “Eswatini” OR “Ethiopia” OR “Gabon” OR “Gambia” OR “Ghana” OR “Guinea” OR “Guinea-Bissau” OR “Kenya” OR “Lesotho” OR “Liberia” OR “Madagascar” OR “Madagascar” OR “Malawi” OR “Mali” OR “Mauritania” OR “Mauritius” OR “Mozambique” OR “Namibia” OR “Niger” OR “Nigeria” OR “Rwanda” OR “Sao Tome And Principe” OR “Senegal” OR “Seychelles” OR “Sierra Leone” OR “Somalia” OR “South Africa” OR “South Sudan” OR “Sudan” “Tanzania” OR “Togo” OR “Uganda” OR “Zambia” OR “Zimbabwe” |
| **3** | **allintitle: ART AND Adherence OR Intervention** | ""HIV Treatment" OR “Antiretroviral Therapy” OR “Antiretroviral Treatment” OR “ART treatment” OR “ART adherence” OR “ART Programs” OR “ART Programmes” OR “Mellitus Diabetes” OR “Hypertension” OR “Hypertension treatment” OR “Hypertension treatment adherence” OR “Chronic conditions” OR “Stroke” AND “Adherence” OR “Compliance” AND “Intervention” OR “Strategies” OR “Odds ratio” OR “risk ratio” OR “Evaluation” OR “Impact” OR “Effectiveness” OR “Outcome” AND “sub-Saharan Africa” OR “sub Saharan Africa” OR “sub-Saharan African” OR “sub Saharan African” OR “Sub-Saharan Africa” OR “Africa” OR “Angola” OR “Benin” OR “Botswana” OR “Burkina Faso” OR “Burundi” OR “Cabo Verde” OR “Cameroon” OR “Central African Republic” OR “Chad” OR “Comoros” OR “Democratic Republic of Congo” Or “Congo Republic” OR “Cote D'ivoire” OR “Equatorial Guinea” OR “Eritrea” OR “Eswatini” OR “Ethiopia” OR “Gabon” OR “Gambia” OR “Ghana” OR “Guinea” OR “Guinea-Bissau” OR “Kenya” OR “Lesotho” OR “Liberia” OR “Madagascar” OR “Madagascar” OR “Malawi” OR “Mali” OR “Mauritania” OR “Mauritius” OR “Mozambique” OR “Namibia” OR “Niger” OR “Nigeria” OR “Rwanda” OR “Sao Tome And Principe” OR “Senegal” OR “Seychelles” OR “Sierra Leone” OR “Somalia” OR “South Africa” OR “South Sudan” OR “Sudan” “Tanzania” OR “Togo” OR “Uganda” OR “Zambia” OR “Zimbabwe” |
| **4** | **allintitle: Mellitus Diabetes AND Adherence OR Intervention** | "HIV Treatment" OR “Antiretroviral Therapy” OR “Antiretroviral Treatment” OR “ART treatment” OR “ART adherence” OR “ART Programs” OR “ART Programmes” OR “Mellitus Diabetes” OR “Hypertension” OR “Hypertension treatment” OR “Hypertension treatment adherence” OR “Chronic conditions” OR “Stroke” AND “Adherence” OR “Compliance” AND “Intervention” OR “Strategies” OR “Odds ratio” OR “risk ratio” OR “Evaluation” OR “Impact” OR “Effectiveness” OR “Outcome” AND “sub-Saharan Africa” OR “sub Saharan Africa” OR “sub-Saharan African” OR “sub Saharan African” OR “Sub-Saharan Africa” OR “Africa” OR “Angola” OR “Benin” OR “Botswana” OR “Burkina Faso” OR “Burundi” OR “Cabo Verde” OR “Cameroon” OR “Central African Republic” OR “Chad” OR “Comoros” OR “Democratic Republic of Congo” Or “Congo Republic” OR “Cote D'ivoire” OR “Equatorial Guinea” OR “Eritrea” OR “Eswatini” OR “Ethiopia” OR “Gabon” OR “Gambia” OR “Ghana” OR “Guinea” OR “Guinea-Bissau” OR “Kenya” OR “Lesotho” OR “Liberia” OR “Madagascar” OR “Madagascar” OR “Malawi” OR “Mali” OR “Mauritania” OR “Mauritius” OR “Mozambique” OR “Namibia” OR “Niger” OR “Nigeria” OR “Rwanda” OR “Sao Tome And Principe” OR “Senegal” OR “Seychelles” OR “Sierra Leone” OR “Somalia” OR “South Africa” OR “South Sudan” OR “Sudan” “Tanzania” OR “Togo” OR “Uganda” OR “Zambia” OR “Zimbabwe” |
| **5** | **allintitle: Hypertension AND Adherence OR Intervention** | "HIV Treatment" OR “Antiretroviral Therapy” OR “Antiretroviral Treatment” OR “ART treatment” OR “ART adherence” OR “ART Programs” OR “ART Programmes” OR “Mellitus Diabetes” OR “Hypertension” OR “Hypertension treatment” OR “Hypertension treatment adherence” OR “Chronic conditions” OR “Stroke” AND “Adherence” OR “Compliance” AND “Intervention” OR “Strategies” OR “Odds ratio” OR “risk ratio” OR “Evaluation” OR “Impact” OR “Effectiveness” OR “Outcome” AND “sub-Saharan Africa” OR “sub Saharan Africa” OR “sub-Saharan African” OR “sub Saharan African” OR “Sub-Saharan Africa” OR “Africa” OR “Angola” OR “Benin” OR “Botswana” OR “Burkina Faso” OR “Burundi” OR “Cabo Verde” OR “Cameroon” OR “Central African Republic” OR “Chad” OR “Comoros” OR “Democratic Republic of Congo” Or “Congo Republic” OR “Cote D'ivoire” OR “Equatorial Guinea” OR “Eritrea” OR “Eswatini” OR “Ethiopia” OR “Gabon” OR “Gambia” OR “Ghana” OR “Guinea” OR “Guinea-Bissau” OR “Kenya” OR “Lesotho” OR “Liberia” OR “Madagascar” OR “Madagascar” OR “Malawi” OR “Mali” OR “Mauritania” OR “Mauritius” OR “Mozambique” OR “Namibia” OR “Niger” OR “Nigeria” OR “Rwanda” OR “Sao Tome And Principe” OR “Senegal” OR “Seychelles” OR “Sierra Leone” OR “Somalia” OR “South Africa” OR “South Sudan” OR “Sudan” “Tanzania” OR “Togo” OR “Uganda” OR “Zambia” OR “Zimbabwe” |
| **6** | **allintitle: Hypertension treatment AND Adherence OR Intervention** | "HIV Treatment" OR “Antiretroviral Therapy” OR "HIV Treatment" OR “Antiretroviral Therapy” OR “Antiretroviral Treatment” OR “ART treatment” OR “ART adherence” OR “ART Programs” OR “ART Programmes” OR “Mellitus Diabetes” OR “Hypertension” OR “Hypertension treatment” OR “Hypertension treatment adherence” OR “Chronic conditions” OR “Stroke” AND “Adherence” OR “Compliance” AND “Intervention” OR “Strategies” OR “Odds ratio” OR “risk ratio” OR “Evaluation” OR “Impact” OR “Effectiveness” OR “Outcome” AND “sub-Saharan Africa” OR “sub Saharan Africa” OR “sub-Saharan African” OR “sub Saharan African” OR “Sub-Saharan Africa” OR “Africa” OR “Angola” OR “Benin” OR “Botswana” OR “Burkina Faso” OR “Burundi” OR “Cabo Verde” OR “Cameroon” OR “Central African Republic” OR “Chad” OR “Comoros” OR “Democratic Republic of Congo” Or “Congo Republic” OR “Cote D'ivoire” OR “Equatorial Guinea” OR “Eritrea” OR “Eswatini” OR “Ethiopia” OR “Gabon” OR “Gambia” OR “Ghana” OR “Guinea” OR “Guinea-Bissau” OR “Kenya” OR “Lesotho” OR “Liberia” OR “Madagascar” OR “Madagascar” OR “Malawi” OR “Mali” OR “Mauritania” OR “Mauritius” OR “Mozambique” OR “Namibia” OR “Niger” OR “Nigeria” OR “Rwanda” OR “Sao Tome And Principe” OR “Senegal” OR “Seychelles” OR “Sierra Leone” OR “Somalia” OR “South Africa” OR “South Sudan” OR “Sudan” “Tanzania” OR “Togo” OR “Uganda” OR “Zambia” OR “Zimbabwe” |
| **7** | **allintitle: Hypertension therapy AND Adherence OR Intervention** | "HIV Treatment" OR “Antiretroviral Therapy” OR “Antiretroviral Treatment” OR “ART treatment” OR “ART adherence” OR “ART Programs” OR “ART Programmes” OR “Mellitus Diabetes” OR “Hypertension” OR “Hypertension treatment” OR “Hypertension treatment adherence” OR “Chronic conditions” OR “Stroke” AND “Adherence” OR “Compliance” AND “Intervention” OR “Strategies” OR “Odds ratio” OR “risk ratio” OR “Evaluation” OR “Impact” OR “Effectiveness” OR “Outcome” AND “sub-Saharan Africa” OR “sub Saharan Africa” OR “sub-Saharan African” OR “sub Saharan African” OR “Sub-Saharan Africa” OR “Africa” OR “Angola” OR “Benin” OR “Botswana” OR “Burkina Faso” OR “Burundi” OR “Cabo Verde” OR “Cameroon” OR “Central African Republic” OR “Chad” OR “Comoros” OR “Democratic Republic of Congo” Or “Congo Republic” OR “Cote D'ivoire” OR “Equatorial Guinea” OR “Eritrea” OR “Eswatini” OR “Ethiopia” OR “Gabon” OR “Gambia” OR “Ghana” OR “Guinea” OR “Guinea-Bissau” OR “Kenya” OR “Lesotho” OR “Liberia” OR “Madagascar” OR “Madagascar” OR “Malawi” OR “Mali” OR “Mauritania” OR “Mauritius” OR “Mozambique” OR “Namibia” OR “Niger” OR “Nigeria” OR “Rwanda” OR “Sao Tome And Principe” OR “Senegal” OR “Seychelles” OR “Sierra Leone” OR “Somalia” OR “South Africa” OR “South Sudan” OR “Sudan” “Tanzania” OR “Togo” OR “Uganda” OR “Zambia” OR “Zimbabwe” |
| **8** | **allintitle: Chronic conditions AND Adherence OR Intervention** | "HIV Treatment" OR “Antiretroviral Therapy” OR “Antiretroviral Treatment” OR “ART treatment” OR “ART adherence” OR “ART Programs” OR “ART Programmes” OR “Mellitus Diabetes” OR “Hypertension” OR “Hypertension treatment” OR “Hypertension treatment adherence” OR “Chronic conditions” OR “Stroke” AND “Adherence” OR “Compliance” AND “Intervention” OR “Strategies” OR “Odds ratio” OR “risk ratio” OR “Evaluation” OR “Impact” OR “Effectiveness” OR “Outcome” AND “sub-Saharan Africa” OR “sub Saharan Africa” OR “sub-Saharan African” OR “sub Saharan African” OR “Sub-Saharan Africa” OR “Africa” OR “Angola” OR “Benin” OR “Botswana” OR “Burkina Faso” OR “Burundi” OR “Cabo Verde” OR “Cameroon” OR “Central African Republic” OR “Chad” OR “Comoros” OR “Democratic Republic of Congo” Or “Congo Republic” OR “Cote D'ivoire” OR “Equatorial Guinea” OR “Eritrea” OR “Eswatini” OR “Ethiopia” OR “Gabon” OR “Gambia” OR “Ghana” OR “Guinea” OR “Guinea-Bissau” OR “Kenya” OR “Lesotho” OR “Liberia” OR “Madagascar” OR “Madagascar” OR “Malawi” OR “Mali” OR “Mauritania” OR “Mauritius” OR “Mozambique” OR “Namibia” OR “Niger” OR “Nigeria” OR “Rwanda” OR “Sao Tome And Principe” OR “Senegal” OR “Seychelles” OR “Sierra Leone” OR “Somalia” OR “South Africa” OR “South Sudan” OR “Sudan” “Tanzania” OR “Togo” OR “Uganda” OR “Zambia” OR “Zimbabwe” |
| **9** | **Language** | English |
| **10** | **Custom range** | 2000-2022 |
