# Supplementary material for: Intervention strategies to improve adherence to treatment for selected chronic conditions in sub‐Saharan Africa: a systematic review
Source: J Int AIDS Soc. 2024 Jun 25;27(6):e26266. doi: 10.1002/jia2.26266 (PMC11197966; doi:10.1002/jia2.26266)
Supplement: Supplementary file 3 — Supporting information [file JIA2-27-e26266-s001.docx]

**Supplementary material 3: Applied QUIPS tool and Risk of Bias**

| **Domain** | **Items for consideration** | **Rating** |
| --- | --- | --- |
| Study participation | Adequate description of period + place of recruitment  Adequate description of in/exclusion criteria  Adequate description of number + characteristics of study participants | **High risk:** Significant amount of eligible patients not included in the study (e.g., experimental design)  **Low risk:** almost all eligible patients included or random inclusion |
| Intervention described | Adequate description of how adherence intervention was formulated, defined, or described. | **High risk**: no clear description for the medication adherence intervention  **Low risk:** A clear description of the intervention and how it was measured, including the limitations |
| Outcome measurement | Clear definition of the study outcomes.  Method of measurement described | **High risk:** flexible definition including interruptions or depending on time of data collection with different time points.  **Low risk:** strict medication adherence definition with clear defined outcomes and related measurements |
| Sample and sample size | Sample has been described and where applicable, Sufficient sample size and/or rationale on how sample size was calculated. | **High risk:** no description of the sample and sample size reported, and no scientific justification for small or unmatching sample size  **Low risk:** Sample has been described and where applicable sample size has been reported and sample size calculations provided |
| **Overall (matched with EPHPP scores)** |  | **High risk:** If 2 or more high risks  **Moderate risk:** if 1 high risk and / or 2 or more unclear  **Low risk:** If maximum 1 unclear and no high risks |
