# Supplementary material for: Intervention strategies to improve adherence to treatment for selected chronic conditions in sub‐Saharan Africa: a systematic review
Source: J Int AIDS Soc. 2024 Jun 25;27(6):e26266. doi: 10.1002/jia2.26266 (PMC11197966; doi:10.1002/jia2.26266)
Supplement: Supplementary file 4 — Supporting information [file JIA2-27-e26266-s002.docx]

**Supplementary material 4: Characteristics of included studies**

| **Study title** | **First author, publication year** | **Type** | **Study duration** | **Country** | **Population** | **Setting** | **Study design** | **Sample size** | **Intervention description** | **Details on age (median, mean, range)** | **Sex** | **Condition (HIV, HTN, DM)** | **Outcome measures** | **Main results** | **Results summary of effective** |
| --- | --- | --- | --- | --- | --- | --- | --- | --- | --- | --- | --- | --- | --- | --- | --- |
| A peer adherence support intervention to improve the antiretroviral treatment outcomes of HIV patients in South Africa: the moderating role of family dynamics | Wouters et al., 2014 | Journal article | 2007-2008 | Free State Province, South Africa | HIV positive patients who are on ART | Public-sector ART programme of the Free State Province of South Africa | Cross-sectional secondary statistical analysis of post-trial data | 340 | Peer adherence support intervention | 37.0 (SD±9.1) | Female (77.4%) | HIV | Treatment outcomes were assessed using the patients’ CD4 cell counts, recorded closest to the date of the interview and extracted from the patient's files and electronic records | No significant overall differences in CD4 cell count between the intervention group accessing additional peer adherence support and the control group receiving standard care. When controlling for the potential moderating role of family dynamics, the outcomes showed a significant interaction effect between the adherence intervention and the level of family functioning about treatment outcomes. Multi-group analysis demonstrates that peer adherence support has a positive effect on immunological restoration in well-functioning families, while having a negative effect in dysfunctional families | Non-effective adherence strategy |
| A randomized control trial of a peer adherence and nutritional support program for public sector antiretroviral patients | Booysen et al., 2016 | Journal article | Not specified | Free State Province, South Africa | HIV positive patients who are on ART | Public health care setting in South Africa | Prospective cohort study and experimental study | 653 | Peer adherence and nutritional support | 37 (IQR 31-43) | Female (76.8%) | HIV | The impact of these peer adherence and nutritional support interventions on self-reported adherence, timeliness of clinic and hospital visits, and immunologic response to antiretroviral treatment | Peer adherence and nutritional support improved the timeliness of adults´ clinic and hospital visits for routine follow-up while on antiretroviral treatment. Peer adherence support impacted positively on immunologic response to antiretroviral treatment | Effective adherence strategy |
| A randomized controlled trial of real-time electronic adherence monitoring with text message dosing reminders in people starting first-line antiretroviral therapy | Orrell et al., 2015 | Journal article | 2012-2014 | Gugulethu, Cape Town | HIV positive patients on first-line ART | Large public sector urban ART outpatient clinic | Randomized controlled trial in ART-naive individuals | 230 | Simple text message that would remind patients to take their tablets, but not disclose their HIV status to others at home or in the community | 34.5 (SD±9.1) | Female (65.2%) | HIV | The primary outcome was adherence execution as measured by the electronic adherence monitoring device (EAMD). Adherence execution was calculated by the number of days the container was opened over the number of days in the period in care (for those who completed the study, transferred out or who died); and for the period from randomization to calculated week 48 for those LTFU. | Median adherence was 82.1% (interquartile range, 56.6%–94.6%) in the intervention arm, compared with 80.4% (interquartile range, 52.8%–93.8%) for SoC [adjusted odds ratio for adherence 1.08; 95% confidence interval (CI): 0.77 to 1.52]. Suppressed HIV RNA (<40 copies/mL) occurred in 80 (69.6%) of control and 75 (65.2%) of intervention (adjusted odds ratio for virological failure in intervention arm 0.77; 95% CI: 0.42 to 1.40). In the intervention arm, the count of TIs of >72 hours was reduced (adjusted incident rate ratio, 0.84; 95% CI: 0.75 to 0.94) | Effective adherence strategy |
| Adherence clubs and decentralized medication delivery to support patient retention and sustained viral suppression in care: Results from a cluster-randomized evaluation of differentiated ART delivery models in South Africa | Fox et al., 2019 | Journal article | 2015-2016 | Gauteng, North West, Limpopo, and KwaZulu Natal-South Africa | HIV patients who are on ART | The study was conducted in 24 health facilities (12 intervention, 12 control sites) in Gauteng, KwaZulu Natal, Limpopo, and North West provinces | Unblinded cluster-randomized evaluation design | 569 | Adherence Clubs (ACs) and Decentralized Medication Delivery (DMD): ACs comprise clinically stable ART patients who meet at facilities or community locations in groups of up to 30 every 2 to 3 months to receive group counseling, have a brief symptom screen, and receive prepacked medications. DMD comprises prepacking and distribution of medications to PuPs, which are at locations other than the clinic pharmacy. Patients only need to come to the clinic on a 6-monthly basis for a clinical exam and rescripting | 61% were aged 30–49 years | AC: 71% of patients were female. DMD: 70% female | HIV | Outcomes were retention and sustained viral suppression (<400 copies/mL) 12 months after AC or DMD enrollment (or comparable time for controls) | AC patients had higher 1-year retention (89.5% versus 81.6%, aRD: 8.3%; 95% CI: 1.1% to 15.6%) and comparable sustained 1-year viral suppression ((89.5% versus 81.6%, aRD: 8.3%; 95% CI: 1.1% to 15.6%) and comparable sustained 1-year viral suppression (<400 copies/mL any time <= 18 months) (80.0% versus 79.6%, aRD: 3.8%; 95% CI: −6.9% to 14.4%). Retention associations were apparently stronger for men than women (men RD: 13.1%, 95% CI: 0.3% to 23.5%; women RD: 6.0%, 95% CI: −0.9% to 12.9%). For DMD, 232 intervention and 346 control patients were enrolled; 71% of patients were female, 65% were aged 30–49 years, and median CD4 count at ART initiation was 270 cells/μL. DMD patients had apparently lower retention (81.5% versus 87.2%, aRD:−5.9%; 95% CI: −12.5% to 0.8%) and comparable viral suppression versus standard of care (77.2% versus 74.3%, aRD: −1.0%; 95% CI: −12.2% to 10.1%), though in both cases, our findings were imprecise. we found that ACs and DMD, in agreement with other studies, are feasible, acceptable, and for ACs, had positive outcomes. We saw an overall retention benefit to ACs and comparable outcomes with DMD against standard of care, which should still prove to be a benefit to clinics as they are designed to decongest the clinic overall | Effective adherence strategy |
| Adherence counseling and reminder text messages improve uptake of antiretroviral therapy in a tertiary hospital in Nigeria | Maduka et al., 2013 | Journal article | 2011 | Nigeria | ART non adherent patients | Tertiary health care institution in Nigeria | Randomized control trial among non-adherents | 104 | The intervention group received monthly adherence counseling and twice weekly short message reminders for four months, while the control group received only standard care. | Control: 35.3 (SD± 9.04) and intervention: 36.6 (SD± 11.77) | Females (56.7%) | HIV | Self-reported adherence: This was calculated based on client self-report of number of pills missed in the past seven days. The results for the intervention and control groups were compared to ascertain any changes in CD4 levels pre- and post-intervention and the magnitude of such changes if present. | Adherence counselling and text message reminders improved adherence among HIV patients. Its adoption for HIV patient management is advocated. At post-intervention, 76.9% of the intervention group and 55.8% of the control group achieved adherence ([sup] = 5.211, P = 0.022, RR = 0.75 (0.55-0.96), Cohen's w = 0.224). Also, median CD4+ cell count of the intervention group increased from 193 cells/ml to 575.0 cells/ml against 131.0 cells/ml to 361.5 cells/ml in the control group (P = 0.007). | Effective adherence strategy |
| Antiretroviral therapy in Zambia: do partners on ART enhance adherence? | Jones et al., 2014 | Journal article | Not specified | Lusaka, Zambia | HIV-positive serocon-cordant heterosexual couples. Couples in which either or both member were on ART | 6 Community Health Centres in urban Lusaka, Zambia | Longitudinal Implementation Science study | 446 | Influence of partners on each other's adherence and compared adherence between couples in which either one or both members were on antiretroviral therapy (ART) | 38 (SD± 8) | Not specified | HIV | Nonadherence was defined as any missed doses in the past 2 weeks. A longitudinal multivariable model was used to examine adherence including time (baseline, 6-, and 12- month follow-up), couple medication status (time varying; medication concordant versus medication discordant), and the interaction between time and medication status as predictors of interest. | At baseline, most participants were adherent to their medication (198 of 261 on medication, 76%). The proportion of adherent participants declined to 66% (143 of 217) at 6 months and returned to 70% (136 of 193) at 12-month follow-up. Adherence at baseline was not related to loss to follow up at 6 months (chi-square, χ2 = 1.25, P = .264) or 12 months (χ2 = 0.877, P = .349). Adherence also did not differ between individuals in HIV-sero-concordant and -sero-discordant couples Adherence was not enhanced by having a partner on ART, and that adherence declined over time. Partners on ART may not necessarily provide support for adherence to each other | Non-effective adherence strategy |
| Clinic-based food assistance is associated with increased medication adherence among HIV-infected adults on long-term antiretroviral therapy in Zambia | Tirivayi et al., 2012 | Journal article | 2009 | Lusaka, Zambia | ART patients receiving food assistance with a control group of non-recipients | Four Lusaka public-sector ART clinics that distributed food rations (Mtendere, Chawama, Kanyama, and George), and four control clinics that did not distribute rations (Bauleni, Chipata, Matero Reference, and Chilenje) | Cohort study | 291 | Clinic-based food assistance program: comparing ART patients receiving food assistance with a control group of non-recipients | Intervention: 41 (SD±0.8), Control: 40 (SD±0.6) | Female: intervention: 80% (115), control: 73% (107) | HIV | A comparison between ART adherence and the change in weight and CD4+ lymphocyte count between food insecure, HIV-infected Zambian adults on long-term treatment enrolled in a 6-month clinic-based food assistance program versus a matched control group which did not receive assistance. | The provision of food assistance to HIV infected adults on ART improved medication adherence. After 6 months, food assistance recipients (n=145) had higher ART adherence compared to non-recipients (n=147, 98.3% versus 88.8%, respectively; p<0.01). The improvement in adherence rates was greater for participants on ART for less than 230 days, and those with BMI<18.5 kg/m2, a higher HIV disease stage, or a CD4+ lymphocyte count ≤ 350 cells/μl | Effective adherence strategy |
| Community-based Adherence Clubs for the management of stable antiretroviral therapy patients in Cape Town, South Africa: a cohort study | Grimsrud et al., 2016 | Journal article | 2012-2013 | Cape Town, South Africa | Stable ART patients decentralized to community based adherence clubs | Gugulethu CHC-a large primary health care facility typical of urban public sector ART services across the region | Cohort study | 2113 | Community-Based Adherence Clubs: A CAC was a community-based, CHW led- and nurse-supported model of care supporting groups of 25 to 30 patients. CACs met every 2 months for group counseling, a brief symptom screening, and distribution of prepacked ART. | 33.4 (IQR 28.4–29.8) | Female (71%) | HIV | The outcomes of interest in this analysis were LTFU and viral rebound. LTFU was defined as having no visit in the first 12 weeks of 2014, and patients were censored at the date of last contact with either health care service. Viral rebound was defined as a single viral load measurement .1000 copies per milliliter after previous suppression (1000 copies/mL) | 94% were retained on ART after 12 months. CAC participation was associated with a 67% reduction in the risk of LTFU (aHR: 0.33, 95% CI: 0.27 to 0.40) compared with community health centre, and this association persisted when stratified by patient demographic and clinic characteristics. During the study period, 3.0% of CAC patients experienced viral rebound | Effective adherence strategy |
| Food assistance and its effect on the weight and antiretroviral therapy adherence of HIV infected adults evidence from Zambia | Tirivayi et al., 2010 | Journal article | 2009 | Lusaka, Zambia | HIV positive patients who are on ART | Four public-sector ART clinics distributed a standardized household food assistance ration | Cross sectional survey and administrative data | 314 | World Food Programme food assistance program: The WFP country programme aimed to improve the nutritional status and health of vulnerable populations through targeted assistance programs for people living with HIV/AIDS. | intervention: 41 (IQR 39.6-43), control: 40 (IQR 38.7-41.6) | Female: intervention: 80%, control: 71% | HIV | Weight and ART adherence over a period of 6 months among HIV-infected adults | The receipt of food assistance has significant and larger positive effect sizes on adherence to treatment for patients who had been on ART for less than the sample median of 995 days, while food assistance has no effect (ordinary least squares regression) or some negative effect (instrumental variable regression) on adherence for patients whose duration of ART was greater than the sample median | Effective adherence strategy |
| How treatment partners help: social analysis of an African adherence support intervention | O’Laughlin et al., 2011 | Journal article | 2006-2008 | Tanzania | People living with HIV | HIV public care setting | Qualitative study used a grounded theory approach. | 40 | Partner support | Average age: 40 years | Female (68%) | HIV | Highlights social consequences of treatment partnering and its significance for the health and well being of individuals living with HIV/AIDS in sub-Saharan Africa. | Ninety- eight minimally structured interviews were conducted with twenty pairs of adult HIV/AIDS patients (N = 20) and treatment partners (N = 20) treated at a public HIV-care setting in Tanzania. Four social functions were identified using inductive, category construction and interpretive methods of analysis: (1) encouraging disclosure; (2) com- bating stigma; (3) restoring hope; and (4) reducing social difference. These functions work to restore social connections and reverse the isolating effects of HIV/AIDS, strengthening access to essential community safety nets. Besides encouraging ARV adherence, treatment partners contribute to the social health of patients. Social health as well as HIV treatment success is essential to survival for persons living with HIV/AIDS in sub-Saharan Africa. | Effectiveness of adherence strategy is unclear |
| Improving ART adherence among HIV positive adolescents and youth using an eHealth intervention: a field study in Mombasa, Kenya | [Ivanova et al., 2019](https://go-gale-com.proxy.library.uu.nl/ps/advancedSearch.do?method=doSearch&searchType=AdvancedSearchForm&userGroupName=utrecht&inputFieldNames%5b0%5d=AU&prodId=AONE&inputFieldValues%5b0%5d=%22Olena+Ivanova%22) | Journal article | 2014 – end 2015 | Mombasa, Kenya | HIV positive patients who are on ART | Coast Provincial General Hospital Comprehensive Care Clinic (CCC) and Family Care Clinic (FCC) in Mombasa, Kenya | Cross-sectional study | 90 | Digital peer support platform aiming at improving adherence to ART treatment among HIV positive youth. The intervention consisted of interactive web-based peer support platform which included a blog with posts written by project coordinators, health care providers and young people on different topics related to sexual and reproductive health, HIV, medication, nutrition, relationships etc.; discussion section; Q&A section with health care providers; stories contest and private messaging. The platform resembled social media platforms with secured users' profiles for posting and communication. | 18.4 (SD±2.8) and range:15 to 25 years old | 36 were male and 45 were females | HIV | self-reported adherence | The participants were satisfied with the main features of the web platform and stated that they would use it again (95%). However, there was not a significant change in knowledge and behavior, but adherence intentions after 3 months intervention period have improved. | Effective adherence strategy |
| Improving clinic attendance and adherence to antiretroviral therapy through a treatment supporter intervention in Uganda: a randomized controlled trial | Kunutsor et al., 2011 | Journal article | 2010 | Jinja District, Uganda | HIV positive patients who are on ART | Jinja Hospital HIV clinic located in the Jinja District of central Uganda | Two-arm randomized controlled trial | 174 | Treatment Supporter (TS) intervention designed to improve overall access to ART in the context of resource-limited settings. Patients in the TS arm received both the TS intervention and the standard adherence intervention package. Elements of the standard intervention package consisted of self-monitoring of medication taking using adherence diaries; regular individual and group education by peer-workers using patient education leaflets and tabletop flip-charts; and late attendee tracing. The treatment supporters were usually family members—usually a partner, mother, daughter, sister, brother, friend, or neighbour/friend—who were chosen by the patient with the assistance of the health workers, had accepted the patient’s HIV? status and were confidantes. | 39.1 (SD±8.3) | Female: Treat supporter: 70.1%, non-treat supporter: 65.5% | HIV | Primary outcome measure was medication adherence for both arms of the study as measured by pill counts. Secondary outcomes were mortality, loss to follow-up, and clinic attendance outcomes categorised into attendance on or before day of appointment, within 3 days of appointment day, after 3 days of appointment day, and missed visits. | There was a non-significant difference in mean adherence between the TS and non-TS groups at end of follow-up [99.1% (95% CI: 98.3–99.9% vs. 96.3% (95% CI: 94.2–98.3%), P[0.05]. TS participants had more than four times the odds of achieving optimal adherence (C95%) [Odds ratio (OR) = 4.51, 95% CI: 1.22–16.62, exact P = 0.027]. TS participants were also more likely to be on time for their clinic appointments: 91.6 vs. 90.1% for TS and non-TS, respectively (OR = 1.19, 95% CI: 0.74–1.91, P[0.05). Use of patient-selected treatment supporters may be an effective intervention to improve ARV treatment outcomes in resource-constrained settings. | Non-effective adherence strategy |
| Improving treatment adherence for blood pressure lowering via mobile phone SMS-messages in South Africa: a qualitative evaluation of the SMS-text Adherence SuppoRt (StAR) trial | Leon et al., 2015 | Journal article | 2012-2014 | South Africa | Hypertensive patients | A single large public sector clinic in Cape Town, South Africa | A qualitative design using focus groups and in-depth interviews | 22 | The SMS-text message trial intervention: The intervention was a structured 12-month program of adherence support delivered by SMS-text message, intended to facilitate closer communication between patients and the health care system. Messages were designed to address a range of common potential issues with treatment adherence that might lead to changes in treatment adherence behaviour and improve health outcomes. | Age range: 36-78 years old | 16 were females and 6 were males | HTN | Self-reported adherence: For the sub-group of participants who reported adherence behaviour change, there are indications that the intervention may have operated in multiple ways to facilitate change | Most participants were comfortable with the technology of using SMS-text messages. Messages were experienced as acceptable, relevant and useful to a broad range of participants. The SMS-content, the respectful tone and the delivery (timing of reminders and frequency) and the relational aspect of trial participation (feeling cared for) were all highly valued. A subgroup who benefitted the most, were those who had been struggling with adherence due to high levels of personal stress. The intervention appeared to coincide with their readiness for change, and provided practical and emotional support for improving adherence behaviour. Change may have been facilitated through increased acknowledgement of their health status and attitudinal change towards greater self-responsibility. | Effective adherence strategy |
| Integrated mobile phone interventions for adherence to antiretroviral treatment in clients with HIV infection in Accra, Ghana | Dzansi, 2017 | Doctoral dissertation | 2017 | Accra, Ghana | HIV positive patients who are on ART | 2 major health facilities Greater Accra | Mixed method: Phase 1-RCT: quantitative analytical experimental study, Phase 2: Qualitative study approach | 362 | The mobile phone intervention on adherence in two groups (Intervention and Control). The Control group received standard care while the Intervention group received standard care, alarm prompting, weekly text messages and monthly voice calls. | 44.4 (SD±9.8) | Female 228 (63%) | HIV | The primary outcome indicator for the intervention was adherence scores. The secondary indicators were BMI, CD4 counts levels. Primary (overall adherence: Self-report, visual analogue, pill identification, pill count) and secondary (CD4 count and Body Mass Index) outcomes were measured at baseline, month three and month six. In phase two, individual interviews were conducted with six clients and two health professionals; three focus group discussions were held with participants from the Intervention group at month six. | Respondents were highly adherent at baseline (n =255, 70%) month three (n =176, 80%) and month six (n = 180, 67%). Overall adherence outcome for the three timelines in the Intervention (M = 99.2, SE = .059, CI = 99.1, 99.4) and Control (M = 99, SE = .066, CI = 98.9, 99.2) groups was statistically significant F (1, 2547) = 4.24, p = .04. The observed change occurred in both groups, therefore not attributable to the treatment. Intervention was rated as helpful and qualitative outcomes show a readiness for integration of mobile phone in care. | Non-effective adherence strategy |
| Intensive adherence counselling for HIV-infected individuals failing second-line antiretroviral therapy in Johannesburg, South Africa | Fox et al., 2016 | Journal article | between 1 March 2012 and 1 December 2013. | Johannesburg, South Africa | Patients on second-line ART | Themba Lethu Clinic-a public-sector clinic in Johannesburg | Single-arm prospective cohort study | 400 | Intensive adherence counselling for HIV-infected individuals failing second-line antiretroviral therapy. Patients with an elevated viral load (≥400 copies/ml) on second line are flagged by clinic staff, bypass the normal clinic queue and undergo detailed counselling with an experienced adherence counsellor or social worker trained to address common misconceptions about ART. Counsellors use a standardised form that includes a depression screen, alcohol and substance abuse evaluation, and an assessment to help identify barriers to adherence. The form includes questions on side effects of medications, the patient’s social circumstances, the patient’s employment status and depression indicators. All patients also complete a standardised adherence screen. | 40.8 (IQR 36.2–46.7) | Female 249 (62%) | HIV | Primary outcome was a suppressed viral load at the first viral load measure after targeted adherence counselling. Follow-up began at the first elevated viral load on a PI and ended at the earliest of death, loss to follow-up, transfer | Of the 400 patients who underwent targeted adherence counselling after an elevated viral load on second-line ART, 388 (97%) underwent repeat viral load testing. Most of these (n = 249; 64%, 95% CI 59–69) resuppressed (400 copies/ml, 11 switched to third line, 5 were awaiting third line, 4 had died and 13 were lost to follow-up. Among the unsuppressed, 48 successfully underwent resistance testing with some resistance detected in most (41/48). In addition with intensive counselling, a limited numberof patients required expensive resistance testing. Due tothe lack of a control group, the effectiveness of theseinterventions cannot be determined. | Effectiveness of adherence strategy is unclear |
| Internet and cell phone as tools to support antiretroviral therapy adherence among HIV infected patients aged above 18 years attending Kenyatta national hospital | Kinyua, 2015 | Masters degree dissertation | 2015 | Kenya | HIV infected patients on ART at Kenyatta National Hospital comprehensive care clinic. | Kenyatta National Hospital, Kenya | Descriptive cross-sectional study | 385 | HIV infected patients received weekly SMS messages from a clinic nurse and were required to respond within 48 hrs. Patients in the control group received standard follow-up without text messages. | mean age: 40.3 years | Females 237 (62%) | HIV | Primary outcomes were self-reported ART adherence (>95% of prescribed doses in the past 30 days at both 6- and 12-month follow-up visits) and plasma HIV-1 viral RNA load suppression | Nearly all (99%) of the HIV infected clients interviewed supported the idea of cell phone use in improving adherence to their medication. Majority (98%) of the xviii respondents reported that internet was not a feasible tool in supporting ARV adherence due to its high cost of maintenance and use. The study concluded that there was higher accessibility of the participants to cell phone than internet and the high usage of cell phones for communication through calling and texting, while high usage of internet for communication through social media networks. Cell phone was identified as a feasible tool for supporting adherence to ARV therapy due to its affordability and easy access. The study therefore recommends adoption of cell phone into health care sector to boost medication adherence. | Effective adherence strategy |
| It helps me live, sends my children to school, and feeds me: a qualitative study of how food and cash incentives may improve adherence to treatment and care among adults living with HIV in Tanzania | Czaicki et al., 2017 | Journal article | Interviews were conducted between February and May 2015. | Shinyanga, Tanzania | HIV positive patients who are on ART experiencing food insecurity | Two government hospitals and one government health clinic | Qualitative study | 29 | Food and cash incentives to improve adherence to ART among food-insecure HIV patients: nutritional assessment and counselling (NAC; control), NAC plus monthly food incentive, or NAC plus cash incentive. Participants in the food or cash arms were eligible for up to 6 monthly incentives of equivalent value conditional on attending routine appointments (within a 4-day window). | Not Specified | 16 women and 13 men | HIV | Retention in care and adherence to antiretroviral therapy (ART) | We found that the incentives acted through three pathways to potentially increase retention in care and adherence to ART: 1) addressing competing needs and offsetting opportunity costs associated with clinic attendance, 2) alleviating stress associated with attending clinic and meeting basic needs, and 3) by potentially increasing motivation. Participants did not report any harmful events associated with the incentives but reported myriad beneficial effects on household welfare. | Effective adherence strategy |
| Mobile phone technologies improve adherence to antiretroviral treatment in a resource-limited setting: a randomized controlled trial of text message reminders | Pop-Eleches et al., 2011 | Journal article | between June 2007 and August 2008 | Kenya | Adult patients on ART | Chulaimbo Rural Health Center (CRHC) in Nyanza Province, Kenya. | Randomized controlled trial | 431 | Mobile phone technologies: four SMS reminder interventions with 48 weeks of follow-up. Participants in the intervention groups received SMS reminders that were either short or long and sent at a daily or weekly frequency. | Average age: 35.65 (N =137) | Female (66%) | HIV | Adherence was measured using the medication event monitoring system. The primary outcome was whether adherence exceeded 90% during each 12- week period of analysis and the 48-week study period. The secondary outcome was whether there were treatment interruptions lasting at least 48 h. | In intention-to-treat analysis, 53% of participants receiving weekly SMS reminders achieved adherence of at least 90% during the 48 weeks of the study, compared with 40% of participants in the control group (P=0.03). Participants in groups receiving weekly reminders were also significantly less likely to experience treatment interruptions exceeding 48 h during the 48- week follow-up period than participants in the control group (81 vs. 90%, P = 0.03). These results suggest that SMS reminders may be an important tool to achieve optimal treatment response in resource-limited settings. | Effective adherence strategy |
| Mobile phone text messages to support treatment adherence in adults with high blood pressure (SMS-Text Adherence Support [StAR]) a single-blind, randomized trial | Bobrow et al., 2016 | Journal article | Between June 26, 2012, and November 23, 2012, | Cape Town, South Africa | Hypertensive patients | large public sector clinic in Cape Town, South Africa | A parallel, three-group randomized controlled trial | 1372 | Two SMS text-messaging based interventions with clinical staff, and patients with high blood pressure working and living in low-income communities around Cape Town.12 The messages were designed to address a range of common issues with adherence to and persistence with treatment.13 We developed a library of SMS-text messages, which we mapped to a taxonomy of behavior change techniques. Most of the messages focused on the techniques of goals and planning, repetition and substitution, social support, and natural consequences. The SMS text-messages used in the interventions were developed, translated, and tested in English, isiXhosa and Afrikaans, the three languages most commonly spoken by people living in Cape Town. | 54.3 (SD±11.5) | Sex (male) usual care:126 (28%), information only:126 (28%), Interactive: 127 (28%) | HTN | The primary clinical outcome was the change in mean SBP measured at baseline and twelve months with a validated oscillo metric device,10 adapted to record six sequential readings at three-minute intervals. The mean blood pressure was calculated by discarding the initial reading and calculating the mean from the five remaining readings. | There was a small, reduction in systolic blood pressure control compared to usual care at 12-months. There was no evidence that an interactive intervention increased this effect.1372 participants were randomized to receive information-only SMS text-messages (n=457), interactive SMS text messages (n=458), or usual care (n=457). Primary outcome data were available for 1256 (92%) participants. At 12-months, the mean adjusted change (95% CI) in systolic blood pressure compared to usual care was −2.2 mm Hg (−4.4 to −0.04) with information-only SMS and −1.6 mm Hg (−3.7 to 0.6) with interactive SMS. Odds ratios (95% CI) for the proportion of participants with a blood pressure | Non-effective adherence strategy |
| Peer mentors, mobile phone and pills: collective monitoring and adherence in Kenyatta National Hospital's HIV treatment programme | Moyer, 2014 | Journal article | between 2008 and 2014 | Kenya | HIV positive patients who are on ART | Large public sector urban ART outpatient clinic | In-depth ethnographic case study | Not Specified | A text message sent to the designated peer mentor who comes to the Voluntary Counselling center and escorts the HIV patient to the Comprehensive Care Centre, where the file is established, and the client is informed about further laboratory tests and treatment option. The exchange of tele-phone numbers also gave expert clients a way to follow up with the newly diagnosed patients, providing a means of monitoring their health, their entry into medical care, and their adherence. Having the mobile number of a trusted expert client at the treatment centre allowed HIV-positive clients to coordinate the logistical details of hospital visits and drug pick-ups, as well as elicit information and advice about the daily challenges of living with HIV, which spared them additional trips to the clinic. | Not Specified | Not specified | HIV | No outcome measure | Peer mentors provide counselling services, follow up people who stray from treatment regimens, and perform a range of other tasks related to patient management and treatment adherence | Effectiveness of adherence strategy is unclear |
| Promoting adherence to antiretroviral therapy through a directly administered antiretroviral therapy (DAART) strategy in Mombasa Kenya. | Sarna et al., 2005 | Journal article | between September 2003 and November 2004 | Mombasa, Kenya | HIV positive patients who are on ART | Coast Province General Hospital, Mkomani Bomu Clinic, and Port Reitz District Hospital in Mombasa, Kenya | Two-arm randomized controlled trial | 234 | A directly administered antiretroviral therapy strategy (DAART). The DAART intervention lasted for a period of 24 weeks. During this time, participants in the DAART arm visited a health centre twice a week where they met with DAART observers (nurses) who observed the ingestion of one dose of antiretroviral medications, performed pill-counts, collected used medication bottles, enquired about difficulties encountered, and provided individualized adherence support. At these visits medications were dispensed for the following three or four days, until the next visit. During the DAART intervention, community health workers (CHWs) traced participants who missed visits and carried medications home for those who, for reasons of ill-health, were unable to visit the center. After the first 24 weeks, DAART patients were followed by routine monthly visits for a further 48 weeks. | 37 (IQR 20–58) | Female (64%) | HIV | Mean adherence levels compared to those who received standard follow-up. Adherence levels in excess of 95 percent over 24 weeks consistently at each reporting period as compared to those receiving standard follow-up. differences in CD4 counts and weight between the two groups at 24 weeks of follow-up. | Data from pill counts show that mean adherence over 24 weeks was significantly higher in the DAART group compared to the non-DAART group (96 percent vs. 90 percent; p = 0.042). Data from pill counts also show that a greater proportion of DAART clients achieved a total adherence > 95 percent over 24 weeks than non-DAART clients (92 percent vs. 80 percent; p = .012). igh levels of adherence to HAART were observed for all patients during the first 24 weeks of the regimen. However, patients exposed to the DAART intervention achieved higher mean adherence levels compared to those who received standard follow-up. A higher proportion of DAART patients achieved adherence levels in excess of 95 percent over 24 weeks consistently at each reporting period as compared to those receiving standard follow-up. | Effective adherence strategy |
| Real-time electronic adherence monitoring plus follow-up improves adherence compared to standard electronic adherence monitoring | Haberer et al., 2017 | Journal article | Participants were drawn from a observational cohort from 2005–2011, followed by real-time EAM from 2011–2015. Cohort enrolment occurred through 2012. Some participants were therefore monitored with both types of EAM; others were monitored only with real-time EAM. | Uganda | HIV positive patients who are on ART |  | Ad-hoc analysis of a cohort study | 112 | In electronic adherence monitoring (EAM), a device records each opening with a date-and-time stamp as a proxy for medication ingestion. Standard EAM devices store these data for later transfer to a computer. EAM was followed subsequently by home visits for sustained adherence interruptions. | Median age: 36 years (IQR not specified) | Females (68%) | HIV | Differences in overall adherence and sustained adherence interruptions between these two periods | Real-time EAM with follow-up triggered by incomplete adherence is an effective intervention. Follow-up visits were not designed as interventions; however, participants likely perceived them as supportive. Immediately after switching from standard EAM to real time EAM plus follow-up, mean adherence increased from 84% to 93%. | Effective adherence strategy |
| Short message service (SMS) reminders and real-time adherence monitoring improve antiretroviral therapy adherence in rural Uganda | Haberer et al., 2016 | Journal article | Between September 2013 and October 2014, | Uganda | HIV positive patients who are on ART | Mbarara Regional Referral Hospital in southwestern Uganda | Pilot randomized controlled trial. |  | All study participants received a real-time adherence monitor and were followed for 9 months. Monitor openings during periods of inadequate cellular reception were stored for later transmission. Participants were given solar chargers and sent an SMS to charge the monitor as needed. | Median age: 31 years (IQR not specified) | Females (65%) | HIV | The primary outcome of interest was adherence (calculated as the number of monitor opening signals received divided by the number of monitor opening signals expected, and capped at 100%). Scheduled SMS reminders improved ART in the context of real-time monitoring. Larger studies are needed to determine the impact of triggered reminders and role of social supporters in improving adherence. | Scheduled reminders were experienced as supportive, whereas triggered reminders could be received too late to be effective (i.e., after participants were asleep) Although prior studies saw significant benefit in overall adherence and adherence lapses with SMS linked to real-time detection of late or missed doses. Potential explanations include the overall high adherence in this study and the small sample size that scheduled SMS significantly increased adherence in individuals initiating ART in Uganda. The technology employed for real-time adherence monitoring and accompanying SMS was successfully implemented despite limited resources. Scheduled SMS reminders improved ART in the context of real-time monitoring. Larger studies are needed to determine the impact of triggered reminders and role of social supporters in improving adherence | Effective adherence strategy |
| The Cameroon Mobile Phone SMS (CAMPS) trial: a randomized trial of text messaging versus usual care for adherence to antiretroviral therapy | Mbuagbaw et al., 2012 | Journal article | 2010-2011 | Cameroon | HIV-positive adults on ART (aged 21 years and above) | Yaoundé Central Hospital (YCH) Accredited Treatment Centre (ATC). | Single-site randomized two-arm parallel design trial | 200 | The use of motivational mobile phone text messages (SMS) to improve adherence to antiretroviral therapy (ART) over six months. A short text message was send to each participant in the intervention (SMS) group, once a week. The content of the message was motivational, with a reminder component. The content was varied and contemporary (e.g. messages would contain season's greetings) so as to retain participants' attention throughout the study period and to explore the various aspects of behavior change. Text messaging was an add-on to usual care that includes regular ART counselling and home visits determined on a case-by-case basis. In the control (no SMS) group, participants received only usual care. They did not receive any text messages, but they were interviewed at baseline, 3 months and 6 months. | SMS group: 41.3 (SD±10.1), Control group: 39.0 (SD±10.0) | Female; SMS 69 (68.3%) vs Control 78 (78.8%) | HIV | The primary outcome was adherence measured using a visual analogue scale (VAS), number of doses missed (in the week preceding the interview) and pharmacy refill data. Outcomes were measured at 3 and 6 months. Service providers and outcome assessors were blinded to allocation. To conclude, standardized motivational mobile phone text messages did not significantly improve adherence to ART in this study. Other types of messaging or longer term studies are recommended | Analysis was by intention-to-treat. Between November and December 2010, 200 participants were randomized, with 101 in the intervention group and 99 in the control group. At 6 months, overall retention was 81.5%. We found no significant effect on adherence by VAS>95% (risk ratio [RR] 1.06, 95% confidence interval [CI] 0.89, 1.29; p = 0.542; reported missed doses (RR 1.01, 95% CI 0.87, 1.16; p>0.999) or number of pharmacy refills (mean difference [MD] 0.1, 95% CI: 0.23, 0.43; p = 0.617. One participant in the intervention arm reported a possible disclosure of status. | Effective adherence strategy |
| THE EFFECT OF ADHERENCE COUNSELLING AND SHORT MESSAGE SENDING (SMS) REMINDERS ON ADHERENCE TO TREATMENT IN CLIENTS ON HIGHLY ACTIVE ANTIRETROVIRAL THERAPY IN RIVERS STATE | Maduka, 2011 | A dissertation | 2011 | River state, Nigeria | HIV positive patients who are on ART | Two HAART treatment facilities were selected the University of Port Harcourt Teaching Hospital (UPTH) and the Health of the Sick Catholic Hospital (HOSH) in Port Harcourt. | Experimental study, employing a two site, two group randomized control trial. | 104 | The effect of adherence counselling and short message sending reminders as interventions to improve adherence to ART. A. Intervention Group: 1.Adherence Counselling: One adherence counselling session per month for four consecutive months was conducted for each client Each session lasted between 45 and 60 minutes. 2.Short Message Sending (SMS) reminders Twice a week (Monday and Thursday morning) for the duration of four months, each client enrolled into the intervention group of the study was sent a pre scripted text message containing adherence related information and a reminder to take HAART medications. B. Control group - The control group received standard care only. They did not get any adherence counselling and SMS message reminders. | 35.94 (SD±14.9), range: 20-68. | Females (56.7%) | HIV | Self-Reported Adherence: This was calculated based on client self-report of number of pills missed in the past seven days. A cut off of 95% was used to distinguish those who were adherent from those who were not. Adherence was assessed at recruitment (before the commencement of the intervention), at every monthly counselling visit and at the end of the intervention in the fourth month. However, the two adherence values used for comparison were the pre-intervention and post intervention adherence rates in the intervention and control groups. CD4+ Ce ll Count: Pre and post intervention CD4 cell counts were carried out for all study participants. The results for the intervention and control groups were compared to ascertain any changes in CD4 levels pre and post intervention and the magnitude of such changes if present. CD4 count has been shown to be useful as a biological measure of response to HAART treatment which is dependent among other things on adherence to treatment. | At the end of the intervention period, adherence was significantly higher in the intervention group (76.9%) than in the control group (55.8%) (p=0.022) with a small effect size (Cohen’s w) of 0.224 and RR of 0.75 (0.55 to 0.96). In addition, the mean CD4+ cell counts for the intervention group (574.15 cells/ml) was significantly higher than that of the control group (408.58 cells/ml) (p=0.005) with a medium effect size (Cohen’s d) of 0.560. The responses from the focus group discussions corroborated the findings from the quantitative analysis and shed light on the perceptions of patients as to the impact of these interventions. | Effective adherence strategy |
| The effect of food assistance on adherence to antiretroviral therapy among HIV/AIDS patients in Sofala province, in Mozambique: a retrospective study | Posse et al., 2013 | Journal article | September 2007 to December 2010) | Sofala, Mozambique | HIV/AIDS positive patients of all ages, including children, tuberculosis patients and pregnant and breast-feeding women enrolled in prevention of mother-to-child transmission (PMTCT) programs. | Five districts in Sofala province (Beira, Dondo, Nhamatanda, Caia and Muanza) with similar patients, who did not receive food assistance. Controls-from four districts in Zambezia province (Quelimane, Namacurra, Nicoadala and Mopeia). | Retrospective study | 357 | Provision of food assistance for HIV/AIDS patients in Sofala Province. This intervention was provided to HIV/AIDS patients of all ages, including children, tuberculosis patients and pregnant and breast-feeding women enrolled in prevention of mother-to-child transmission (PMTCT) programs | FA recipients 38.52 (SD±9.06) vs controls 36.61 (SD±10.20) | Female Fa recipients 134 (77.91%) vs controls 125 (67.57%) | HIV | Adherence based on pill pick-up, a pharmacy adherence measure (PAM), which measures whether an individual picks-up all or a majority of their prescribed ART. PAMs are ideally suited to monitoring adherence because they are objective and can be easily derived from data routinely collected for other purposes, such as clinical care or drug supply management | During the food assistance programme, the adherence of food assistance recipients who received food assistance for a period of six and 12 months and non-food assistance recipients is not significantly different as the average impact is only 0.4% (p=0.94) and -2.3% (p=0.73) respectively. For the period after food assistance had been terminated, adherence is still not significantly different between the two groups, as the average impact is 5.3% (p=0.44) and 1.9% (p=0.65). | Non-effective adherence strategy |
| The Effect of Home Follow Up Visit in Enhancing Antiretroviral Therapy Adherence Among HIV and AIDS Patient in a Rural Setting, Malawi | Mwale et al., 2016 | Journal article | from 2009 January to December 2010 | Malawi | The study populations were HIV patients, who had been on Triomune (combination of Stavudine, Lamivudine and Nevirapine) continuously for more than one year from the period of being initiated on antiretroviral drugs, and were living within the catchment area of the two Mission Hospitals namely St Gabriel's and Kapiri. | St Gabriel's and Kapiri Mission Hospitals in Lilongwe and Mchinji districts, Malawi | An Operational Evaluation Quantitative Descriptive Study | 589 | Comparing the level of adherence to ART between patients who receive ambulatory treatment at a health facility with a home visit program and those receiving treatment at a health facility without home visit program. | **Not Specified** | Female: St Gabriel's 55 (51%) and Kapiri Hospitals (47%) | HIV | No clear outcome measure documented. Levels of ART adherence in patients who were not attached to treatment helpers were assessed. | The Chi square test results showed that there was a significant difference in the ART adherence between HIV patients who received ambulatory treatment at health facility with no follow up visit (Kapiri) and that of a follow up visit (St Gabriel's). χ2 = 21.02, p=0.001, α=0.05 with effective size = 0.189. Follow up of clients by treatment helpers within the community can help to improve ART adherence and retention of clients on ART. | Effective adherence strategy |
| The experience of "medicine companions" to support adherence to antiretroviral therapy: quantitative and qualitative data from a trial population in Uganda | Foster et al., 2010 | Journal article | 2005-2008. | Uganda | HIV positive patients | AIDS Support Organization (TASO) Clinic in Jinja, southeast Uganda | Randomized controlled trial | 1453 | Patients were randomized to either home based (HB) or facility based (FB) care, using cluster randomization. Patients receiving HB care visited monthly by a field officer on a motorcycle. HB care patients visit the TASO clinic every six months for a routine clinical and counselling review. FB patients visit the clinic every month to collect drugs and be seen by a nurse; they are assessed routinely every three months by a counsellor and physician. Patients in both arms visited the TASO clinic any time they felt unwell, and they had access to a telephone hotline. In the FB arm, patients eligible for ART were given Voluntary Testing and Counseling (VCT) vouchers for each family member and for the medicine companion (MC) and were encouraged to bring them to the facility for free VCT. In the HB arm, ART patients were visited by TASO field officers and VCT was provided at their homes to household members and to the MC if requested. All patients were requested to appoint a MC to assist them in remembering to take their medicines and to encourage them when they are not feeling well. In the FB arm, eligible patients were asked to come along with their MCs on their enrolment visit. This was a condition for ART initiation. | The mean age of the MCs: 30.8 years and the median is 30 (33.8 for men, 29.5 for women), but the mode is 14) | Female (71%) | HIV | The qualitative study sample was stratified to ensure equal numbers of participants by sex, trial arm, and clinical/ immunological stage contrasting early (CD4 counts above 150106 /l or Stages I and II defining conditions) and advanced (CD4 counts below 100106 /l or Stage III and IV defining conditions) categories. The patients were asked about the role of their MC, how they chose their MC, and how helpful the MC was in their therapy. Textual data on rationales and experiences with MCs were transcribed, translated, coded and analyzed by two independent reviewers. This paper reports on the data collected at baseline. Characteristics of MCs in both arms of the study, and on the qualitative data about MCs collected through in-depth interviews at baseline, months 3, 6, and 36 (no questions on the MC were asked at the 18th month interview). | Women were most likely to choose a child as their MC while men were most likely to choose their spouse; 41% of women chose an MC under 21 compared with only 14% of men. Only 31% of married women chose their husband, compared with 66% of married men who chose their wife. Qualitative interviews suggested MCs proved useful for reminding and other supportive tasks in the first three months but were generally less essential by six months and beyond. Convenience, reliability, and trust were key considerations in choosing an MC. Children provided the only alternative for many unmarried women, but even some married women felt children made more reliable MCs than husbands. Participants who had disclosed their serostatus usually received drug-taking reminders from multiple household members. One participant in the qualitative sample with poor family relations delayed starting treatment due to unwillingness to identify an MC. MCs were generally welcome and useful in supporting early adherence. However, disclosure to an MC should not be a condition of obtaining treatment | Effectiveness of adherence strategy is unclear |
| The impact of community-versus clinic-based adherence clubs on loss from care and viral suppression for antiretroviral therapy patients: Findings from a pragmatic randomized controlled trial in South Africa | Hanrahan et al., 2019 | Journal article | From February 12, 2014, to May 31, 2015 | South Africa | HIV positive patients who are virally suppressed on antiretroviral therapy (ART) | Witkoppen Health and Welfare Centre in Johannesburg, South Africa | Pragmatic randomized controlled trial | 775 | Adherence clubs, where groups of 25–30 patients who are virally suppressed on antiretroviral therapy (ART) meet for counselling and medication pickup, represent an innovative model to retain patients in care and facilitate task-shifting. This intervention replaces traditional clinical care encounters with a 1-hour group session every 2–3 months and can be organized at a clinic or a community venue. We randomized eligible adults into pairs of clubs—376 (49%) into clinic-based clubs and 399 (51%) into community-based clubs. | median age: 38 years | Female (65%) | HIV | The primary outcome was loss from club-based care, defined as referral to clinic-based standard care for any of the above specified reasons. Participants were assessed for the outcome at each club visit, each annual medical visit and any interim clinical visit made between medical visits. The primary outcome was assessed through review of the club register and review of the participants’ clinical files and electronic medical records. Key prespecified secondary outcomes were the proportion of patients who voluntarily chose to return to clinic-based standard care, the proportion of patients with medical contraindication for continuation of club-based care (those referred back to clinic-based care because of pregnancy, TB diagnosis, hypertension, identification of an excluding comorbid or chronic condition, or ART regimen change), and all-cause mortality. Participants were followed for outcomes for 24 months following the initial treatment assignment, and outcomes were compared by arm. | Overall, 47% (95% CI 44%–51%) experienced the primary outcome of loss from club-based care. Among community-based club participants, the cumulative proportion lost from club-based care was 52% (95% CI 47%–57%), compared to 43% (95% CI 38%–48%, p = 0.002) among clinic-based club participants. The risk of loss to club-based care was higher among participants assigned to community-based clubs than among those assigned to clinic-based clubs (adjusted hazard ratio 1.38, 95% CI 1.02–1.87, p = 0.032), after accounting for sex, age, nationality, time on ART, baseline CD4 count, and employment status. Among those who were lost from club-based care (n = 367), the most common reason was missing a club visit and the associated ART medication pickup entirely (54%, 95% CI 49%–59%), and was similar by arm (p = 0.086). Development of an excluding comorbidity occurred in 3% overall of those lost from club-based care, and was not different by arm (p = 0.816); no deaths occurred in either arm during club-based care. Viral rebound occurred in 13% of those lost from community club-based care and 21% of those lost from clinic-based care (p = 0.051). In post hoc secondary analysis, among those referred to standard care, 72% (95% CI 68%–77%) reengaged in clinic-based care within 90 days of their club-based care discontinuation date. The main limitations of the trial are the lack of a comparison group receiving routine clinic-based standard care and the potential limited generalizability due to the single-clinic setting. | Non-effective adherence strategy |
| The Meanings in the messages: how SMS reminders and real-time adherence monitoring improve antiretroviral therapy adherence in rural Uganda | Ware et al., 2016 | Journal article | September 2013 and June 2015 | Uganda | HIV positive adult patients who are on ART | Mbarara Regional Referral Hospital (MRRH), Mbarara, in rural southwestern Uganda. | Qualitative study, conducted with a pilot randomized controlled trial. | 63 | The study investigated the effects of multiple types of SMS reminders combined with real-time adherence monitoring on ART adherence. All study participants were given a real-time adherence monitor and were randomized (1 : 1 : 1) to receive ‘scheduled’ SMS reminders (daily, weekly), reminders ‘triggered’ by a missed dose, or no SMS (a control group). | 30 (IQR 25-35) | Female (65%) | HIV | Category development began with repeated reviews of coded data to populate one a-priori category (SMS reminder preferences), and to identify emergent themes. Subsequent steps included specifying labels, organizing, and writing descriptions of category content, and supplying evidence from the data in the form of illustrative quotes from interviewees. | SMS reminders prompted taking individual doses of antiretroviral therapy and helped to develop a ‘habit’ of adherence. Real-time adherence monitoring was experienced as ‘being seen’; participants interpreted ‘being seen’ as an opportunity to demonstrate seriousness of commitment to treatment and ‘taking responsibility’ for adherence. Both SMS reminders and real-time monitoring were interpreted as signs of ‘caring’ by the healthcare system. Feeling ‘cared about’ offset depressed mood and invigorated adherence. | Effective adherence strategy |
| The role of social support on HIV testing and treatment adherence: A qualitative study of HIV-infected refugees in southwestern Uganda | Rouhani et al., 2017 | Journal article | from March to July 2011 | Uganda | HIV-infected refugees on anti-retroviral therapy (ART) in Uganda | GIZ clinic in Nakivale refugee settlement in Southwestern Uganda | Qualitative study | 61 | Four roles for the types of social support were identified: (1) informational support encouraged refugees to test for HIV; (2) emotional support helped refugees cope with a diagnosis of HIV; (3) instrumental support facilitated adherence to ART and (4) after diagnosis, HIV-infected refugees provided informational and emotional support to encourage other refugees to test for HIV. | Average agr was 40 years old | Female (59%) | HIV | The categories that emerged fit within four commonly acknowledged types of social support: emotional support, informational support, instrumental support, and appraisal support. Emotional support was defined as the conveyance of love, caring, trust and acceptance. Informational support was information that helped solve problems and explain circumstances, such as advice and suggestions. Instrumental support was tangible and involved the donation of goods and services. Appraisal support involved assistance with self-evaluation. Finally, the data were classified into conceptual categories of social support that the participant received from others (informational support, emotional support, and instrumental support) versus social support the participant offered to others. | Our data indicate that, for refugees in Nakivale on ART, social support has a profound influence on HIV testing and adherence to medications. In our study population, informational support encouraged refugees to test for HIV and thus access care. Emotional support helped patients cope with an HIV diagnosis, and instrumental support helped them adhere to HIV treatment. Additionally, patients felt they could encourage testing by providing informational and emotional support to others. Taken together, our findings suggest that, in Nakivale, social support among refugees plays a similar role in determining HIV testing and treatment adherence compared to non-refugee populations. This similarity does not diminish the importance of these findings but rather suggests an opportunity to explore how social support interventions successful in other settings can be modified to the refugee context. In this study, informational support encouraged patients to test, and the patients in turn offered similar support to encourage others to test. The results of our study suggest that HIV-infected refugees might also encourage testing uptake by making it more salient and acceptable to their social contacts. | Effective adherence strategy |
| The use of a brief, active visualisation intervention to improve adherence to antiretroviral therapy in non-adherent patients in South Africa | Jones et al., 2019 | Journal article | May and November 2016 | South Africa | Non-adherent HIV positive patients on ART | Two sites in the Western Cape South Africa; Infectious Diseases Clinic at a major peri-urban hospital and a community clinic | Randomized controlled trial | 111 | The intervention was an active visualisation device. The intervention was delivered by the trained research assistants. The intervention took approximately 10 minutes with each participant. The intervention runs through scenarios to demonstrate that medication must be taken each day, as even when medication is added to the ‘body’, the following day the pink colour returns because the infection cannot be reversed. This process demonstrates why consistent adherence is needed to achieve virological suppression. The intervention also demonstrates the effects of missing one or two doses of ART, versus how long-term non-adherence can lead to treatment failure. | Mean: 36.48 (SD±9.66), range: 15 to 59 years | Female (75/11, 67.6%) | HIV | The primary outcome was adherence as measured by plasma viral load (VL). | There was a clinically significant difference (p = 0.06) in VL change scores between groups from baseline to follow-up, where the intervention had a greater decrease in log VL (Madj = − 1.92, CI [− 2.41, − 1.43), as compared to the control group (Madj = − 1.24, [− 1.76, − 0.73]). Participants in the intervention group were also significantly more likely to have a 0.5 log improvement in VL at follow-up (𝜒2(1) = 4.82, p = 0.028, ɸ = 0.28). | Effective adherence strategy |
| Treatment partners and adherence to HAART in Central Mozambique | Stubbs et al, 2009 | Journal article | between September 2004 and June 2006 | Mozambique | HIV positive patients on ART patients with good adherence and suboptimal adherence | Beira Day Hospital pharmacy, Mozambique | Randomized controlled trial | 896 | Treatment partners: Prior to starting HAART, patients are encouraged to self-select a treatment partner, such as a spouse, family member, or friend. If a treatment partner cannot be identified, clinic staff may suggest a treatment partner who is either a peer counsellor based in the clinic or a member of a community-based group of people living with HIV/AIDS. | 15 - 29 (24.5%), 30-39 (40.6%), 40-49 (24.2%), 50> (10.7%) | Female (54.2%) | HIV | Adherence rates was calculated based on pharmacy records for all patients who refilled their medication for at least six consecutive months between September 2004 and June 2006. Differences in baseline characteristics for patients with high (≥90%) adherence versus patients with low (<90%) adherence were assessed to identify factors that may be associated with high and low adherence, respectively, in this study population | A total of 305 patients (70%) had self-selected treatment partners, 121 (28%) had community-based treatment partners, and 8 (2%) had no treatment partner. In adjusted analysis, patients who had no treatment partner were more likely to have low adherence (OR 9.47; 95% confidence interval 2.37–37.86 compared to self-selected treatment partner). Patients with community-based treatment partners did not have significantly lower adherence than patients with self-selected treatment partners. | Effective adherence strategy |
| Treatment supporter to improve adherence to antiretroviral therapy in HIV-infected South African adults: a qualitative study | Nachega et al., 2006 | Journal article | July 1 to August 30, 2004 | South Africa | HIV positive adult patients who are on ART | HIV primary care settings: 3 clinics in Khayelitsha and 1 clinic in Gugulethu | Exploratory qualitative pilot study | 19 | This study aimed to understand how patient-selected treatment supports might affect antiretroviral treatment outcomes and to identify key components of support, including the social and material resources necessary for promoting high adherence in South Africa. | Range: 22-42 years | Female 11, Male 1 | HIV | Information was collected from patients (insiders) and health care providers (outsiders) involved in HAART programs to determine the characteristics of effective treatment support and to learn more about social and material barriers to HAART adherence. The transcripts were used for content analysis to identify main themes and were then coded for retrieval and analysis. The data collected from the key informants and from the focus groups was based on participants’ critical forms and sources of support that facilitate adherence and the social and material barriers that they believe can impede adherence. | The patients and health care workers identified individuals-usually a mother, daughter, sister, brother, or partner-who were confidantes and had moral authority with them. These individuals command respect, and patients allow them to influence health-related decision making, both of which are necessary if they are to be effective treatment supporters. Barriers to adherence identified by study participants include alcohol abuse, stigma related to disclosure of HIV status, and lack of financial resources and food. These are critically important challenges to address if high adherence is to be achieved in this setting. In addition, our results suggest that interventions tailored to treatment supporter characteristics and relationship factors may be effective in influencing patients' antiretroviral therapy adherence | Effective adherence strategy |
| Family support, medication adherence and glycaemic control among ambulatory type 2 diabetic Nigerians in a primary care clinic in Eastern Nigeria | Iloh et al., 2018 | Journal article | from April 2011 to December 2011 | Nigeria | Type 2 diabetic Nigerians who were on treatment for at least 3 months at the primary care clinic | Primary care clinic of a tertiary hospital in Nigeria | Descriptive study | 120 | The role of family support in medication adherence and glycaemic control among ambulatory Type 2 diabetic patients in a primary care clinic in Nigeria. Family support and medication adherence were assessed in the previous 3 months and 1 month preceding the study using multi‑dimensional Scale of Perceived Social Support and interviewer‑administered questionnaire on self‑administered and reported therapy (SAT), respectively. Glycaemic control was assessed in the previous 1 month. | Range: 27-81 years | Females (62.5%) | DM | Medication adherence was assessed by the use of pretested, interviewer administered questionnaire on 30 days self-administered and reported therapy (SAT). Patients were seen at the recruitment visit and at the end of the study visit. At the end of study visit, the adherence section of the data collection tool was administered. The details of the information collected had been explained in specific details in the previous study by the authors. Grading of adherence was done using an ordinal scoring of 0–4 points designed by the researchers from literature review as follows: all times = 4 points, most times = 3 points, sometimes = 2 points, rarely = 1 point, and never = 0 point. | The age of the participants ranged from 27 to 81 years, and there were 37.5% males and 62.5% females with sex ratio of 1:1.7. Family support, medication adherence, and glycaemic control rates were 77.5%, 72.5%, and 61.7%, respectively. Family support was significantly associated with elderly age (0.041), medication adherence (P = 0.038), and glycaemic control (P = 0.027). The most significant demographic predictor of family support was elderly age (odds ratio = 4.30 [2.06–5.15]; P = 0.015). The elderly patients with Type 2 diabetes were four times more likely to have family support compared to their counterparts who were <60 years. | Effective adherence strategy |
| Role of Family support in medication adherence in Type 2 Diabetes Mellitus patients at an outpatient setting in Nigeria: A prospective cohort study. Affiliation: 1St. Nicholas Hospital, Lagos | Adedigba et al., 2019 | Journal article | Between January and April 2016 | Nigeria | Patients with type 2 diabetes mellitus | Outpatient Department of Bingham University Teaching Hospital, Jos, Nigeria | Prospective cohort study | 132 | The influence of family support on medication adherence by comparing medication adherence among patients with good and poor family support using a prospective cohort design. T2DM patients were recruited by systematic random sampling and allocated to two groups based on family support scores. | Mean age: 60.6 (SD±11.3). For the good family support group: 58.8 (SD±10.6) years and in the poor family support group: 62.1 (SD±12.0) | Female: 80 participants Male: 52 participants | DM | Medication adherence scores were among the study participants comparing medication adherence scores at baseline, 4 weeks, and at eight weeks | Medication adherence scores were generally low (5.54±1.7) among the study participants. Respondents had comparable medication adherence scores at baseline (p =0.39) and 4 weeks (p =0.75), but the difference was significant at eight weeks (p =0.01). Multiple logistic regression showed that good family support (OR 2.042; 95% CI 1.219-3.420; p =0.007), age group 45 to 54 years and age group 55-64 years (OR 3.084; 95% CI 1.113-8.543; p =0.03) were significant predictors of good medication adherence. Good family support is a significant predictor of good medication adherence among type 2 diabetes mellitus patients. | Effective adherence strategy |
| EFFECTS OF TWO HEALTH EDUCATION INTERVENTIONS ON ADHERENCE TO ANTIHYPERTENSIVE MEDICATION AND ON BLOOD PRESSURE IN SELECTED TERTIARY HEALTH FACILITIES IN SOUTHWESTERN NIGERIA | Atulomah, 2014 | Thesis | 2013 | Nigeria | Hypertensive patients | Patients receiving care in hypertensive clinics in three tertiary health institutions. Intervention 1 (Olabisi Onabanjo University Teaching Hospital, Sagamu), Intervention 2 (Lagos University Teaching Hospital, Lagos) and Control (University College Hospital, Ibadan) | Quasi-experimental | 180 | Patient Education and Counselling (Intervention 1) and Patient Education and Counselling with Family-Support (Intervention 2) were implemented for four weeks. | Intervention 1; 52.1 (SD±6.5), Intervention 2; 51.3 (SD±7.2) and control group 50.8 (SD±6.2) | Intervention 1, Intervention 2 and control group sexes; Females (28.3%; 41.7%; 38.3%) | HTN | Adherence: Self-Reported Medication Adherence (SRMA), Pill-Count (PC), Appointment Keeping (AK) and Blood Pressure (BP) measurement constituted the outcome variables in this study. SRMA were operationalized in the questionnaire and asked questions about “frequency of forgetting to take prescribed medications”, “frequency of deciding not to take medications for the treatment of hypertension”, “too busy to take medication” and “frequency of getting refill prescription when medications runs out”. Similarly, three questionnaire items were used to measure AK: “frequency of forgetting to go for an appointment”, “too busy to meet scheduled appointment with healthcare giver”, with reversed coding on “frequency of meeting scheduled appointment”. Blood pressure was measured using a mercury sphygmomanometer calibrated in mm of mercury (Hg). Pill-Count was performed by counting pills that were not consumed and subtracting this from total pills prescribed to derive pills consumed during a designated period of 14 days. | At baseline, there were no significant differences in outcome measures among the three groups in respect of primary outcomes of SRMA and secondary outcomes of SBP values respectively. At 13th week follow-up, Intervention 2 demonstrated significantly higher scores in respect of SRMA, PC, AK with SBP reduction from compared with Intervention 1 with SRMA and a corresponding SBP reduction. The values of SRMA, PC and AK in control were 9.6±1.0, 59.6±0.6 and 5.8±1.0 respectively with SBP reduction. Furthermore, the magnitude of changes between outcome measures at 13th week follow-up and their respective baseline values for the three groups. Intervention 2 results were significantly higher | Non-effective adherence strategy |
| Effect of health literacy on medication adherence among diabetic patients. | Olorunfemi, 2018 | Journal article | Not specified | Nigeria | Diabetic patients | University of Benin teaching hospital, state hospital, and Faith Mediplex hospital Benin-city, Edo state, Nigeria. | Correlational research design | 180 | The correlation between health literacy and medication adherence among Diabetic patients. The participants were asked to fill the research instrument on 1) Morisky Medication Adherence test to find out the level of medication adherence among diabetic patients, 2) Demographic data to determine personal information of the participants and 3) Functional Health Literacy in Adults (TOHFLA) test to determine the level of acquiring diabetes-related information among diabetes patients in the identified hospitals. | Mean age was 57.18 (SD±14.15), median= 60, mode =75 | 80 (44.4%) male and 100 (55.6%) female | DM | A correlation between health literacy and medication adherence among diabetic patients was explored. The instrument used was Morisky Medication Adherence Scale (MMAS-8) and Short Functional Health Literacy in Adults Test (STOHFLA). Patients with medication adherence of score zero (0) were rated as higher adherence, 1-2 score of medication adherence were rated as average adherence and 3-8 score of medication adherence were rated as Poor adherence, while health literacy score of 1–16 were taken as inadequate and score of 17 and above were taken as Adequate health literacy. | The findings showed that 100 (55.6%) of the participants had low adherence level, 70(38.9%) had medium adherence level and 10(5.6%) of the participants had higher adherence level. It was also found that 33.3% of the participants had adequate health literacy and 66.7% of the participants had inadequate health literacy. The correlation showed that health literacy with p- value of 0.05 is statistically significant to medication adherence. | Effective adherence strategy |
| Role of social support on adherence to antiretroviral therapy among patients attending AMPATH clinic at Moi Teaching and Referral Hospital, Eldoret, Kenya | Kaguiri, 2014 | Thesis | Data was collected between January and March 2010 | Kenya | HIV positive adult patients who are on ART | Health Care (AMPATH) clinic-Moi Teaching and Referral Hospital (MTRH) in Eldoret | Cross sectional study | 108 | Social support was identified as a intervention to improve adherence. This study sought to estimate proportions of patients reporting optimum adherence to ARVs, to identify the types of social support being utilized by AMPATH patients and to determine the association between social support and adherence to ARVs | 32 (IQR 28-39) | Males (36.1 %) | HIV | Adherence to HAART was measured by self-report using structured interview questions. The Adult AIDS Clinical Trials Group (AACTG) adherence instrument was used and consists of nine questions that assess adherence. The instrument also assesses reasons for non-adherence. Non-adherence was defined as having missed at least one dose during the past 4 days. Adherence to scheduling was measured by the question "Most anti-HIV medications need to be taken on a schedule, such as '2 times a day' or '3 times a day' or 'every 8 hours.' | Optimum adherence was reported by 219(73.2%) of the participants. Those aged 25- 34 years were more likely to adhere compared to those aged below 25 years (Adjusted OR=3.36, 95% CI: 1.44 – 7.81, P = 0.005). Females were more likely to adhere (Adjusted OR=2.80, 95% CI: 1.45 – 5.38, P = 0.002). Most of the participants (184, 61.5%) reported to be members of support groups, 190(63.5%) reported to have disclosed their HIV status to their sexual partners and advice from health care providers was reported by all the participants. Reporting optimum adherence was positively associated with disclosure of HIV status to sexual partner (Adjusted OR= 2.43, 95% CI: 1.37 – 4.29 P value= 0.002), belonging to a support group (Adjusted OR=2.68, 95% CI: 1.51 – 4.76, P value = 0.001) and perceived support from children (Adjusted OR=2.90, 95% CI: 1.29 – 6.53, P value = 0.01). | Effective adherence strategy |
| Perceived family support and factors influencing medication adherence among hypertensive patients attending a Nigerian tertiary hospital | Olowookere et al., 2018 | Journal article | Not specified | Nigeria | Hypertensive patients | Medical Outpatient Clinic of Federal Medical Centre Owo, Ondo State, Nigeria. | Descriptive cross-sectional study. | 420 | This descriptive cross-sectional study assessed perceived family support and other factors that determine medication adherence among hypertensive patients in a tertiary hospital. | 60.6 (SD±11.7) years, range: 21-85 years. | Females: 214 (51%) | HTN | The relationship between family support and adherence to drug treatment amongst hypertensive outpatients was explored. Degree of adherence by individual patients was estimated manually by means of patient self-report. The degree of adherence from patient self-report was estimated using the number of pills prescribed minus the number of pills missed over number of pills prescribed. From the formula, level of adherence by individual patients was categorized into those with less than 80% adherence and those with equal to or more than 80% adherence. Adherent patients were defined in this study as individuals with at least 80% adherence level. | Most respondents were Yoruba (86.2%), married (76.7%), and had primary education (27.6%). Most (61%) were adherent to antihypertensive therapy. Common reasons for poor adherence include belief of cure (43%), high cost of treatment (33%), and the experiencing of side effects (27%). Patients with good family support had better adherence compared to those with poor family support (P < 0.05). | Effective adherence strategy |
| "They just come, pick and go." The Acceptability of Integrated Medication Adherence Clubs for HIV and Non Communicable Disease (NCD) Patients in Kibera, Kenya | Venables et al., 2016 | Journal article | Jan-March 2015 | Kenya | HIV and Non-Communicable Disease (NCD) patients | Kibera South Clinic, Kenya | Qualitative study | 106 | We conducted a qualitative research study to assess patient and health-care worker perceptions and experiences of MACs in the urban informal settlement of Kibera, Kenya. A total of 106 patients (with HIV and/or other NCDs) and health-care workers were purposively sampled and included in the study. Ten focus groups and 19 in-depth interviews were conducted, and 15 sessions of participant observation were carried out at the clinic where the MACs took place. Thematic data analysis was conducted using NVivo software, and coding focussed on people’s experiences of MACs, the challenges they faced and their perceptions about models of care for chronic conditions. | Average age: 48 years | Female (64%) | HIV AND NCDs | Key themes around the acceptability and perceptions of MACs were compared across focus group discussions, interviews and participant observation. All transcripts and fieldnotes were coded through an iterative process that involved firstly coding the text into broad themes, then grouping these together and developing sub-themes. Emphasis was placed on comparing the perspectives of HIV positive and NCD patients, as well as MAC-members and non-MAC members, to see if they had differing experiences of MACs. | MACs were considered acceptable to patients and health-care workers because they saved time, prevented unnecessary queues in the clinic and provided people with health education and group support whilst they collected their medication. Some patients and health-care workers felt that MACs reduced stigma for HIV positive patients by treating HIV as any other chronic condition. Staff and patients reported challenges recruiting patients into MACs, including patients not fully understanding the eligibility criteria for the clubs. There were also some practical challenges during the implementation of the clubs, but MACs have shown that it is possible to learn from ART provision and enable stable HIV and NCD patients to collect chronic medication together in a group. | Effectiveness of adherence strategy is unclear |
| The Influence of Family/Social Support on Adherence to Diabetic Therapy | Affusim, 2018 | Journal article | Not specified | Nigeria | Adults diabetic patients | Outpatient clinic of a tertiary health facility in a rural area of Edo state, Nigeria | Descriptive cross-sectional study. | 158 | Family/Social Support: The Multidimensional Scale of Perceived Social Support (MDSPSS) was used to assess the level of social support the respondents receive from family and friends. The Morisky Medication Adherence Scale-8 (MMAS) was used to assess the level of adherence to medication. Data were collated and analyzed using the IBM-SPSS version 21.0 | 56 (SD±11.5) years, with most of the respondents (63.9%) aged 46-65 years | Females (53.8%) | DM | Adherence to medication was assessed using the Morisky Medication Adherence scale-8 (MMAS). This tool is an 8-item medication adherence questionnaire. Each item is scored either 1 (for Yes answer) or 0 (for No answer). The total score ranges from 0 to 8. Those with aggregate score of 0 were regarded as having high adherence, those with 1-2 score were regarded as having moderate adherence and those with 3-8 score were regarded as having low adherence. The level of support that the participants receive from their family, friends and special persons in their lives was assessed using the Multidimensional Scale of Perceived Social Support (MDSPSS). This tool contains 12 items and each is scored between 1 and 7. The total score is derived from dividing the sum across all 12 items by 12. The aggregate score is thus from 1 to 7. Those with aggregate score of 1-2 were regarded as having low support, those with aggregate score of 3-5 were regarded as having moderate support, while those who had an aggregate score of 5.1-7 were regarded as having high support. | About 48.8% were found to have high social support, 40.5% had moderate support, while 10.8% had low social support. Most of the respondents (70.9%) had low adherence, 19.6% had moderate adherence, while 9.5% had high adherence. There was the significant association between social support and medication adherence, social support, and clinic attendance, social support and BMI, but no significant association between social support and blood sugar level. Since social support can predict health-promoting behavior, it can also predict self-care behavior of patients with DM. Therefore, getting the family members, especially the spouse, involved in self-care behavior can be of significant importance in providing health care to patients with diabetes. | Effective adherence strategy |
| The Relationship between Family Support and Medication Adherence among Hypertensive Patients in Kenya | Xiong, 2018 | Master thesis | unknown | Kenya | Hypertensive patients | Three healthcare facilities in Nairobi, Kenya | Mixed-method cross-sectional study | 104 | Family support and medication adherence among hypertensive patients in Kenya. Fisher’s exact test and Chi-square test were used to compare the level of medication adherence and family support among different subgroups of patients; bivariate logistic regression was used to determine the predictors of medication adherence; and multiple logistic regression was used to examine the independent association between family support/function and medication adherence. Grounded theory was used to guide the thematic analysis of the qualitative data. | 56.61 (SD±11.70) | Female (n = 71, 68.27%) | HTN | The medication adherence measurements not specified. Descriptive statistics were used to describe the patient profiles; Test of associations were used to compare the level of medication adherence and family support among different subgroups of patients. Logistic regression was used to determine the predictors of medication adherence; and to examine the independent association between family support/function and medication adherence. Grounded theory was used to guide the thematic analysis of the qualitative data. | The overall control rate of HTN among the patients was low, with only 33.98% of them under control. The percentage of highly adherent patients determined by the Morisky Green Scale was 55.77% and was 26.92% as determined by the Hill-Bone Scale. Based on the Perceived Social Support from Family Scale, most of the patients (82.69%) reported strong family support. The majority of patients (77.88%) were determined to have “functional” families by the Family Function APGAR Scale, and 22.12% had dysfunctional families. Both the bivariate logistic regression and multiple logistic regression generated non-significant results for the association between family support/function and medication adherence using either scale. | Effectiveness of adherence strategy is unclear |
| Cash vs. food assistance to improve adherence to antiretroviral therapy among HIV-infected adults in Tanzania | McCoy et al., 2017 | Journal article | December 2013-July 2015. Participants were prospectively followed for 12 months. | Shinyanga, Tanzania | HIV positive patients who are on ART experiencing food insecurity | Three facilities (two hospitals and one peri-urban clinic) in Shinyanga, a resource-limited region in Tanzania | Randomized controlled trial | 805 | Nutrition assessment and counselling (NAC) and cash transfers (∼$11/month, n = 347), NAC and food baskets (n = 345), and NAC-only (comparison group, n = 113). Cash or food was provided for 6 or less consecutive months, conditional on visit attendance. | 35 (IQR 29–43) | Female 509 (64%) | HIV | The primary outcome was medication possession ratio (MPR ≥ 95%) at 6 months. Secondary outcomes were appointment attendance and loss to follow-up (LTFU) at 6 and 12 months. | The primary intent-to-treat analysis included 800 participants. Achievement of MPR ≥ 95% at 6 months was higher in the NAC + cash group compared with NAC-only (85.0 vs. 63.4%), a 21.6 percentage point difference [95% confidence interval (CI): 9.8, 33.4, P < 0.01]. MPR ≥ 95% was also significantly higher in the NAC + food group vs. NAC-only (difference = 15.8, 95% CI: 3.8, 27.9, P < 0.01). When directly compared, MPR ≥ 95% was similar in the NAC + cash and NAC + food groups (difference = 5.7, 95% CI: -1.2, 12.7, P = 0.15). Compared with NAC-only, appointment attendance and LTFU were significantly higher in both the NAC + cash and NAC + food groups at 6 months. At 12 months, the effect of NAC + cash, but not NAC + food, on MPR ≥ 95% and retention was sustained. | Effective adherence strategy |
| Adapting an adherence support workers intervention: engaging traditional healers as adherence partners for persons enrolled in HIV care and treatment in rural Mozambique. | Audet et al., 2017 | Journal article | March to July 2016 | Mozambique | Traditional healers and HIV positive persons enrolled in HIV care | Rural Mozambique | Qualitative study | 180 | Adherence support workers intervention: Researchers chose the Adherence Support Workers program, developed by FHI 360, to provide an optimal foundation for our intervention. The Adherence Support Workers program includes three primary activities: (1) education and psychosocial support to PLHIV initiating/continuing ART; (2) referrals to specialized clinics as needed; and (3) participation of support workers as members of the ART clinical team. | 40 (IQR 28-51) | Female 71 (66%) | HIV | Framework analysis was used to identify main themes from our FGDs about the drivers, core facilitators, and barriers to acceptability of the traditional healer-based intervention approach. Four code maps were developed to categorize data: social, structural, and informational drivers, facilitators, and barriers to acceptability of healers as adherence partners; educational and counselling strategies that healers could use to encourage retention in care and adherence to ART (e.g., partner counselling); the role that healers could play in ensuring that respectful care is provided by clinicians; and recommendations for the development of a “best practice” intervention strategy, including the necessary information, motivation (for healers and PLHIV), and appropriate behavioral considerations healers would be required to follow. | Traditional healers were an acceptable group of community health workers to assist with patient adherence and retention. Traditional healers, clinicians, and interested community members suggested novel strategies to tailor the adherence support worker intervention, revealing a local culture of HIV denialism, aversion to the health system, and dislike of healthcare providers, as well as a preference for traditional treatments. Proposed changes to the intervention included modifications to the training language and topics, expanded community-based activities to support acceptability of an HIV diagnosis and to facilitate partner disclosure, and accompaniment to the health facility by healers to encourage delivery of respectful clinical care. PLHIV, healers, and clinicians deemed the intervention socially acceptable during focus groups. We subsequently recruited 180 newly diagnosed HIV-infected patients into the program: 170 (94%) accepted. | Effectiveness of adherence strategy is unclear |
| The effectiveness of social resource intervention to promote adherence to HIV medication in a multidisciplinary care setting in Kenya. | Kamau et al., 2012 | Journal article | Not specified | Kenya | Small-scale farmers, and petty trade or casual labourers who worked in tea, coffee and flower plantations and were HIV positive patients who were prescribed ART | Nine satellite centres under the auspice of the Nazareth Hospital in Kenya | Cross-sectional design | 354 | The comprehensive HIV treatment and care programme (intervention) encompasses treatment preparation sessions, individual and group counselling, assignment of treatment partners to new patients at the start of HIV treatment, and the provision of the necessary social resources. | 18-30=21.8%, 31-40=43.5%, 41-50=26.8%, 51-64=7.9% | Female (71.4%) | HIV | Adherence to HIV medication was defined as the correct consumption of all doses and pills at the correct timings, and following all other instructions provided for in the previous four days. A four-day self-assessment period was selected because patients’ recall for self-reported adherence is better for shorter periods, when compared with longer periods such as 28 days or more. Any patient who missed any dose, pill or failed to follow proper timing guidelines or other instructions was categorized as non-adherent. The measure also included 13 items to determine patients’ reasons for failing to follow the medication schedule correctly. | Binomial logistics were used to test the relationships between social support and its dimensions with adherence to HIV medication. Composite social support was predictive of adherence to HIV medication (P ,0.05). Among the four dimensions of support, material and emotional support were the strongest predictors. | Effective adherence strategy |
| Individualised Motivational Counselling to Enhance Adherence to Antiretroviral Therapy is not Superior to Didactic Counselling in South African Patients: Findings of the CAPRISA 058 Randomised Controlled Trial. | Van Loggerenberg et al., 2015 | Journal article | Between August 2007 to February 2009 | South Africa | HIV positive adults eligible to initiate ART (at the time, a CD4 T-cell count of 350 or less). | CAPRISA eThekwini HIV-TB clinic, in Durban, South Africa. | Parallel group (1:1 ratio), open label randomised controlled trial | 297 | On determination of ART eligibility, all patients received two pre-initiation 20–45-minute didactic counselling sessions as standard at the clinic. The didactic arm participants received the final 20–45-minute didactic counselling session only. The motivational counselling arm participants instead received the first 30–40-minute individualised motivational counselling session, and then the additional four individualised motivational counselling sessions at intervals up to six months after initiation of therapy. All participants were followed up to at least nine months on treatment. The standard didactic counselling consisted of three sessions. The first session covered stigma and discrimination, nutrition and taking control of the treatment regimen. The second session focused on HIV pathogenesis, routes of infection, HIV testing techniques, and a basic introduction to ART. The final session focused on information relating to ART, the drug regimen, the importance of adherence, side effects, and the importance of monitoring and the development of resistance. | mean 35.8 years | Male (43.1%) | HIV | The primary outcome was the proportion of participants with suppressed viral load (plasma viral load of <400 copies/ml, HIV-1 RNA-PCR – Roche Diagnostics) at nine months (range of eight to 10 months) post-initiation of ART. A secondary outcome looked at adherence by pill count over the previous inter-visit period at six months post-initiation of therapy. Pill count was expressed as a percentage of the pills taken as prescribed over the study visit interval (usually the previous 28 days), by assuming that all non-returned pills had been taken over the study visit interval. These data were verified from the clinic records based on previous study visit and drugs dispensed by the pharmacy. This measure was recorded at 6 months, as this was part of the counselling intervention study follow-up. Suppressed viral load at 12 months was assessed as an additional secondary outcome. | We randomised 297 HIV-positive ART-naïve patients in Durban, South Africa, to receive either didactic counselling, prior to ART initiation (n=150), or an intensive motivational adherence intervention after initiating ART (n=147). Study arms were similar for age (mean 35.8 years), sex (43.1% male), CD4+ cell count (median 121.5 cells/μl) and viral load (median 119 000 copies/ml). Virologic suppression at nine months was achieved in 89.8% of didactic and 87.9% of motivational counselling participants (risk ratio [RR] 0.98, 95% confidence interval [CI] 0.90-1.07, p=0.62). 82.9% of didactic and 79.5% of motivational counselling participants achieved >95% adherence by pill count at six months (RR 0.96, 95%CI 0.85-1.09, p=0.51). Participants receiving intensive motivational counselling did not achieve higher treatment adherence or virological suppression than those receiving routinely provideddidactic adherence counselling. These data are reassuring that less resource intensive didactic counselling was adequate for excellent treatment outcomes in this setting. | Non-effective adherence strategy |
| How community ART delivery may improve HIV treatment outcomes: Qualitative inquiry into mechanisms of effect in a randomized trial of community-based ART initiation, monitoring and re-supply (DO ART) in South Africa and Uganda | Gilbert et al., 2021 | Journal article | 2016-2019 | western Uganda and KwaZulu-Natal South Africa | HIV positive patients who are on ART | Two peri-urban sites in KwaZulu-Natal, South Africa, and one site in rural Sheema District, in southwest Uganda. | A qualitative study with a three-arm randomized trial of community ART initiation. | 150 | The Delivery Optimization for Antiretroviral Therapy (DO ART) Study offered ART initiation, monitoring and refills in communities to clinically stable adults living with HIV and not taking ART at the time of enrolment or within the previous 3 months. (1) community-based ART initiation, monitoring and re-supply via mobile vans; (2) clinic-based ART initiation with community-based monitoring and re-supply (“hybrid” services); and (3) clinic-based ART (standard of care). | 56% between 30 and 49 years | Female (49%) | HIV | Individual participants’ experiences of receiving ART in the community were summarized and entered into a matrix. The matrix both preserved the coherence of individual experiences and helped to identify patterns that cut across the data, for a thematic analytic approach. Thematic concepts identified through the matrix were elaborated through coded data. Coded data corresponding to these concepts were retrieved, revised and added to the thematic concepts. The revised concepts were then labelled, described and illustrated to form descriptive categories. By employing this combined thematic and content analytic approach, a set of categories representing a variety of potential mechanisms was set based on the perspectives and priorities of DO ART Study participants. | The analysis yielded four potential mechanisms drawn from qualitative data representing the perspectives and priorities of DO ART participants. Empowering participants to schedule, re-schedule and select the locations of community-based visits via easy phone contact with clinical staff is characterized as flexibility. Integration refers to combining the components of clinic-based visits into single interaction with a healthcare provider. Providers” willingness to talk at length with participants during visits, addressing non-HIV as well as HIV-related concerns, is termed “a slower pace”. Finally, increased efficiency denotes the time savings and increased income-generating opportunities for participants brought about by delivering services in the community. | Effectiveness of adherence strategy is unclear |
| The Treatment Ambassador Program: A Highly Acceptable and Feasible Community-Based Peer Intervention for South Africans Living with HIV Who Delay or Discontinue Antiretroviral Therapy | Katz et al., 2021 | Journal article | 2017 | South Africa in Gugulethu township | HIV positive people who are not on ART | Community based in Gugulethu township | Randomized controlled trial of an intervention | 84 | Treatment ambassador program (TAP). Core intervention components included one-on-one client-cantered counselling sessions and patient navigation. TAP was hypothesized to work through several mechanisms and levels as framed by the TTI: (1) individual-level factors, including attitudes and beliefs about treatment, by building the knowledge base and trust of treatment for participants, while promoting self-efficacy and effective coping strategies; (2) social-level factors through social interactive processes that address HIV-related stigma and the need for disclosure; and (3) structural-level factors through facilitating engagement with clinic providers. the full intervention consisted of eight sessions over 8–14 weeks for people living with HIV who had not initiated treatment within 6 months of testing or had previously initiated ART but been off treatment for over 6 months. | median age: 43 years | Female (77%) | HIV | Survey measures were administered at baseline and follow-up to assess psychosocial characteristics, as well as sociodemographic and medical characteristics potentially related to behavior at baseline and at follow-up in both arms of the study to evaluate the moderators and mediators of intervention effects. The Theory of Triadic Influence (TTI) states that health related behaviours are shaped by individual-, social-, and structural-level factors and thus, the survey attempted to analyze barriers at these three levels. | TAP was highly feasible (90% completion), with peer counsellors demonstrating good intervention fidelity. Post-intervention interviews showed high acceptability of TAP and counsellors, who supported autonomy, assisted with clinical navigation, and provided psychosocial support. Intention-to-treat analyses indicated increased ART initiation by 3 months in the intervention vs. control arm (12.2% [5/41] vs. 2.3% [1/43], Fisher exact p-value = 0.105; Cohen’s h = 0.41). Among those previously on ART (off for > 6 months), 33.3% initiated ART by 3 months in the intervention vs. 14.3% in the control arm (Cohen’s h = 0.45). Results suggest that TAP was highly acceptable and feasible among PWH not on ART. | Effective adherence strategy |
| Those People Motivate and Inspire Me to Take My Treatment. Peer Support for Adolescents Living With HIV in Cape Town, South Africa | Rencken et al., 2021 | Journal article | 2016 and 2017 | Cape Town, South Africa | Adolescents living with HIV who are on ART | A large public hospital in Cape Town, South Africa | Qualitative study with in-depth interviews | 35 | ALHIV peer support group | Median age: 15 years (range:12-19 years) | 19 female and 16 male | HIV | The analysis and outcome for this paper is based on the codes associated with the domain of ‘barriers to and facilitators of adherence.’ Thematic analysis using an iterative process detailed the codes and nodes through an inductive process (to finalize the codebook). Overall, the study explored (1) barriers and facilitators of adherence, including stigma, fears about HIV disclosure, and access to treatment; (2) psychosocial support and the role of the peer groups; and (3) different aspects of conditional economic incentives, including acceptability, likely influences on behavior, and the preferred format. | Three themes emerged: (1) peer support encouraged adherence to ART, (2) serostatus disclosure outside the family was perceived as difficult, and (3) the peer support group fostered fundamental and meaningful peer relationships for ALHIV. Caregivers felt peer support groups increased self-acceptance and adherence for ALHIV across 3 domains: (1) as motivation for families and adolescents, (2) to increase adolescent independence and maturity, and (3) to help adolescents accept their HIV status and live successfully with HIV. These data highlight the importance of psychosocial support groups for ALHIV and caregivers, illustrating the benefits of a safe space with trusted relationships and open communication. | Effective adherence strategy |
| Out-of-Facility Multimonth Dispensing of Antiretroviral Treatment: A Pooled Analysis Using Individual Patient Data From Cluster-Randomized Trials in Southern Africa | Lopes et al., 2021 | Journal article | 2017 and 2018 | Zimbabwe and Lesotho | HIV positive patients who are on first line ART | Study facilities (n = 60) were public health facilities in 8 high HIV-prevalence districts of Zimbabwe and Lesotho in Southern Africa. | A meta analysis using individual-level data from the 2 cluster randomized trials. | 10136 | **Control arm (3MF)**: Participants received facility-based standard-of care ART and clinical consultations at three-monthly intervals. **Intervention arm 1 (3MC):** Participants received ART at three-monthly intervals within community ART groups (CAGs) with annual facility visits and clinical consultations. **Intervention arm 2 (6MC):** Participants received ART at 6-monthly intervals within CAGs (Zimbabwe) or at community distribution points (Lesotho) with annual facility visits and clinical consultations. | Median: 3MF: 44.1 (IQR 36.4–53.8), 3MC 47,4 (IQR 40.1–57.0), 6MC 43.0 (IQR 35.2–52.6) | 3MF: 66,4% female, 3MC: 72,6% female, 6MC: 69,0% female | HIV | The primary outcome was the proportion of enrolled participants retained in ART care after 12 months by intention-to-treat including participants in each arm as per baseline allocation. Secondary outcomes were the proportions achieving VS after 12 months, retention in the study arm (retention in the randomized strategy), all-cause mortality after 12 months, incidence of unscheduled facility visits between months 0–12 after enrolment and participant attrition from ART care using time-to-event analyses until 18 months in Zimbabwe (follow-up data collection did not continue to 18 months in Lesotho). | Ten thousand one hundred thirty-six participants were included, 3817 (37.7%), 2893 (28.5%) and 3426 (33.8%) in arms 3MF, 3MC and 6MC, respectively. After 12 months, retention was non-inferior for 3MC (95.7%) vs. 3MF (95.0%) {adjusted risk difference (aRD) = 0.3 [95% confidence interval (CI): −0.8 to 1.4]}; and 6MC (95.1%) vs. 3MF [aRD = −0.2 (95% CI: −1.4 to 1.0)]. Retention was greater amongst intervention arm participants in CAGs versus 6MC participants not in CAGs, aRD = 1.5% (95% CI: 0.2% to 2.9%). Viral suppression was excellent (≥98%) and unscheduled facility visits were not increased in the intervention arms. | Effective adherence strategy |
| The Money, It's OK but It's not OK: Patients' and Providers' Perceptions of the Acceptability of Cash Incentives for HIV Treatment Initiation in Cape Town, South Africa | Swartz et al., 2021 | Journal article | 2015-2016 | Cape Town, South Africa | HIV positive patients who are on ART | Mobile health clinic in Cape Town | Qualitative study with in-depth interviews | 64 | The intervention group received the standard of care plus a voucher that could be exchanged for R300 (~ $25USD) cash if ART was started within three months. After ART initiation, participants in the intervention group met with the study staff to verify ART initiation and receive the incentive. | Not specified | Not specified | HIV | No clear outcome measure documented. Data analysis adopted a combination of inductive and deductive approaches, following the specific elements of the Sekhon et al framework that guided this analysis, namely affective attitudes and ethicality. Potential links between emergent themes and the dimensions of affective attitudes and ethicality of incentives were explored. Data from the patient and provider perspectives enabled the comparisons and contrasting perspectives, thereby contributing to a richer and deeper understanding of attitudes and perceptions of incentives. | Drawing on in-depth interviews with patients and health care workers (HCWs), we find that, despite the perception that cash incentives are effective in promoting ART initiation, significant ambivalence surrounds the acceptability of such incentives. The receipt of a financial incentive was highly moralized, and fraught with challenges. Increasing the acceptability of cash incentives through careful design and delivery of interventions is central to the potential of this type of intervention for improving outcomes along the HIV care continuum. | Effective adherence strategy |
| Community-based differentiated service delivery models incorporating multi-month dispensing of antiretroviral treatment for newly stable people living with HIV receiving single annual clinical visits: a pooled analysis of two cluster-randomized trials in southern Africa | Fatti et al., 2021 | Journal article | 2017-2019 | Zimbabwe and Lesotho | HIV positive patients who are on ART | Community-based differentiated service delivery | Cluster-randomized trial | 599 | **Control arm (SoC):** Participants received standard-of-care ART and clinical consultations at three-monthly intervals at facilities. **Intervention arm 1 (3MC):** Participants received ART at three-monthly intervals in community ART groups (CAGs) with annual facility visits and clinical consultations. **Intervention arm 2 (6MC):** Participants received ART at six-monthly intervals in CAGs (Zimbabwe) or community distribution points (Lesotho) with annual facility visits and clinical consultations. | Median age: All participants: 39.8 (IQR 32.8–49.6); SoC: 38,6 (IQR 32.2–48.1); 3MC: 42,6 (IQR 35.7–50.7); 6MC: 39,8 (IQR 32.1–49.6) | SoC: 55,7% female; 3MC: 75% female; 6MC:64,5% female | HIV | The primary outcome was the proportion remaining in ART care 12 months after enrolment by intention-to-treat including participants in each arm as per baseline allocation. Secondary outcomes were proportions achieving viral suppression (VS) after 12 months, and the number of unscheduled facility visits between months 0 and 12. | A total of 599 participants were included; 212 (35.4%), 128 (21.4%) and 259 (43.2%) in SoC, 3MC and 6MC, respectively. Few participants aged <25 years were included (n = 32). After 12 months, 198 (93.4%), 123 (96.1%) and 248 (95.8%) were retained in SoC, 3MC and 6MC, respectively. Retention in 3MC was superior versus SoC, adjusted risk difference (aRD) = 4.6% (95% CI: 0.7%−8.5%). Retention in 6MC was non-inferior versus SoC, aRD = 1.7% (95% CI: −2.5%−5.9%) (prespecified non-inferiority aRD margin −3.25%). VS was similar between arms, 99.3, 98.6 and 98.1% in SoC, 3MC and 6MC, respectively. Adjusted risk ratio's for VS were 0.98 (95% CI: 0.92−1.03) for 3MC versus SoC, and 0.98 (CI: 0.95−1.00) for 6MC versus SoC. Unscheduled clinic visits were not increased in intervention arms: incidence rate ratio = 0.53 (CI: 0.16−1.80) for 3MC versus SoC; and 0.82 (CI: 0.25−2.79) for 6MC versus SoC. | Effective adherence strategy |
| Pharmacist-led medication therapy management of diabetes club patients at a primary healthcare clinic in Cape Town, South Africa: A retrospective and prospective audit | Sonday et al., 2022 | Journal article | 2016 and 2017 | Cape Town, South Africa | Type 2 diabetes mellitus patients | Community day centre (CDC) | Evaluation study design using a case study approach | 104 | Medication Therapy Management (MTM) intervention among stable diabetes club patients. The CDC has a functional ‘club’ system whereby patients with DM who adhere to their medicine regimen and have minimal changes in their clinical status (classified as stable) are referred to the club, to which they return every 6 months for their follow-up appointment. ‘Stable’ diabetes club patients are seen by either the club doctor or a clinical nurse practitioner (CNP) and their appointment dates are recorded in a club register. | 57,7 (SD±9.2), range: 26-80 | Female (67.3%) | DM | An evaluation of the implementation of a pharmacist-led MTM intervention to optimise the management of stable patients with type 2 DM attending a diabetes club at a Cape Town community day centre. | Of 104 patient folders audited, most were for females (n=70; 67.3%). A total of 453 MTPs were identified, averaging four interventions per folder reviewed. The most common MTPs identified were the absence of basic clinical data: body mass index not documented (22.5%) in the folder, no medical indication noted (19.2%), and laboratory tests not requested (18.3%) by clinicians. Prescriber acceptance of the pharmacist’s recommendations was found to be low (26.8%), suggestive of clinical inertia. Aspirin was found to be irrationally prescribed to patients with DM (15.4%). | Effectiveness of adherence strategy is unclear |
| The role of pharmacy personnel in promoting adherence to antiretroviral therapy in the Eastern Cape: communication barriers and breakthroughs | Rattine-Flaherty et al., 2021 | Journal article | 2018 | Eastern cape, South Africa | Pharmacy personnel | Public health clinics | Qualitative study with in-depth interviews | 24 | Pharmacy support | Age not specified | Not specified | HIV | No clear outcome measure documented. An understanding of what role pharmacy personnel believe that they play in the goal of patient adherence: an exploration of the key communication challenges faced by pharmacy staff as they serve patients with HIV and the strategies these health providers use to address personal, cultural and environmental barriers that impede effective patient counselling and limit adherence. | Pharmacy personnel identified three key sets of communication challenges that limited effective patient counselling on antiretroviral therapy. These included environmental barriers presented by clinic design, language barriers between patients and pharmacy personnel, and varying communication styles and education levels of pharmacy staff and patients. Additionally, pharmacy personnel described innovative strategies they use to improve patient-provider communication and address adherence issues. | Effectiveness of adherence strategy is unclear |
| One Pill, Once a Day: Simplified Treatment Regimens and Retention in HIV Care | Bor et al., 2022 | Journal article | 2011 to 2014 | Themba Lethu clinic, Johannesburg, South Africa | HIV positive naive adult patients initiating first-line ART | ART treatment clinic | Quasi experimental regression discontinuity design | 4484 | Fixed-dose combinations (FDCs) combining 3 antiretroviral medications into a single daily pill. | 38,5 (SD±9.9) | Male (43%) | HIV | Our primary exposure was whether the patient starting ART was prescribed an FDC or multiple-pill regimen (“regimen type”). We classified patients as starting FDC if either source indicated an FDC regimen. We assumed that patients who initiated ART prior to the September 2012 availability of pharmacy data were prescribed multiple pills, since FDCs were not yet available. We used date of ART initiation as the assignment variable in the RDD, with patients starting on April 1, 2013, or later exposed to the new guidelines. | The share of patients prescribed a single-pill regimen increased by over 40 percentage points between March and April 2013. Initiating treatment after the policy change was associated with 11.7–percentage-points’ higher retention at 12 months (95% confidence interval: −2.2, 29.4). Findings were robust to different measures of retention, different bandwidths, and different statistical models. Patients starting treatment early in HIV infection—a key population in the test-and-treat era—experienced the greatest improvements in retention from single-pill regimens. | Effective adherence strategy |
| Intervention development of a brief messaging intervention for a randomised controlled trial to improve diabetes treatment adherence in sub-Saharan Africa | Leon et al., 2021 | Journal article | 2018 | Lilongwe, Milawi and Cape Town, and Johannesburg South africa | Type 2 diabetes mellitus patients | Public sector health facilities in urban/ peri urban sites | Randomized controlled trial with multiple, qualitative research strategies. |  | SMS text Adherence support for people with type 2 diabetes (StarR2D). The aim of the StAR2D intervention development study was to ensure the final brief (SMS) text-message intervention was theory- and evidence-informed, relevant, and acceptable to the target audiences, and appropriately aligned with the organisation of clinic care at the trial sites. Intervention messages were meant to advise people about the benefits of their diabetes treatment and offer motivation and encouragement around lifestyle and use of medication. | Lilongwe, Malawi: age range 28–78 years), Cape Town, South Africa, age range 47–80 years) (Johannesburg, South Africa, age 42–68 years) | Not specified | DM | The primary focus of this paper is to document a systematic, transparent approach to intervention development in the context of an intervention that was tested experimentally. A secondary, but important focus is on the evidence generated in each phase to illustrate its contribution to shaping the final intervention. | We used a four-phase, iterative approach that first generated primary and secondary evidence on the lived experience of diabetes, diabetes treatment services and mobile-phone use. Second, we designed a type 2 diabetes-specific, brief text-message library, building on our previous hypertension text-message library, as well as drawing on the primary and secondary data from phase one, and on expert opinion. We then mapped the brief text-messages onto behaviour change (COM-B) theoretical constructs. Third, we refined and finalised the newly developed brief text-message library through stakeholder consultation and translated it into three local languages. Finally, we piloted the intervention by pre-testing the automated delivery of the brief text-messages in the trial sites in Malawi and South Africa. The final SMS text Adherence support for people with type 2 diabetes (StAR2D) intervention was tested in a randomised controlled trial in Malawi and South Africa | Effectiveness of adherence strategy is unclear |
| Multimonth dispensing of up to 6 months of antiretroviral therapy in Malawi and Zambia (INTERVAL): a cluster-randomised, non-blinded, non-inferiority trial | Hoffman et al., 2021 | Journal article | 2017 and 2018 | Malawi and Zambia | HIV positive adult patients who are on ART | 30 public health faclities | A pragmatic, cluster-randomised, unblinded, non-inferiority trial | 9118 | Matched clusters were randomly assigned (1:1:1) to receive standard of care ART dispensing (ART typically given every 1–3 months, depending on provider judgment), 3-monthly (ie, 90-day) ART dispensing, or 6-monthly (ie, 180-day) ART dispensing. For all groups, clinical service delivery occurred simultaneously with the dispensing visits. Thus, the only differences between the study groups were the frequency of clinical consultations and the amount of ART given at these visits. | 42·7 years (IQR 36.1–49·9) | Female (66.2%) | HIV | [The primary outcome was retention in care at 12 months, defined as the proportion of patients with less than 60 consecutive days without ART during study follow-up, analysed by intention to treat. Secondary outcomes were viral suppression (defined as <1000 copies per mL in Malawi and <20 copies per mL in Zambia), feasibility and acceptability of dispensing intervals to participants and providers, and cost (per patient achieving the primary outcome in each group).](https://www.sciencedirect.com/topics/medicine-and-dentistry/intention-to-treat-analysis) | Between May 15, 2017, and April 30, 2018, 9118 participants were randomly assigned, of whom 8719 participants (n=3012, standard of care group; n=2726, 3-monthly ART dispensing group; n=2981, 6-monthly ART dispensing group) had primary outcome data available at 12 months and were included in the primary analysis. The median age of participants was 42·7 years (IQR 36·1–49·9) and 5774 (66·2%) of 8719 were women. The primary outcome was met by 2478 (82·3%) of 3012 participants in the standard of care group, 2356 (86·4%) of 2726 participants in the 3-monthly ART dispensing group, and 2729 (91·5%) of 2981 participants in the 6-monthly ART dispensing group. After adjusting for clustering, for retention in care at 12 months, the 6-monthly ART dispensing group was non-inferior to the standard of care group (percentage-point increase 9·1 [95% CI 0·9–17·2]) and to the 3-monthly ART dispensing group (5·0% [1·0–9·1]). | Effective adherence strategy |
| A nurse-led intervention to improve management of virological failure in public sector HIV clinics in Durban, South Africa: A pre- and post-implementation evaluation | Sunpath et al., 2021 | Journal article | 2017 | Durban, South Africa | HIV positive adult patients who are on first-line ART | 3 public health HIV clinics | Controlled before-after observational design | 116 | A health system-strengthening programme to manage VF at each of the three clinics. The programme included the following elements: Assignment of a nurse as the ‘VL champion’ at each clinic to supervise the staff responsible for monitoring all patients with a detectable VL and Development of an SOP for management of VF by clinic staff. Training on the SOP was provided to: (i) a lay counsellor or nurse assigned to adherence counselling; (ii) a nurse and/or doctor assigned to manage the VF clinic; (iii) an administrative clerk for records handling; and (iv) a data clerk to ensure same-day data entry. In brief, VL results were reviewed daily by the VL champion and filed or entered in the patient charts. The lay counsellor also managed completion of the high VL register and was expected to call patients who missed a clinic appointment within a week at any point of the follow-up period. | Pre-intervention: 36 (IQR 23–41), post-intervention: 35 (IQR 30 – 39) | Pre-intervention: 35% female and post-intervention: 25% female | HIV | The primary outcome was appropriate response completed to the repeat VL, specifically a VL <1 000 copies/mL or change to a protease inhibitor-based regimen after a repeat VL >1 000 copies/mL within 6 months of VF. Secondary outcomes included completion of at least one EAC session and completion of a repeat VL within 6 months. | We identified 60 and 56 individuals in the pre-intervention and post-intervention periods, respectively, with VF who met the inclusion criteria. Sociodemographic and clinical characteristics were similar between the periods. Repeat VL testing was completed in 61.7% and 57.8% of individuals in these two groups, respectively. We found no difference in the proportion achieving our primary outcome in the pre- and post-intervention periods: 11/60 (18.3%; 95% confidence interval (CI) 9 - 28) and 15/56 (22.8%; 95% CI 15 - 38), respectively (p=0.28). In multivariable logistic regression models adjusted for potential confounding factors, individuals in the post-intervention period had a non-significant doubling of the odds of achieving the primary outcome (adjusted odds ratio 2.07; 95% CI 0.75 - 5.72). However, there was no difference in the rates of completion of each step along the first-line VF cascade of care. | Non-effective adherence strategy |
| Impact of SMS and peer navigation on retention in HIV care among adults in South Africa: results of a three-arm cluster randomized controlled trial | Steward et al., 2021 | Journal article | 2014 and 2015 | North West Province, South Africa | HIV positive adult patients who are on ART | Clinical sites included five community healht centres and 13 primary health clinics. | Three-arm cluster randomized controlled trial | 752 | 1. **The SMS-only intervention** used an automated messaging system to deliver three kinds of SMS. First, reminders were sent prior to clinic appointments, and every two weeks after missed appointments until a participant returned to care or three months elapsed. Second, brief behavioural messages were sent biweekly to encourage engagement in care, adherence, prevention (e.g. disclosure to sexual partners) and healthy living (e.g. reduced alcohol use). Third, SMS check-in messages were sent biweekly, asking participants to reply, free of charge using a numeric code, to indicate if they were experiencing challenges. 2. **The SMS+PN intervention** used the same automated system to send SMS appointment reminders and biweekly behavioural messages. Additional support was provided by peer navigators, who were PLHIV receiving care at a local facility. Navigators met each client in-person at least once per month and had one phone or SMS check-in mid-month. 3. **Participants at SOC clinics** (Standard of Care) did not receive services beyond standard care. P | Just over half were younger than 35 | SoC: 61,2% Female; SMS-only: 58,1% Female; SMS+PN: 64,2% Female | HIV | The trial outcome, retention in care, was measured at the individual level and defined by ART status. | Between October 2014 and April 2015, we enrolled 752 adult clients recently diagnosed with HIV (SOC: 167; SMS-only: 289; SMS+PN: 296). Individuals in the SMS+PN arm had approximately two more clinic visits over a year than those in other arms (p < 0.01) and were more likely to be retained in care over one year than those in SOC clinics (54% vs. 38%; OR: 1.77, CI: 1.02, 3.10). Differences between SMS+PN and SOC conditions remained significant when restricting analyses to the 628 participants on ART (61% vs. 45% retained; OR: 1.78, CI: 1.08, 2.93). The SMS-only intervention did not improve retention relative to SOC (40% vs. 38%, OR: 1.12, CI: 0.63, 1.98). | Effective adherence strategy |
| Diabetes self-management: a qualitative study on challenges and solutions from the perspective of South African patients and health care providers | Masupe et al., 2022 | Journal article | 2018 | Cape Town, South Africa | Type 2 melltus diabetes and/or hypertensive patients | 2 community health centres | Qualitative study with 8 in-depth interviews and 4 mixed focus groups. | 43 | Self-management support. Self-management is described based on tasks that fall within three categories: medical/behavioural management, role management, and emotional management. The medical/behavioural role entails proper use of medication for disease control and adopting positive health behaviours geared towards slowing down disease progression. Role management defines new long-term partnerships between patients and healthcare professionals where the patient plays a role of a partner, accurately and truthfully reporting any changes in symptoms. The emotional role consists of the patient’s emotional reactions to chronic disease. | Age range was 38–75 years | 8 Males and 35 Females | HTN AND DM | Identify self-management barriers, and solicit solutions for enhancing self-management of T2D/HTN from patients and their healthcare providers | Patients experienced challenges across all three self-management tasks of behavioural/medical management, role management, and emotional management. Main challenges included poor patient self-control towards lifestyle modification, sub-optimal patient-provider and family partnerships, and post-diagnosis grief-reactions by patients. Barriers experienced were stigma, socio-economic and cultural influences, provider-patient communication gaps, disconnect between facility-based services and patients’ lived experiences, and inadequate community care services. Patients suggested empowering community-based solutions to strengthen their disease self-management, including dedicated multidisciplinary diabetes services, counselling services; strengthened family support; patient buddies; patient-led community projects, and advocacy. Providers suggested contextualised communication using audio-visual technologies and patient-centred provider consultations. | Non-effective adherence strategy |
| Impact of Friendship Bench problem-solving therapy on adherence to ART in young people living with HIV in Zimbabwe: A qualitative study",2021 | Ouansafi et al., 2021 | Journal article | 2019 | Harare, zimbabwe | Young people (aged 18-24 years) living with HIV | Primary care clinics | Qualitative study | 10 | The friendship Bench (FB)-an innovative model developed in Zimbabwe to bridge the gap in mental health treatment. Embedded within the City Health Department of Harare, it offers problem-solving therapy delivered on benches in primary care facilities by trained lay health workers (LHWs), elderly women commonly known as community “grandmothers”. FB counselling consists of six sessions generally completed within four to six weeks. LHWs ask questions, encourage clients to “open their minds”, identify a problem and proactively tackle it. Following problem identification and exploration, LHWs guide their clients on an action plan towards a feasible solution. | Age range: 18-24 years | Female (70%) | HIV | Exploration of the experiences of young people living with HIV attending FB, and their perception of how problem-solving therapy impacted their adherence to ART. | Study findings revealed a clear emotional denial towards HIV, particularly for young people infected perinatally, and a resulting low adherence to ART. The study also unpacked the issues of internal stigma and how young people living with perinatally acquired HIV are informed of their HIV status. Participants reported that FB had a critical role in helping them accept their HIV status. Grandmothers’ empathic attitude was key during counselling on adherence to ART, to demystify the disease and treatment, normalize the reality of living with HIV, encourage young people to socialize with peers and free them of guilt. Interviewees unanimously reported improved ART adherence following FB counselling, and many described enhanced health and wellbeing. | Effective adherence strategy |
| Group medical visit and microfinance intervention for patients with diabetes or hypertension in Kenya | Vedanthan et al., 2021 | Journal article | Not specified | Kenya | Mellitus Diabetes or Hypertension patients | Health care facility | Cluster randomized trial | 2890 | **In the UC arm,** participants received AMPATH’s multicomponent chronic disease management care package. The clinical protocol aligned with contemporaneous relevant global and national guidelines, using drugs contained in the Kenyan national formulary, and included both pharmacologic and nonpharmacologic interventions. There was no charge for the clinical encounter. Standard fees for medications were negotiated between AMPATH and the Kenyan Ministry of Health and did not vary across the trial arms, so that price differences would not bias study results. At the time of the study, the Kenyan national health insurance plan did not cover outpatient chronic disease medications. **In the MF arm,** participants received usual multicomponent clinical care and were encouraged to create MF groups that met monthly and were organized and supported by AMPATH. AMPATH’s MF program involves the creation of community savings groups, wherein MF group members mobilize and manage their own savings, provide interest-bearing loans to group members, offer a limited form of financial insurance, and contribute to a social fund used for emergency or welfare issues of group members. No external funds are provided to the MF groups. All MF group participants received loans from the combined group savings. **In the GMV arm,** participants were invited to join a group that met monthly with a community health worker and clinician (either physician or clinical officer) at a location convenient to the community health worker, clinician, and participants. The clinical care package was identical to the multicomponent AMPATH chronic disease management package, but the delivery was in the form of a GMV rather than individual clinician-patient encounter in the health facility. Each GMV began with the measurement of resting BP (all participants) and blood glucose (diabetes patients), the ascertainment of medication regimen for each participant, and extent of medication adherence. Subsequently, the community health worker facilitated a group discussion about a self-care or health education topic chosen by the group. The community health worker had received training in motivational interviewing, chronic disease self-management techniques, and group-based peer support, and encouraged group members to engage in mutual problem solving and sharing of ideas. During this time, the clinician reviewed the BP, glucose, and adherence data to determine a clinical management plan for each individual. After the group discussion ended, each participant had a brief individual consultation with the clinician during which their individual management plan was discussed and finalized. Each GMV lasted for 2h. **In the GMV-MF arm**, the monthly GMV was integrated into the MF groups, wherein each group meeting consisted of an initial MF portion, followed by the GMV. Thus, participants received their clinical care in a GMV as well as participated in MF, as described above. | 60.7 (SD±12.1) | Female: 2020 (69.9%) | HTN AND DM | The primary outcome measure was 1-year absolute mean change in SBP, measured by trained study staff using electronic BP machines and standardized procedures. Key secondary outcome measures included change in DBP and change in 10-year CVD event risk as measured by the QResearch-based QRISK3 score. Other secondary outcomes included BP control (SBP<140 mm Hg and DBP<90 mm Hg), change in total and low-density lipoprotein cholesterol, change in the International Wealth Index (asset-based index of a household’s material well-being, used in LMICs), and change in livestock ownership. | A total of 2,890 individuals (69.9% women) were enrolled (708 UC, 709 MF, 740 GMV, and 733 GMV-MF). Average baseline SBP was 157.5 mm Hg. Mean SBP declined11.4,14.8,14.7, and16.4 mm Hg in UC, MF, GMV, and GMV-MF, respectively. Adjusted estimates and multiplicity-adjusted 98.3% confidence intervals showed that, relative to UC, SBP reduction was 3.9 mm Hg (8.5 to 0.7), 3.3 mm Hg (7.8 to 1.2), and 2.3 mm Hg (7.0 to 2.4) greater in GMV-MF, GMV, and MF, respectively. GMV and GMV-MF tended to benefit women, and MF and GMV-MF tended to benefit poorer individuals. Active participation in GMV-MF was associated with greater benefit. | Effective adherence strategy |
| Social support for self-care: patient strategies for managing diabetes and hypertension in rural Uganda | Tusubira et al., 2021 | Journal article | 2019 | Nakaseke, Uganda | Mellitus Diabetes and/or Hypertension patients | NCD clinics at three health facilities in rural Uganda | Cross-sectional qualitative study | 19 | Self-care practices and social support: Self-care is a patient-driven process involving activities intended to manage symptoms and maintain physiological stability. Self-care includes all actions patients take to attain and maintain good health. Social support may take the form of physical, financial, or psychological help from family, friends, and community members. It may consist either of emotional support (i.e., a confidant) or instrumental support (i.e., tangible and/or physical assistance). | 55 (SD±12) | Female (47%) | HTN AND DM | An exploration of practices and resources for attaining and maintaining health in light of diagnosis with diabetes and/or hypertension. | Nineteen patients participated. Patients said they preferred conventional medicines as their first resort, but often used traditional medicines to mitigate the impact of inconsistent access to prescribed medicines or as a supplement to those medicines. Patients adopted a wide range of vernacular practices to supplement treatment or replace unavailable diagnostic tests, such as tasting urine to gauge blood-sugar level. Finally, patients sought and received both instrumental and emotional support for self-care activities from networks of family and peers. Patients saw their children as their most reliable source of support facilitating self-care, especially as a source of money for medicines, transport and home necessities. | Effectiveness of adherence strategy is unclear |
| Adherence to diabetes self-care management and associated factors among people with diabetes in Gamo Gofa Zone public health hospitals | Agidew et al., 2021 | Journal article | 2018 | Gamo Gofa Zone, Southern Ethiopia | Mellitus Diabetes patients | 6 Public health hospitals | Institutional-based quantitative cross sectional study | 635 | These hospitals have been giving preventive, curative and rehabilitative service for the catchment population including diabetes and other chronic none communicable diseases. | 48,47 (SD±13.86) | Female (49.5%) | DM | Adherence to diabetes self-care management was the dependent variable while socio-demographic characteristics, dietary feeding practice, physical exercise, eye examination practice and foot care practices were some of the independent study variables as indicated in the result section. | The prevalence of good adherence toward diabetes self-care management was 341 (53.7%), (95% confidence interval=46.09, 61.31). Regarding diabetes self-care practices, poor adherence had been detected in blood sugar measurement practice, dietary feeding practice, physical exercise and eye examination practice. The multivariable analysis indicated that government workers [adjusted odds ratio=2.74 (1.03, 7.30)], training on diabetes self-care practice [adjusted odds ratio=3.13 (1.89, 5.16)], diabetes’ association membership [adjusted odds ratio=1.59 (1.01, 2.50)], having personal glucometer at home [adjusted odds ratio=2.70 (1.37, 5.33)], duration of diabetic illness >10 years [adjusted odds ratio=9.59 (3.99, 23.05)] and people with diabetes who were not developing complication [adjusted odds ratio=1.54 (1.01, 2.33)] were significantly associated with good adherence to diabetes self-care management practice. | Effective adherence strategy |
| Family support and medication adherence among adult type 2 diabetes: Any meeting point? | Olagbemide et al., 2021 | Journal article | 2016 | Ido-Ekiti, Nigeria | Type 2 Mellitus Diabetes patients | Department of family medicine at a federal teaching hospital. A tertiary healthcare facility | Cross-sectional hospital-based study | 367 | Self-care practices and social support: Self-care is a patient-driven process involving activities intended to manage symptoms and maintain physiological stability. Self-care includes all actions patients take to attain and maintain good health. Social support may take the form of physical, financial, or psychological help from family, friends, and community members. It may consist either of emotional support (i.e., a confidant) or instrumental support (i.e., tangible and/or physical assistance). | 61,7 (SD± 11.4) | Female (51.4%) | DM | The relationship between family support and medication adherence among adult Type 2 DM (T2DM) attending family medicine clinic of a rural tertiary hospital. | The mean (standard deviation) age of respondents was 61.7 ± 11.4 years. Those with strong family support that achieved medium/high (Morisky Medication Adherence Scale-8 > 6) level of medication adherence (odds ratio [OR] [95% confidence interval (CI)] = 1 6.4 [9.1–29.6], P < 0.001) constituted 69.5% of respondents. Family support was also found to have a direct relationship to glycemic control (FPG < 7.1 mmol/l), 65.7% of those with strong family support achieved good glycemic control, P < 0.001, OR (95% CI) = 17.4 (9.2–37.2). The level of medication adherence was noted to be directly related to glycemic control, 79.4% of those with medium/high medication adherence had good glycemic control, OR (95% CI) = 25.0 (14.4–43.6), P < 0.001. Strong family support leads to higher medication adherence level which resulted into better glycemic control. | Effective adherence strategy |
| EFFECT OF HEALTH EDUCATION INTERVENTION IN THE MANAGEMENT OF TYPE-II DIABETES MELLITUS AMONG ADULTS ATTENDING GARISSA COUNTY REFERRAL HOSPITAL, KENYA | Abdalla, 2022 | Thesis | Not specified | Kenya | Type 2 Mellitus Diabetes patients | Garissa county referral Hospital | Quasi experimental study | 152 | The intervention group (treatment arm or cases) received a health education package on DM2 comprising education on the disease in terms of causes and risk factors, clinical presentation and complications, screening and diagnosis, management, and control of the disease adapted from the American Diabetes Association. Both the cases (intervention or treatment arm) and the controls received standard diabetic care comprising of medical, nutritional, physical, and counselling care. This standard of care has been shown to improve treatment outcomes, lower the chances of developing complications among DM2 patients, and risk of developing diabetes among prediabetics. The intervention group attended a education program that lasted 6 hours, twice a week for each session, i.e., baseline, and end-term for three weeks over the 6-month study period. The education intervention was facilitated by the principal investigator, and a public health officer research assistant. The educational techniques used in this study included brainstorming, question and answers, scenarios, and group discussion. At baseline and at 6-months after intervention (end-term); physical measurements (height and weight measurements were performed and BMI calculated), and serum samples were collected for analysis of the clinical laboratory markers of diabetes (glucose, glycated haemoglobin, and lipid profile). | Cases (median, 46.0; range, 18.0-84.0 years) and controls (median, 42.0; range, 18.0-81.0 years) | Female: 55,4% intervention group and 34,8 % control group | DM | The effect of group health education. | Socio-demographic and socio-economic assessment indicated that the distribution of age, gender, education levels, marital status, monthly house-hold income, house-hold size, and familial history of diabetes was similar between the cases and controls. The prevalence of type-2 diabetes was 16.6%. Lifestyle evaluation indicated higher rates of smoking a pack of cigarettes daily (71.1% vs. 24.6%) or more than a pack of cigarettes daily (9.6% vs. 17.4%; χ 2 =33.289; df=2; P<0.0001) in the cases and controls, respectively. Overall levels of good knowledge in the cases and controls at baseline (57.8% vs. 72.5%; χ2 =3.816; df=1; P=0.051); and after intervention (88.0% vs. 66.7%; χ2 =11.058; df=1; P=0.001), respectively. Diabetic control marker analysis indicated that only HbA1c levels were significantly lower in the cases compared to controls at baseline (P=0.002). After intervention, BMI (P=0.005), HbA1c (P<0.0001) and fasting glucose (P<0.0001) were significantly lower in the cases relative to the controls. In addition, among the intervention group, the BMI, HbA1c, LDL-cholesterol, HDL-cholesterol, triglycerides and fasting glucose were significantly lower after intervention compared to baseline levels (P<0.01 for all). | Effective adherence strategy |
| Adherence to Diabetes Self-Management and Its Associated Factors Among Adolescents Living with Type 1 Diabetes at Public Hospitals in Addis Ababa, Ethiopia: A Cross-Sectional Study | Geneti et al., 2022 | journal article | 2022 | Addis ababa, Ethiopia | Type 1 Mellitus Diabetes adolescents | Diabetic clinic at public hospitals | Institutional-based quantitative cross sectional study | 422 | Diabetes Self-management: Refers to behaviours such as following adherence to the management of insulin administration, dietary management, management of hypoglycemia, blood glucose testing, and regulation of exercise. The percentage was calculated and categorized as good adherence to diabetes self-management if ‘diabetes self-management profile self-report questionnaire’ scores >50% and poor adherence to diabetes self-management for scores <50% | Mean age of 13.64 years (range: 10-18 years) | Female (56.5%) | DM | Adherence to diabetes self-management (ADSM): Data about self-management activities was collected using the tool called “Validation of a self-report version of the diabetes self-management profile” which was cross-culturally adapted in Hindi in 2020. Its reported internal consistency reliability was α=0.835. This Diabetes Self-Management Profile Self-Report (DSMP-SR) has 24 item instrument that quantifies five areas of diabetes self-management which were insulin administration (4 items), dietary management (6 items), hypoglycemia management (4 items), blood glucose testing (7 items), and exercise (3 items). Then 5-point Likert scale with the anchors 1=never, anchors 2=Almost never, anchors 3=Sometimes, anchors 4=Almost always, anchors 5=always was used. Therefore, those with scores greater than 50% are categorized as having good adherence and those with scores less than or equal to 50% are categorized as having poor adherence. | In this study, a total of 414 diabetic adolescents were interviewed making a 98.1% response rate. About 218 participants (52.7%) had poor adherence to overall diabetes self-management. Self-efficacy (AOR=8.7, 95% CI:1.9-14.1, P=0.005), social support (AOR=4.6, 95%CI:1.5-13.5, P=0.006), age (AOR=0.2, 95%CI:0.1-0.4, P=0.001), good knowledge of the disease (AOR=9.046, 95%CI:3.83-13.5, P=0.000), moderate knowledge (AOR=6.763, 95%CI:2.18-12.921, P=0.001), and time since diagnosis of type 1 diabetes (AOR=0.1, 95%CI:0.02-0.2, P=0.005) were significantly associated with adherence to diabetes self-management. | Effective adherence strategy |
| HIV-and hypertension-related knowledge and medication adherence in HIV seropositive persons with hypertension | Jackson et al., 2022 | Journal article | Not specified | Cross River State, Nigeria | Hypertensive patients who HIV seropositive | University of Calabar Teaching Hospital, HIV clinic. | Descriptive, cross-sectional study | 199 | Intervention: HIV- and hypertension- related knowledge | 46 (IQR 38–58) years | Female (57.3%) | HIV AND HTN | Hypertension-related knowledge compared to HIV-related knowledge and a comparison between ART adherence and antihypertensives adherence. | Participants were predominantly females (57.3%), with a median (IQR) age of 46 (38–58) years; majority were married (67.8%) and employed (60.8%). Participants reported a higher hypertension-related knowledge compared with HIV-related knowledge (63.6% versus 33.3%, Z = −10.263, P < 0.001), but better adherence to antiretroviral medications compared to antihypertensives (100.0% versus 89.3%, Z = −9.118, P < 0.001). Of the 98 participants with documented viral load, 55 (56.1%) had undetectable (<40 copies/ml) values; however, only four (2.0%) of the entire sample had controlled (<140/90 mmHg) blood pressure. | Effective adherence strategy |
| Family support and medication adherence among residents with hypertension in informal settlements of Nairobi, Kenya: a mixed-method study | Xiong et al., 2022 | Abstract | Not specified | Nairobi, Kenya | Hypertensive patients | Two health facilities in informal settlement areas of the Korogocho neighborhood | Mixed-method study | 93 | Family support | Mean age: 57 (SD±14.7) | Female (66%) | HTN | exploration of the relationship between family support and medication adherence among people with hypertension | A total of 93 people participated in the survey (mean age: 57 ± 14.7, 66% female). Most participants reported high family support (82%, n = 76) and suboptimal medication adherence (43% by the Morisky Scale; 76% by the Hill-Bone Scale), with no significant associations between family support and medication adherence. During interviews, many participants reported they lacked health knowledge and education. We suggest that the lack of health knowledge among this population may have contributed to a failure for family support to meaningfully translate into improvements in medication adherence. | Non-effective adherence strategy |
| PEERNaija: A Gamified mHealth Behavioral Intervention to Improve Adherence to Antiretroviral Treatment Among Adolescents and Young Adults in Nigeria | Ahonkhai et al., 2021 | Journal article | Not specified | Nigeria | Adolescents and young adults living with HIV (AYA-HIV) who have poor adherence to ART | Nigerian Institute of Medical Research | Mixed method study approach | 20 | PEERNaija smartphone application: Key features of the guiding application include medication reminders and adherence tracking, refill and appointment reminders, leaderboard and adherence points, internal community supports through discussion forums, peer-to-peer kudos, and community-based resources list. | Age not specified | Not specified | HIV | A peer-based mHealth ART adherence intervention > PEERNaija. | PEERNaija was developed as a gamified Android-based mHealth application to support the behavioral change goal of improving ART adherence among AYA-HIV within Nigeria, a low- and middle- income country (LMIC). Identified via foundational interviews with the target population and review of the literature, key individual (forgetfulness and poor executive functioning), environmental (poor social support) and structural (indirect cost of clinic-based interventions) barriers to ART adherence for AYA-HIV informed application features. Further informed by established behavioral theories and principles, the intervention aimed to improve self-efficacy and self-regulation of AYA-HIV, leverage peer relationships among AYA to incentivize medication adherence (via contingency management, social accountability), provide peer social support through an app-based chat group, and allow for outreach of the provider team through the incorporation of a provider application. Gamification mechanics incorporated within PEERNaija include: points, progress bar, leaderboard with levels, achievements, badges, avatars and targeted behavior change messages. PEERNaija was designed as a tethered mobile personal health record application, sharing data to the widely deployed Open MRS electronic health record application. It also uses the secure opensource Nakama gamification platform, in line with Principles of Digital Development that emphasize use of opensource systems within LMICs. | Effectiveness of adherence strategy is unclear |
| The transient effect of a peer support intervention to improve adherence among adolescents and young adults failing antiretroviral therapy in Harare, Zimbabwe: A randomized control trial | Ndhlovu et al., 2021 | Journal article | 2016-2018 | Zimbabwe | HIV positive adolescents and young adults on ART with virologic failure (VF) | Parirenyatwa Hospital Family Care Clinic-one of the largest referral hospitals in Zimbabwe | Randomized control trial | 212 | Participants enrolled in the intervention arm received SOC as practiced at the PHFCC as well as ‘Zvandiri’ as the intervention. “Zvandiri” is a peer-led model of layered psychosocial support services, delivered through home visits, support groups, clinic visits and mobile health, together with support for caregivers. On enrolment in the study, each participant was referred by the clinic to Africaid’s Zvandiri programme and assigned to a Community Adolescent Treatment Supporter (CATS) living within their own community. CATS are young people (18–24 years old) living with HIV who are recruited, trained, and mentored as peer counsellors. CATS conducted weekly home visits for participants who had consented to be visited, during which they provided information, counselling, adherence monitoring and support. Each CATS delivered weekly WhatsApp messages to their designated participant. The messages provided adherence and clinic reminders and enquired about the participant's well-being. | 18.1 (IQR: 15.1–20.0) years | Female (49.5%) | HIV | The primary outcome of this trial was the proportion of participants who were virologically suppressed (defined as HIV VL of < 1000 copies/mL as per WHO guidelines adopted nationally and being used at that time) in the intervention group compared to SOC at week 36 [25]. | The participants’ median (interquartile range (IQR)) age was 18.1 (IQR: 15.1–20.0) years and half (50.5%, n = 107) were male. At week 24, the proportion of subjects with a detectable viremia was significantly lower in the intervention arm than in the standard of care (SOC) arm (76.0% (n = 79) vs. 89.0% (n = 96), p = 0.013). At Week 36, there remained a difference in the proportion of subjects with a detectable VL between the intervention arm (68.3%, n = 71) and SOC arm (79.6%, n = 86), which was trending towards statistical significance (p = 0.059). There was no difference in the probability of having a detectable VL over time between the intervention and SOC groups (adjusted odds ratio: 1.14, p = 0.439). Baseline HIVDR was observed in 44.0% of the participants in the intervention and 56.0% in the SOC group (p = 0.146). | Non-effective adherence strategy |
| Social networks and barriers to ART adherence among young adults (18–24 years) living with HIV at selected primary health facilities of South-Western Uganda: A qualitative study | Ajuna et al., 2021 | journal article | 2020 | Southwestern Uganda | Young adults living with HIV (YALWH) | 2 Public primary health care facilities: 1 urban and 1 rural | Descriptive qualitative study | 23 | Social networks: Social networks were categorized into three namely: bonding, bridging, and linking networks. Bonding networks were defined as those that involved “strong bonds or intimate relationships with people of the same background” that supported YALWH, such as family members, spouses, friends, and neighbours. Bonding networks were indicated by individuals who were in close contact with the YALWH with whom they engaged in regular visits, talks, collaborative activities; considered confidants for HIV status disclosure and helped YALWH overcome barriers to ART adherence. Bridging networks were defined as networks that involved weaker but more cross-cutting connections with “people of diverse backgrounds” organized in either local, regional, or national level networks of community groups. | Range: 18-24 years | Not specified | HIV | A description of the social networks of YALWH, their barriers to ART adherence, and the perceived role of social networks in overcoming barriers to ART adherence. | Most YALWH belonged to bonding (family, friends, and neighbours), followed by bridging (informal groups), and linking (health professionals) social networks, respectively. Most YALWH, irrespective of gender, had close connections with their mothers or elder sisters. The commonest form of bridging networks was informal community groups that provided financial services, whereas the linking ones comprised health professionals’ directly involved in HIV patient care such as nurses, counsellors, and their affiliates (expert clients or clinic based peer supporters), who occasionally acted as bonding networks. Structural barriers to ART adherence (eg, stigma) were the most cited, followed by medication- (eg, pill burden), and patient-related barriers (eg, non-disclosure of HIV status). Bonding networks were perceived to help overcome patient, medication, and structural barriers to ART adherence. Bridging networks overcame structural and medication-related barriers to ART adherence. Linking networks were perceived to help overcome some health systems and medication-related barriers to ART adherence. | Effective adherence strategy |
| Effectiveness of Mobile Phone Reminders in Improving Adherence and Treatment Outcomes of Patients on Art in Adamawa State, Nigeria: A Ramdomized Controlled Trail | Suru et al., 2021 | journal article | 2017-2018 | Adamawa State, Nigeria | HIV positive adult patients who are on ART | Nigeria (Federal Medical Centre (FMC) Yola, State Specialist Hospital Yola, St Francis Hospital Jambutu in Yola, General hospital Mubi and General hospital Mayo Belwa | Randomized control clinical trial | 244 | Intervention group, Mobile phone reminders: In addition to standard cares the intervention group received a minimum of three (at based line, 3 and month 6) individual counselling sessions with the research assistants lasting an average of 10 minutes per encounter, weekly text message medication reminder, text message reminders 3 days before scheduled clinic appointments and phone call a day to appointment scheduled by the trained research assistants for the period of six months. The clients who did not show up for their medication refills were tracked by the trained research assistant. | 37.7% within the 36 - 45 years age grouping in the intervention group and 39.3% within the 26–35 years age grouping in the control group. | Female: the intervention (64.8%) and control (63.1%) groups | HIV | Outcome is the strength of associations and relationships between the various variables and probability of statistically significant level set. | The response rates in the intervention and control groups were 99% and 96.7% at 3 months; 97.5% and 92.6% at 6 months, respectively. Individual socio-demographic characteristics were not found to be associated with adherence levels in this study. At six months follow up the proportion of the respondents who had good adherence (>95%) was higher (89.1%) and statistically significant (p= 0.001) in the intervention group compared to control group (63.1%) and (p= 0.617). A significantly higher frequency in missed clinic appointments (7.98 vs 1.68) (p=0.024) was noticed in the control group, and a statistically significant increase in the proportion of participants who reported an increase in weight (p=0.001), CD4 cells counts (p=0.001) and decrease in the presence of tuberculosis and other opportunistic infections were observed among patients in the intervention group. | Effective adherence strategy |
| Educational and Psychological Issues Effectiveness of a group diabetes education programme in under-served communities in South Africa: a pragmatic cluster randomized controlled trial | Mash et al., 2014 | Journal article | 2010-2011 | Cape town, South Africa | Type 2 Mellitus Diabetes patients | Public sector community health centres | Pragmatic clustered randomized controlled trial | 1570 | The intervention consisted of four 60-min sessions of group education that focused on understanding diabetes, living a healthy lifestyle, understanding the medication and avoiding complications. Although the training manual anticipated the sessions would last up to 120 min, in reality the sessions lasted up to 60 min. Health promoters recruited from the district health services were trained over a total of 6 days to deliver each session within the facility, using a guiding style of communication based on motivational interviewing principles and skills. Resource materials for group activities were developed for each session and the training manual was published elsewhere 18. The resource materials were made available in English, Afrikaans and Xhosa as necessary. Health promoters discussed the practical implementation during training and each identified a suitable location at their health centre for the group education prior to the intervention. | Mean age: 56.1 (SD±11.6) years | Female: 75,7% control group, 71,5 intervention group | DM | The primary outcome measures were defined as improved diabetes self-care activities, 5% weight loss, and a 1% reduction in HbA1c level. Secondary outcomes were improved diabetes-specific self-efficacy, locus of control, mean blood pressure, mean weight loss, mean waist circumference, mean HbA1c and mean total cholesterol levels, and quality of life. | A total of 422 (59.4%) participants in the intervention group did not attend any education sessions. No significant improvement was found in any of the primary or secondary outcomes, apart from a significant reduction in mean systolic (-4.65 mmHg, 95% CI 9.18 to -0.12; P = 0.04) and diastolic blood pressure (-3.30 mmHg, 95% CI -5.35 to -1.26; P = 0.002). Process evaluation suggested that there were problems with finding suitable space for group education in these under-resourced settings, with patient attendance and with full adoption of a guiding style by the health promoters. | Non-effective adherence strategy |
| Effect of a nutrition education programme on clinical status and dietary behaviours of adults with type 2 diabetes in a resource-limited setting in South Africa: A randomised controlled trial | Muchiri et al., 2016 | Journal article | 2010 - 2011 | North West Province, South Africa | Type 2 Mellitus Diabetes patients | Two community health centres, moretele sub-district | Randomized control trial | 82 | The control group participants received education materials (pamphlet and wall/fridge poster) and continued with the usual medical care at their respective CHC. The intervention group received the same education materials and also participated in an NE programme. The NE programme consisted of three components: (i) the curriculum (eight weekly sessions, 2 to 2·5 h each; ); (ii) follow-up sessions (four monthly meetings and two bi-monthly meetings each lasting 1·5 h); and (iii) vegetable gardening (demonstration of sowing/transplantation of vegetables). The NE sessions were offered in five groups of six to ten participants. The groups were formed on the basis of recruitment time; therefore, the NE sessions were staggered over the study period. The first group commenced in June 2010 and the last group completed in November 2011. Participants were restricted to their clinics as they receive their medication at a particular clinic and the NE sessions were offered at participants’ respective CHC. The total NE programme contact time was 26·5 h per group for the combined weekly and monthly meetings. | Mean age was 58·8 (SD±7·7) years | female: intervention: 87,8%; control: 85,4% | DM | The primary outcome was the change in HbA1c at 6 months. The secondary outcomes were changes in other clinical outcomes (BMI, blood pressure and blood lipids), HbA1c and dietary behaviours at 12 months. | Differences in HbA(1c) (primary outcome) were -0·64 % (P=0·15) at 6 months and -0·63 % (P=0·16) at 12 months in favour of the intervention group. Starchy-food intake was significantly lower in the intervention group, 9·3 v. 10·8 servings/d (P=0·005) at 6 months and 9·9 v. 11·9 servings/d (P=0·017) at 12 months. Median energy intake was significantly lower in the intervention group at 12 months (5988 v. 6946 kJ/d, P=0·017). No significant group differences in BMI, lipid profile, blood pressure and intakes of macronutrients, vegetables and fruits were observed. | Effective adherence strategy |
| Short- and long-term efficacy of modified directly observed antiretroviral treatment in Mombasa, Kenya: a randomized trial | Sarna et al., 2008 | Journal article | 2003-2004 | Mombasa, Kenya | HIV positive patients | A provincial referral hospital (n = 167); a private, not-for-profit clinic (n = 59); and a district hospital (n = 8). | Randomized control clinical trial | 234 | m-DOT (24 weeks of twice weekly health center visits for nurse-observed pill ingestion, adherence support, and medication collection | Control: 37 (SD±7.8) MDOT: 37.3 (SD±8.0) | Female: control:75%; intervention:74% | HIV | Levels of adherence in the 2 groups was compared in the first 24 weeks to assess effectiveness of the intervention and then compared during weeks 25-48 and week 72 to detect any sustained effects on adherence. Secondary outcomes were evaluated as follows: virological suppression, increases in CD4 cell counts, and changes in weight and BMI. | During weeks 1-24, 9.1% (9/99) of m-DOT participants reported missing doses compared with 19.1% (20/105) of controls (P = 0.04) and 96.5% (517/571) of m-DOT pill-count measures were ≥95% compared with 86.1% (445/517) in controls [adjusted odds ratio = 4.4; 95% confidence interval (CI) = 2.6 to 7.5; P < 0.001. Adherence with m-DOT was 4.8 times greater (95% CI = 2.7 to 8.6; P < 0.001) with adjustment for depression and HIV-related hospitalization. In weeks 25-48, adherence with m-DOT (488/589) was similar to controls (507/630). Viral suppression at 48 weeks was 2.0 times (95% CI = 0.8 to 5.2; P = 0.13) as likely in m-DOT participants as controls. M-DOT patients had larger body mass index increases at 24 weeks (2.2 vs 1.4 kg/m3; P = 0.014). Viral suppression was more likely at week 48 (21/25 vs 13/22; P = 0.057) and week 72 (27/30 vs 15/23; P = 0.027) among depressed participants receiving m-DOT. | Effective adherence strategy |
| Supporting Adolescents With HIV in South Africa Through an Adherence-Supporting App: Mixed Methods Beta-Testing Study | Mulawa et al., 2023 | Journal article | August 2021 to December 2021 | Cape Town, South Africa | Adolescents (15-19 years) with HIV | unnamed teaching hospital linked to several HIV clinics in Cape Town, South Africa | Mixed Methods | 14 | Masakhane Siphucule Impilo Yethu (MASI; Xhosa for “Let's empower each other and improve our health”), a smartphone app–delivered intervention to improve treatment adherence among adolescents with HIV in South Africa. MASI is a comprehensive, mobile app–delivered intervention designed to foster connection among users and provide engaging resources related to health, life skills, relationships, and well-being. MASI uses a strengths-based approach that acknowledges and bolsters individual and social network–level resources and assets | Mean age 18 (SD=1) | 7 females, and 7 males | HIV | To explore engagement with MASI features that allowed the posting of user-generated content (ie, Forum and Ask the Expert), authors categorized participants based on their level of engagement (eg, posting, commenting or replying, liking or favoriting, or reading content). | Participants logged into MASI an average of 24.1 (range 10-75) times during the study period. The mean System Usability Scale score was 69.5 (SD 18), which is considered slightly above average for digital health apps. Thematic analysis of qualitative results revealed generally positive experiences across MASI features, although opportunities to refine the app and intervention delivery were identified. Initial usability of MASI was high, and participants described having a generally positive experience across MASI features. Systematically analyzing paradata and using the interview findings to explore participant experiences allowed us to gain richer insights into patterns of participant engagement, enabling our team to further enhance MASI | Effective adherence strategy |
| Nutritional Treatment Outcomes of Therapeutic Feeding Program and Its Predictors Among HIV Patients at Hawassa University Comprehensive Specialized Hospital, Hawassa City, Sidama, Southern Ethiopia: A Retrospective Cohort Study | Tadesse et al., 2023 | Journal article | March 1, 2015 to February 30, 2021 | Hawassa city, Ethiopia | Adult HIV-positive patients who are on ART | Adult ART clinic of Hawassa University Comprehensive Specialized Hospital | Retrospective Cohort Study | 419 | The food intervention program is one of the approaches that target malnutrition among HIV-infected individuals and their exposed family members through nutritional screening, counseling, and care. Food by prescription is a program that provides food and nutritional care to malnourished HIV-positive individuals as a therapeutic and supplementary feeding package at health facilities. The objective of the program is to offer energy-rich and nutrient-dense food products along with nutrition evaluation, counseling, and care to people living with HIV who are malnourished or at threat of malnutrition | median age=35 years (IQR 29–42). | females (59.2%) | HIV | Outcome of the treatment with food by prescription for 3–6 months among HIV-positive patients recovered, unrecovered, defaulted or died. | The proportion of adult HIV patients who recovered from malnutrition after they were enrolled in the food by prescription therapy was 53.0%. The variables found to have an association with good nutritional treatment outcomes of food by prescription in the final model are being female (AHR= 3.38, 95% CI: 2.15, 5.32), secondary education (AHR = 2.16, 95% CI:1.11, 4.18), tertiary education (AHR = 3.75, 95% CI: 1.66, 8.48), SAM (AHR = 0.20; 95% CI: 0.12, 0.35), and HAART (AHR = 2.70, 95% CI: 1.50, 4.86). Having Severe Acute Malnutrition (SAM) at baseline nutritional assessment (AHR = 3.42, 95% CI; 2.81, 5.15), taking ART for more than 12 months (AHR = 0.26, 95% CI: 0.13, 0.84) and starting HAART immediately after testing positive (AHR = 0.26, 95% CI: 0.13, 0.84) are significantly associated with nutritional treatment failure. | Effective adherence strategy |
| Integrated management of HIV, diabetes, and hypertension in sub-Saharan Africa (INTE-AFRICA): a pragmatic cluster randomised, controlled trial | Kivuyo et al., 2023 | Journal article | June 30, 2020, and April 1, 2021 | 17 health facilities in Uganda and 15 health facilities in Tanzania | Patients living with confirmed HIV, diabetes, or hypertension, were aged 18 years or older | Primary healthcare facilities | Pragmatic cluster-randomised, controlled trial | 7028 | In the integrated care group participants with either HIV, hypertension, or diabetes attended a single clinic. They shared the same registration and waiting area, were managed by the same health-care workers, and used the same pharmacy. Their medical records were stored in folders with a similar appearance, to ensure a consistent approach to care across these conditions. Their laboratory services were also integrated | Integrated care: Participants with diabetes, hypertension, or both: Mean (SD)=60.1 (12.7) Standard care: Participants with diabetes, hypertension, or both: Mean (SD)=57.7 (12.2) Integrated care: Participants with HIV alone: Mean (SD)=42.6 (11.2) Standard care: Participants with HIV alone: Mean (SD)=42.7 (10.8) | Among participants with diabetes, hypertension, or both, 2298 (75·8%) of 3032 were female and 734 (24·2%) of 3032 were male. Of participants with HIV alone, 2365 (70·3%) of 3365 were female and 1000 (29·7%) of 3365 were male | HIV, HTN, and DM | The study had two coprimary endpoints: retention in care for participants with either diabetes or hypertension (tested for the superiority of integrated care) and plasma viral load suppression for those with HIV (tested for noninferiority). 12 Retention in care was chosen as a primary endpoint, as the loss from care is probably the biggest determinant of mortality among people with diabetes or hypertension, and the immediate problem that policy makers wanted to address. Secondary endpoints were blood pressure and glycaemia control, rates of retention for all patients, and costs of care. | Follow-up lasted for 12 months. Among participants with diabetes, hypertension, or both, the proportion alive and retained in care at study end was 1254 (89·0%) of 1409 in integrated care and 1457 (89·8%) of 1623 in standard care. The risk differences were –0·65% (95% CI –5·76 to 4·46; p=0·80) unadjusted and –0·60% (–5·46 to 4·26; p=0·81) adjusted. Among participants with HIV, the proportion who had a plasma viral load of less than 1000 copies per mL was 1412 (97·0%) of 1456 in integrated care and 1451 (97·3%) of 1491 in standard care. The integrated chronic care management was associated with a high level of retention in care for people with diabetes or hypertension, although not superior to vertical management, and that integration did not adversely affect the rate of viral suppression among people with HIV. In sub-Saharan Africa, integrated chronic care services could achieve a high standard of care for people with diabetes or hypertension without adversely affecting outcomes for people with HIV. This trial demonstrated that integrated chronic care management in a sub-Saharan African healthcare setting was associated with a high level of retention in care for people with diabetes or hypertension, although not superior to vertical management, and that integration did not adversely affect the rate of viral suppression among people with HIV. | Effective adherence strategy |
| A Structural Equation Model of the Impact of a Family-Based Economic Intervention on Antiretroviral Therapy Adherence Among Adolescents Living With HIV in Uganda | Kizito et al., 2023 | Journal article | January 2014 and December 2015 | Uganda | Adolescents (10-16 years) living with HIV | Masaka, Uganda | Cluster-randomized controlled trial | 702 | 1. Child development account. A long-term savings account was opened for each study participant, in which they saved their money. The Suubi + Adherence study made the initial deposit of money on the account. In addition, for each monthly deposit that the participant saved in the account, the study added an equal amount. These accounts were retained by the participants upon the termination of the study. Various studies have shown the positive role of child development accounts in improving adolescent mental health functioning. 2. Workshops. The participants and their families participated in microenterprise workshops where they were trained on financial management and starting family businesses. A total of four sessions were provided per participant. 3. The participants received mentorship through attending 12 educational sessions. These sessions addressed various topics, including setting short- and long-term goals, averting risk-taking behaviors, and business development. | mean age=12 years, | 56% were female | HIV | The primary outcome was ART adherence, measured using a six-item self-report measure, three of which are included in the Wilson’s three-item self-reported adherence measureme. Adherence data were collected at 24, 36, and 48 months postintervention initiation, with Cronbach’s alpha ranging from 0.43 at baseline to 0.49 at 48 months | Economic empowerment interventions improve adherence, by improving mental health functioning. At 36 (model 2) and 48 months (model 3), the intervention had a significant indirect effect on ART adherence [B = 0.069, Β = 0.039 (95% confidence interval [CI]: 0.005–0.074)], and [B = 0.068, β = 0.040 (95% CI: 0.010–0.116)], respectively. In both models, there was a specific mediation effect through mental health [B = 0.070, β = 0.040 (95% CI: 0.007–0.063)], and [B = 0.039, β = 0.040 (95% CI0.020–0.117)]. Overall, 49.1%, 90.7%, and 36.8% of the total effects were mediated in models, 1, 2, and 3, respectively. | Effective adherence strategy |
| Effect of the Friendship Bench intervention on antiretroviral therapy outcomes and mental health symptoms in rural Zimbabwe: A cluster randomized trial | Haas et al., 2023 | Journal article | Between Oct 5, 2018, and Dec 19, 2019 | Bikita, a rural district in Masvingo Province, Zimbabwe | Men and non-pregnant women aged 18 years or older who are HIV positive and who received first-line ART for at least 6 months | Sixteen public health care facilities in Bikita, a rural district in Masvingo Province, about 300 km south of Harare. | Pragmatic cluster trial with block randomization of health facilities. | 516 | Participants in the intervention arm were offered the Friendship Bench (FB) intervention in addition to an enhanced standard of care (SC). Participants in the control arm received SC only. The FB intervention consisted of weekly individual counselling sessions over six weeks and optional peer-led group support. Trained lay health workers delivered sessions, following a structured approach to identify problems, including adherence issues, and foster a positive attitude towards resolving them. After four sessions, participants were invited to join a peer-led group activity where they were trained in income-generating skills (e.g. producing bags from recycled plastic). The FB intervention is described in detail elsewhere.SC consisted of nurse-led brief counselling, education, and support regarding CMD. Prescription of an antidepressant (fluoxetine) or referral to a psychiatric facility followed standard operating procedures (see Supplement 3). The nurses were trained in managing mental, neurological and substance use disorders | Mean age=45.6 years (SD 10.9) | 84.9% were females | HIV | The primary outcome was Medication Event Monitoring System (MEMS) mean adherence between 2-6 months of follow-up. Secondary outcomes included mean adherence between 1-12 months, change from baseline SSQ-14 and Patient Health Questionnaire (PHQ-9) score at 3, 6, 9, and 12 months and change in viral load suppression (viral load <1000 copies per mL) at months 6 and 12. | In the Friendship Bench group, 88.1% of participants attended all six individual counselling sessions. Rates of adherence (>85%) and virologic suppression (>90%) were high in both groups. The intervention had no statistically significant effect on adherence or viral suppression. Declines in SSQ-14 scores from baseline to 3 months (-1.65, 95% CI -3.07 to -0.24), 6 months (-1.57, 95% CI -2.98 to -0.15), and 9 months (-1.63, 95% CI -3.05 to -0.22) were greater in the Friendship Bench than the standard care group (p<0.05). There were no differences in the decline in the SSQ-14 scores from baseline to 12 months and in declines in PHQ-9 scores from baseline to 3, 6, 9, and 12 months. | Non-effective adherence strategy |
